# SARS-CoV-2 variant Alpha has a spike-dependent replication advantage over the ancestral B.1 strain in human cells with low ACE2 expression

**DOI:** 10.1371/journal.pbio.3001871

**Published:** 2022-11-16

**Authors:** Daniela Niemeyer, Saskia Stenzel, Talitha Veith, Simon Schroeder, Kirstin Friedmann, Friderike Weege, Jakob Trimpert, Julian Heinze, Anja Richter, Jenny Jansen, Jackson Emanuel, Julia Kazmierski, Fabian Pott, Lara M. Jeworowski, Ruth Olmer, Mark-Christian Jaboreck, Beate Tenner, Jan Papies, Felix Walper, Marie L. Schmidt, Nicolas Heinemann, Elisabeth Möncke-Buchner, Morris Baumgardt, Karen Hoffmann, Marek Widera, Tran Thi Nhu Thao, Anita Balázs, Jessica Schulze, Christin Mache, Terry C. Jones, Markus Morkel, Sandra Ciesek, Leif G. Hanitsch, Marcus A. Mall, Andreas C. Hocke, Volker Thiel, Klaus Osterrieder, Thorsten Wolff, Ulrich Martin, Victor M. Corman, Marcel A. Müller, Christine Goffinet, Christian Drosten

**Affiliations:** 1 Institute of Virology, Campus Charité Mitte, Charité — Universitätsmedizin Berlin, Berlin, Germany; 2 German Center for Infection Research, associated partner Charité, Berlin, Germany; 3 Berlin Institute of Health, Berlin, Germany; 4 Institut für Virologie, Freie Universität Berlin, Berlin, Germany; 5 Leibniz Research Laboratories for Biotechnology and Artificial Organs (LEBAO), Department of Cardiothoracic, Transplantation and Vascular Surgery, REBIRTH — Center for Translational Regenerative Medicine, Biomedical Research in Endstage and Obstructive Lung Disease Hannover (BREATH), German Center for Lung Research (DZL), Hannover Medical School, Hannover, Germany; 6 Department of Infectious Diseases and Respiratory Medicine, Charité — Universitätsmedizin Berlin, Berlin, Germany; 7 Institute for Medical Virology, University Hospital Frankfurt, Goethe University, Frankfurt am Main, Germany; 8 Institute of Virology and Immunology, Bern, Switzerland; 9 Department of Pediatric Respiratory Medicine, Immunology and Critical Care Medicine, Charité - Universitätsmedizin Berlin, Berlin, Germany; 10 Unit 17 “Influenza and other Respiratory Viruses", Robert Koch Institute, Berlin, Germany; 11 Institute of Pathology, Charité - Universitätsmedizin Berlin, corporate member of Freie Universität Berlin, Humboldt - Universität zu Berlin, Berlin, Germany; 12 BIH Bioportal Single Cells, Berlin Institute of Health at Charité Universitätsmedizin Berlin, Berlin, Germany; 13 German Center for Infection Research, DZIF, Braunschweig, Germany; 14 Fraunhofer Institute for Molecular Biology and Applied Ecology (IME), Branch Translational Medicine and Pharmacology, Frankfurt am Main, Germany; 15 Institute of Medical Immunology, Charité — Universitätsmedizin Berlin, Corporate Member of Freie Universität Berlin and Humboldt Universität zu Berlin, Berlin, Germany; 16 German Centre for Lung Research (DZL), associated partner Charité, Berlin, Germany; 17 Department of Infectious Diseases and Public Health, Jockey Club College of Veterinary Medicine and Life Sciences, City University of Hong Kong, Kowloon Tong, Hong Kong; 18 Labor Berlin – Charité Vivantes GmbH, Berlin, Germany; University of Wisconsin-Madison, UNITED STATES

## Abstract

Epidemiological data demonstrate that Severe Acute Respiratory Syndrome Coronavirus 2 (SARS-CoV-2) variants of concern (VOCs) Alpha and Delta are more transmissible, infectious, and pathogenic than previous variants. Phenotypic properties of VOC remain understudied. Here, we provide an extensive functional study of VOC Alpha replication and cell entry phenotypes assisted by reverse genetics, mutational mapping of spike in lentiviral pseudotypes, viral and cellular gene expression studies, and infectivity stability assays in an enhanced range of cell and epithelial culture models. In almost all models, VOC Alpha spread less or equally efficiently as ancestral (B.1) SARS-CoV-2. B.1. and VOC Alpha shared similar susceptibility to serum neutralization. Despite increased relative abundance of specific sgRNAs in the context of VOC Alpha infection, immune gene expression in infected cells did not differ between VOC Alpha and B.1. However, inferior spreading and entry efficiencies of VOC Alpha corresponded to lower abundance of proteolytically cleaved spike products presumably linked to the T^716^I mutation. In addition, we identified a bronchial cell line, NCI-H1299, which supported 24-fold increased growth of VOC Alpha and is to our knowledge the only cell line to recapitulate the fitness advantage of VOC Alpha compared to B.1. Interestingly, also VOC Delta showed a strong (595-fold) fitness advantage over B.1 in these cells. Comparative analysis of chimeric viruses expressing VOC Alpha spike in the backbone of B.1, and vice versa, showed that the specific replication phenotype of VOC Alpha in NCI-H1299 cells is largely determined by its spike protein. Despite undetectable ACE2 protein expression in NCI-H1299 cells, CRISPR/Cas9 *knock-out* and antibody-mediated blocking experiments revealed that multicycle spread of B.1 and VOC Alpha required ACE2 expression. Interestingly, entry of VOC Alpha, as opposed to B.1 virions, was largely unaffected by treatment with exogenous trypsin or saliva prior to infection, suggesting enhanced resistance of VOC Alpha spike to premature proteolytic cleavage in the extracellular environment of the human respiratory tract. This property may result in delayed degradation of VOC Alpha particle infectivity in conditions typical of mucosal fluids of the upper respiratory tract that may be recapitulated in NCI-H1299 cells closer than in highly ACE2-expressing cell lines and models. Our study highlights the importance of cell model evaluation and comparison for in-depth characterization of virus variant-specific phenotypes and uncovers a fine-tuned interrelationship between VOC Alpha- and host cell-specific determinants that may underlie the increased and prolonged virus shedding detected in patients infected with VOC Alpha.

## Abstract 2

Epidemiological data show that SARS-CoV-2 variants of concern Alpha and Delta are more transmissible, infectious and pathogenic than previous variants, but their phenotypic properties remain understudied. A functional study of the Alpha variant identifies a single bronchial cell line that reflects its enhanced in vivo transmissibility.

## Introduction

Since its emergence, Severe Acute Respiratory Syndrome Coronavirus 2 (SARS-CoV-2) has genetically diversified, giving rise to variants with altered phenotypic properties [1]. In May 2021, WHO announced a scheme for labeling SARS-CoV-2 lineages with evidence for increased transmissibility, severity, and escape from immunity [2]. The B.1.1.7 lineage was labeled VOC Alpha as it was the first variant of concern (VOC) [3]. First detected in the United Kingdom in September 2020 [4], it shows a 50% to 100% higher reproduction number than previously circulating viruses [5]. Furthermore, the estimated hazard of death associated with VOC Alpha is 61% higher than with preexisting variants [6]. Delta was identified in India in October 2020 and labeled VOC Delta in May 2021. After causing infection waves with increased mortality in many countries during 2021, it is now rarely detected and VOC Omicron subvariants BA.4 and BA.5 predominate. Despite a rapid succession of VOCs, functional properties of even the early VOC Alpha remain understudied.

Genetic hallmarks of VOC Alpha comprise a set of mutations resulting in nonsynonymous changes within the spike gene including deletion of amino acids 69, 70, and 144 in the amino-terminal domain, an N^501^Y exchange in the receptor-binding domain, an A^570^D exchange in subdomain 1, P^681^H, and T^716^I exchanges in the proximity of the furin cleavage site and the S1/S2 domain junction, as well as S^982^A and D^1118^H in the S2 domain. In addition to SNPs in ORF1ab and nucleoprotein, ORF8 in variant VOC Alpha contains a premature stop codon. The functional role of ORF8 in SARS-CoV-2 is unclear. A variant with deleted ORF8 that circulated in Singapore during spring 2020 showed limited evidence for changes in in vitro transcription profile [7] and clinical outcome [8].

To date, only few studies have identified potential functional correlates for the enhanced transmission and pathogenicity particularly of VOC Alpha. In 1 study, VOC Alpha-infected lung epithelial cells produced increased quantities of subgenomic RNAs and elevated ORF6 and ORF9b protein levels, which were proposed to be linked to more efficient suppression of cellular innate immune response [9]. Studies of viral loads demonstrated that VOC Alpha- and Delta-infected individuals shed viral RNA at increased levels [10,11] and for prolonged time [12,13]. Individual mutations in VOC Alpha spike have been investigated with regard to protein structure [14], in vitro ACE2-binding [15], spike processing [16,17] and stability [18], as well as fitness [18]. In contrast to VOC Delta [19–21], VOC Alpha displays only modest, if any, alteration of sensitivity to neutralizing antibodies [22–24], suggesting a limited contribution of antibody-dependent immune escape to the observed phenotype of VOC Alpha. In vitro and in vivo replication of VOC Alpha was found to differ depending on the model used. Some epithelial cell cultures and hamster models showed equal, slightly superior, or inferior replication for VOC Alpha [25–28], while VOC Alpha generally exhibited marginally superior replication in primates and ferrets [25,29]. However, nonhuman models may be limited in their capability to reflect human-adaptive processes that occur in a virus establishing endemicity in humans. Here, we studied the replication of VOC Alpha in different cell and organ models as well as dwarf hamsters and identified a human cell line that reflects the fitness advantage of VOC Alpha over B1.

## Results

### Compared to SARS-CoV-2 B.1, VOC Alpha shows similar or inferior replication kinetics in immortalized cell lines

**Fig 1:**
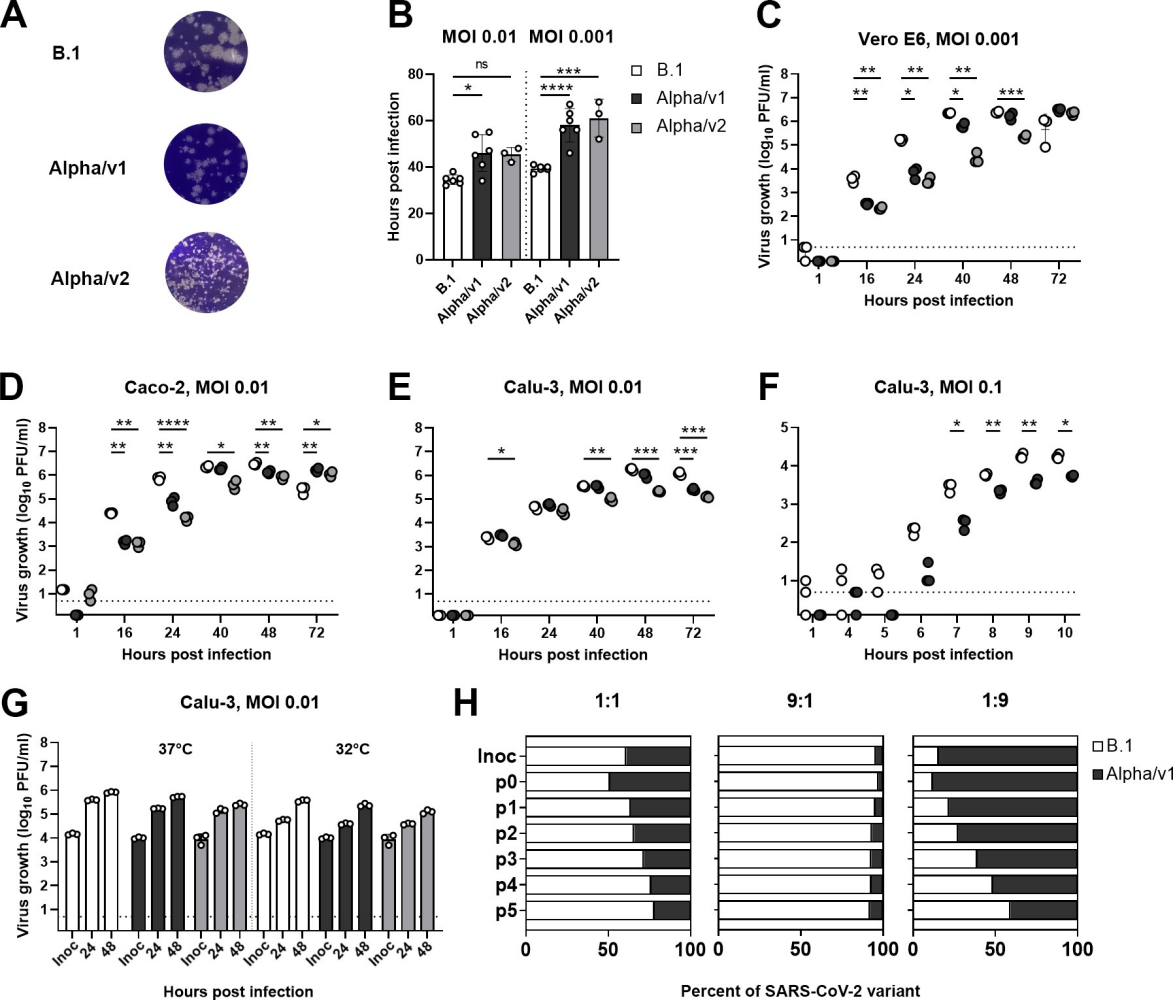
Compared to SARS-CoV-2 B.1, VOC Alpha shows similar or inferior replication kinetics in immortalized cell lines. (**A**) Plaque morphology on Vero E6 cells, which were infected with 1:100 diluted (B.1 and VOC Alpha/v1) or undiluted (VOC Alpha/v2) supernatants of infected Vero E6 cells. (**B**) Vero E6 cells were infected at the indicated MOI and onset of CPE was monitored by live cell imaging until 70 hours postinfection. (**C**-**E**) Virus growth was quantified in Vero E6 (**C**), Caco-2 (**D**), and Calu-3 (**E**) cells infected at indicated MOIs. Supernatant collected at the respective time points was titrated by plaque assay. Growth kinetic experiments in Vero E6 and Caco-2 cells were each performed in triplicates. One representative experiment out of 2 is shown for Calu-3 cells. (**F**) Virus growth in Calu-3 cells infected at an MOI of 0.1 was quantified at early time points after infection. (**G**) Virus growth kinetics in Calu-3 cells at 37°C (left) and 32°C (right). (**H**) Competition assay. Calu-3 cells were infected with a mixture of B.1 and VOC Alpha/v1 at indicated ratios (B.1: VOC Alpha/v1 ratio of 1:1, 9:1, and 1:9) with a total infectious dose of 10,000 PFU (corresponding to an MOI of 0.04). After serial passaging, viral RNA from the supernatant was isolated, sequenced, and the relative proportion of B.1- and VOC Alpha-corresponding sequences (discriminated by a mutation in nsp12; see also S1 Fig) was plotted. Data show arithmetic means of 1 experiment performed in triplicates. Dashed horizontal lines indicate the lower detection limit of the plaque assay. CPE, cytopathogenic effect; Inoc., Inoculum; MOI, multiplicity of infection; PFU, plaque-forming units; p0-p5, passage 0–passage 5; SARS-CoV-2, Severe Acute Respiratory Syndrome Coronavirus 2; VOC, variant of concern. See S1 Data.

We first studied virus replication kinetics in a panel of immortalized cell lines. Compared to an early lineage (Non-VOC) B.1 strain carrying the D^614^G mutation (BavPat1/2020; [30]), 2 different VOC Alpha isolates showed smaller plaque size 3 days postinfection (Fig 1A) and a delayed manifestation of cytopathogenic effect (CPE) in Vero E6 cells (Fig 1B and S1 and S2 Movies) accompanied by a delay in infectious particle production during the initial 40 hours postinfection of Vero E6 cells (Fig 1C).

Unlike Vero E6 cells, Caco-2 and Calu-3 cells express the transmembrane protease serine subtype 2 (TMPRSS2) and are capable of producing type I interferons (IFNs). They supported the growth of B.1. equally or more efficiently than VOC Alpha (Fig 1D and 1E). Interestingly, VOC Alpha production was particularly delayed in the very early phase of replication (Fig 1F).

Cultivation of infected cells at 32°C in order to represent the temperature in the upper respiratory tract did not alter relative replication efficiencies (Fig 1G). Under competitive passaging in Calu-3 cells, B.1 outcompeted VOC Alpha, even when the starting inoculum contained a 9-fold excess of VOC Alpha (Figs 1H and S1). In sum, immortalized cell models fail to reflect a growth advantage of VOC Alpha expected upon its enhanced transmissibility and pathogenicity in vivo.

### Absence of detectable fitness advantages of VOC Alpha in primary human respiratory cells, organoids, and hamsters

**Fig 2:**
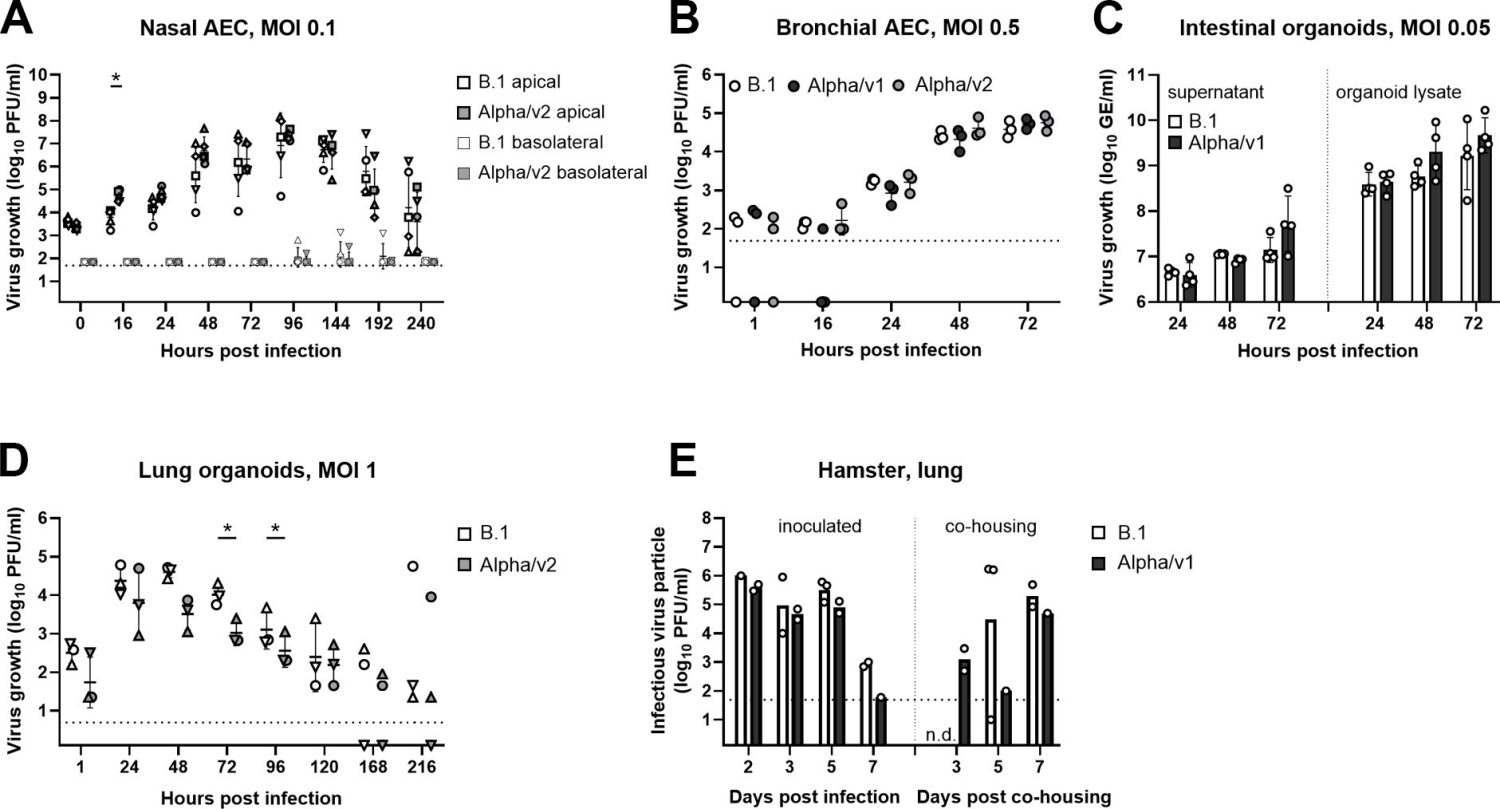
Absence of detectable fitness advantages of VOC Alpha in primary human respiratory cells, organoids, and hamsters. (**A**) Virus growth kinetics were performed in infected hNAECs (MOI 0.1). Samples were collected from the apical and basal side at indicated time points and titrated by plaque assay. *n* = 3 biological replicates. (**B**) Virus growth kinetics was conducted in infected bronchial AEC (MOI 0.5). Samples were collected from the apical side and titrated by plaque assay. Data are derived from 1 experiment conducted in triplicates. (**C**) Intestinal organoids were infected (MOI 0.05) and viral load in supernatant (left) and organoid lysates (right) was quantified at indicated time points by E-gene-specific quantitative RT-PCR. Data are derived from 4 independent experiments. (**D**) Virus replication was monitored in infected lung organoids (MOI 1). Samples harvested at indicated time points were titrated by plaque assay. Data are derived from 3 independent experiments. (**E**) Dwarf hamsters were intranasally infected (100,000 PFU) and infectious virus particles from lung homogenates were quantified using plaque assay (left). Donor hamsters were cohoused with naive animals and transmission efficiency was determined from lung homogenates at the indicated time points (right). *n* = 1–3 animals per experimental condition. Dotted horizontal lines indicate the lower detection limit of the plaque assays. AEC, airway epithelial culture; GE, genome equivalents; hNAEC, human nasal airway epithelial culture; MOI, multiplicity of infection; n.d., not detected; PFU, plaque-forming units; RT-PCR, real-time PCR; VOC, variant of concern. See S1 Data.

To detect potential variant-specific differences that may not become evident in immortalized cell lines, we studied more complex, physiologically relevant, primary human models of mucosal infection. Infection experiments in differentiated air–liquid interface cultures of human nasal (Fig 2A), human bronchial (Fig 2B) airway epithelial cultures (AECs), as well as epithelial intestinal organoids (Fig 2C) failed to reveal a growth advantage for VOC Alpha isolates compared to B.1. Growth of VOC Alpha was even slightly inferior to B.1 in adult stem cell–derived human lung organoids (Fig 2D). Production of both viruses in the lung of infected dwarf hamsters was indistinguishable. In addition, virus production in the lungs of contact animals cohoused with infected animals did not show variant-specific quantitative differences in preliminary analyses (Fig 2E).

### VOC Alpha and B.1 share efficient prevention of induction of innate immunity despite VOC Alpha-specific enhanced production of viral subgenomic RNA transcripts

In accordance with reports by others [24,31,32], B.1 infection- and vaccination-induced antibodies efficiently neutralized both B.1 and VOC Alpha in a plaque reduction neutralization test, whereas they failed to effectively neutralize VOC Beta (S2A Fig). Interestingly, ACE2 binding of the VOC Alpha RBD, which differs from that of B.1 at only 1 position (N^501^Y), was only slightly less sensitive to inhibition by antibodies from vaccinees and/or nonvaccinated patients infected before the emergence of VOC Alpha than by antibodies from nonvaccinated VOC Alpha-infected patients (S2B Fig), suggesting little degree of immune escape associated with VOC Alpha. In contrast, VOC Beta RBD was clearly more resistant to ACE2 binding inhibition than B.1 and VOC Alpha RBDs, confirming immune escape associated with VOC Beta.

**Fig 3:**
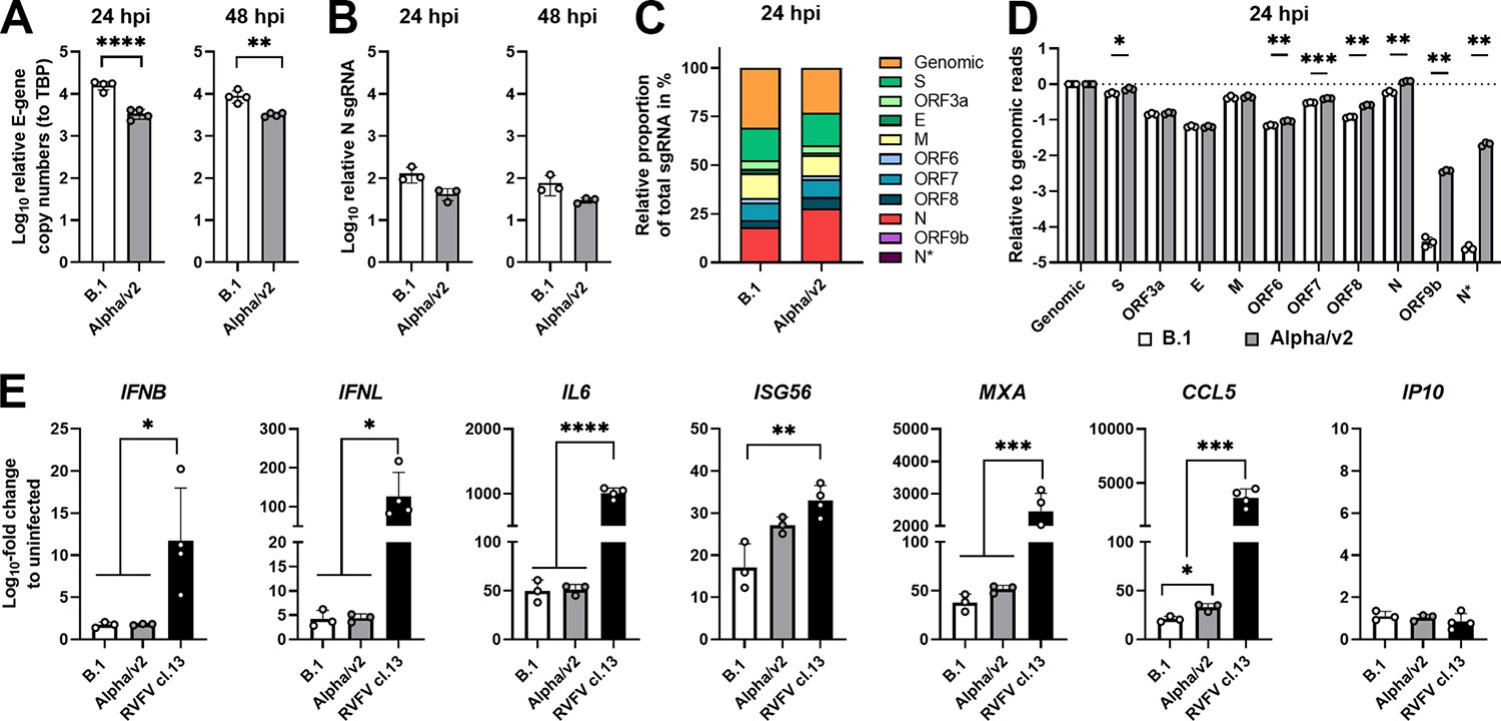
VOC Alpha and B.1 share efficient prevention of induction of innate immunity despite VOC Alpha-specific enhanced production of viral subgenomic RNA transcripts. Calu-3 cells were infected with B.1 or VOC Alpha (MOI of 2) and cell lysates were generated either 24 or 48 hours postinfection, following total RNA extraction. Samples were generated in 4 individual experiments each conducted in quadruplicates. (**A**) Expression of cell-associated *envelope* was determined in Calu-3 at 24 and 48 hours postinfection by Q-RT-PCR. TBP was used for normalization. (**B**) Expression of cell-associated sgN in Calu-3 cells at 24 and 48 hours postinfection was determined by Q-RT-PCR. TBP was used for normalization. (**C**) RNA-seq analysis was conducted from total cell lysates that were obtained 24 hours postinfection to quantify sgRNA proportions in infected cells. Stacked bars depict the relative proportion of total sgRNA in Calu-3 cells mapping to each viral sgRNA from the RNA-seq dataset. (**D**) Canonical, as well as ORF9b and N* sgRNAs were quantified from the RNA-seq dataset. Data were normalized to the respective B.1- or VOC Alpha-specific genomic reads. (**E**) Expression of the indicated genes was determined by Q-RT-PCR. Shown is the mean fold change +/− SD. RVFV cl.13, which is devoid of its IFN antagonist NSs, was included as an induction control for the expression of *IFNs* and *ISGs*. IFN, interferon; MOI, multiplicity of infection; Q-RT-PCR, quantitative real-time PCR; RVFV cl.13, Rift Valley Fever Virus clone 13; sgN, subgenomic nucleocapsid; sgRNA, subgenomic RNA; TBP, TATA-binding protein; VOC, variant of concern. See S1 Data.

The recently identified property of VOC Alpha to induce increased expression of subgenomic RNAs encoding nucleocapsid, ORF6, and ORF9b (as compared to B.1 strains) has been proposed to cause superior ability to prevent or evade cell-intrinsic immunity [33]. Following a similar approach, we found that quantities of genomic RNA were significantly reduced during VOC Alpha infection as compared to B.1. infection (16% reduction at 24 hours postinfection and 11% reduction at 48 hours postinfection; Fig 3A). However, expression of nucleocapsid-encoding subgenomic RNA (sgRNA) was similar between the 2 virus infections (Fig 3B). For better comparability of our study with [9], we performed RNA-seq analysis to quantify global sgRNA proportions in infected cells. As previously observed, the amount of genomic RNAs was reduced by 25% in VOC Alpha- as compared to B.1-infected cells (Fig 3C). Abundance of spike, ORF6, and ORF7 sgRNAs was significantly but very weakly up-regulated (up to 1.3-fold relative to B.1 infection). Levels of nucleocapsid and ORF8 sgRNAs were enhanced 2-fold in the context of VOC Alpha- as compared to B.1 infection. In contrast, ORF9b and N* subgenomic RNAs were 94- and 810-fold increased in VOC Alpha-infected cells (Fig 3D). Of note, relative read counts of ORF9b and N* subgenomic RNAs represented only 0.003% (average absolute read count: 138 transcripts) and 0.007% (average absolute read count: 790 transcripts) of total reads (average absolute read count: 11 million transcripts) in VOC Alpha-infected cells (S3A Fig).

To determine if enhanced expression of these sgRNAs contributes to a more efficient innate immune evasion by VOC Alpha in infected Calu-3 cells, we determined gene expression of IFN-, pro-inflammatory cytokine-encoding and IFN-stimulated genes (ISGs). Infection at multiplicity of infection (MOI) of 2 resulted in 25% and 32% nucleocapsid-positive cells in B.1 and VOC Alpha-infected Calu-3 cells, respectively (S3B and S3C Fig). Expression of *IFNB*, *IFNL*, *IL6*, and *IP10* was induced to similar levels by both variants (Fig 3E). Interestingly, VOC Alpha infection displayed a trend to induce slightly higher levels of *ISG56*, *MXA*, and *CCL5* expression than B.1 (Fig 3E), arguing against the idea that the increase of VOC Alpha-derived sgRNA transcription is linked to stronger suppression of the innate immune response. In conclusion, induction of all tested innate immunity-related genes was efficiently suppressed in B.1- as well as VOC Alpha-infected Calu-3 cells, whereas a Rift Valley Fever Virus clone 13 (RVFV cl.13) control virus infection potently induced their expression, as expected. Similarly, in the ACE2/TMPRSS2-reconstituted lung epithelial cell line A549, expression of subgenomic nucleocapsid RNA and innate immune gene expression were indistinguishable for both virus infections (S4A–S4D Fig).

### VOC Alpha and B.1 efficiently dampen induction of innate immunity in human bronchial airway epithelial cells (hBAECs)

**Fig 4:**
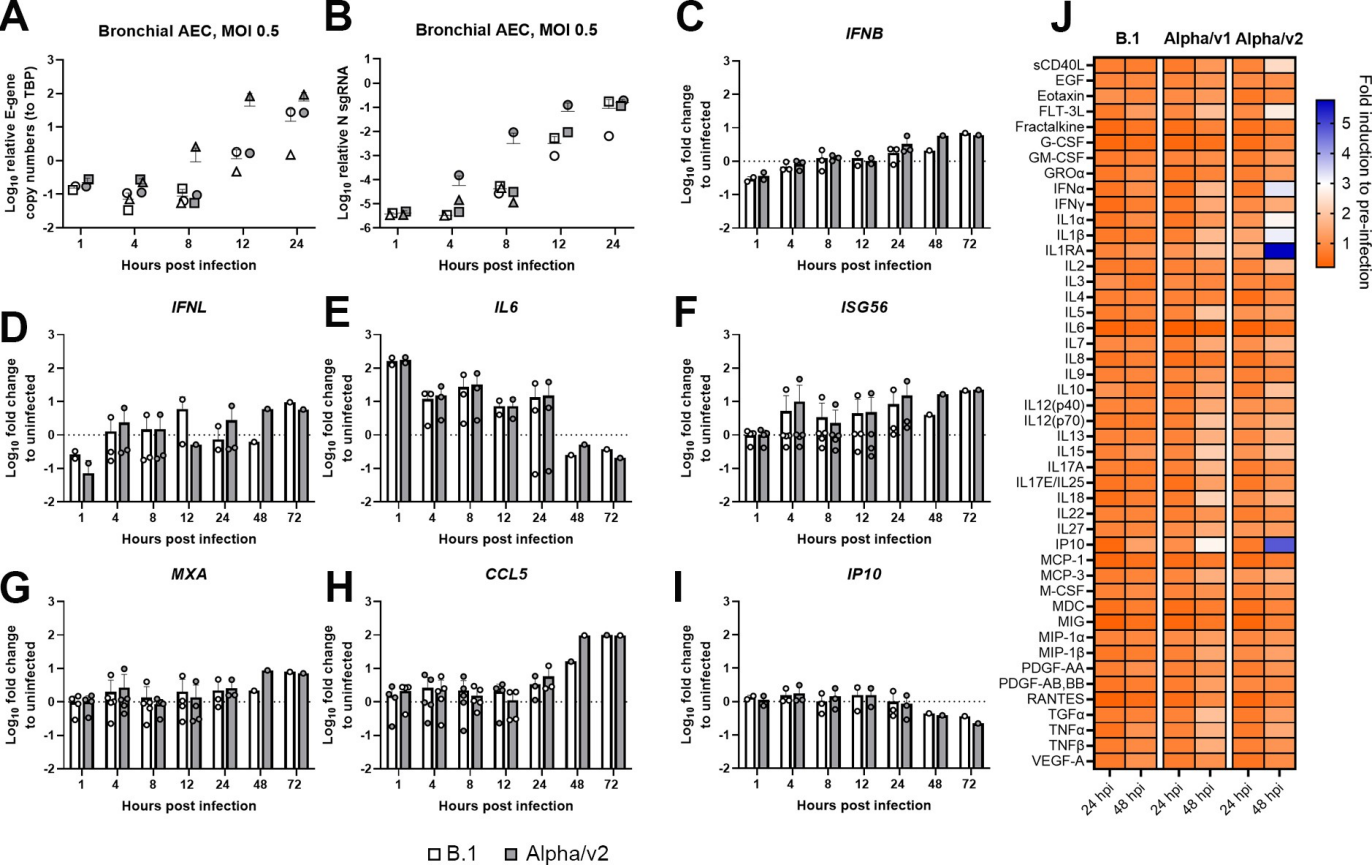
VOC Alpha and B.1 efficiently dampen induction of innate immunity in hBAECs. hBAECs were infected with B.1 or VOC Alpha (MOI of 0.5) and cell lysates were generated at the indicated time points followed by total RNA extraction. The experiment was performed with cells derived from 1–5 adult donors that were infected in duplicates. (**A**) Cell-associated expression of *envelope* in hBAECs during the early phase of infection determined by Q-RT-PCR. TBP was used for normalization. (**B**) Cell-associated expression of sgN in hBAECs during an early phase of infection determined by Q-RT-PCR. (**C**–**I**) Expression of the indicated genes was determined by Q-RT-PCR. Shown is the mean fold change +/− SD. (**J**) Relative change (to pre-infection) of cytokines and chemokines concentration in the basal medium of infected hBAECs (MOI 0.5). Concentration of cytokines and chemokines was determined by MagPix Luminex technology. Paired *t* tests were conducted between B.1 and VOC Alpha-infected groups and scored negative. AEC, airway epithelial cells; hBAEC, human bronchial airway epithelial cell; MOI, multiplicity of infection; Q-RT-PCR, quantitative real-time PCR; sgN, subgenomic nucleocapsid; TBP, TATA-binding protein; VOC, variant of concern. See S1 Data.

To detect potential variant-specific differences that may not become evident in immortalized Calu-3 and A549 cells, we studied innate immune gene expression in primary human bronchial airway epithelial cells (hBAECs) from different donors. For cells from most donors, we observed that quantities of genomic RNA (Fig 4A) and nucleocapsid transcription (Fig 4B) were similar between both variants in hBAECs. In 1 donor, genome replication and nucleocapsid transcription (Fig 4A and 4B) of VOC Alpha versus B.1 was slightly enhanced in the early phase of infection, revealing donor-specific differences of SARS-CoV-2 replication in primary hBAECs. Innate immune gene expression was efficiently and similarly suppressed in B.1 and VOC Alpha-infected hBAECs (Fig 4C–4I). For both virus infections, expression of *IFNB*, *IFNL*, *ISG56*, *MXA*, and *IP10* was merely less than 10-fold enhanced until 72 hours postinfection, while *CCL5* expression was up-regulated up to 100-fold as compared to uninfected cells at 48 and 72 hours postinfection. Of note, initial virus exposure induced *IL6* expression (100-fold), which was subsequently down-regulated to background levels at 48 and 72 hours postinfection. Also, in the basal medium of infected differentiated bronchial airway epithelial cell cultures, no significant variant-specific differences were identified for various cytokines and other secreted proteins related to innate immunity, including IFN-α, IFN-γ, and IP10 (Fig 4J). Overall, suppression of immune gene expression by VOC Alpha and B.1. did not differ in hBAECs.

### VOC Alpha spike protein shows decreased proteolytic processing

**Fig 5:**
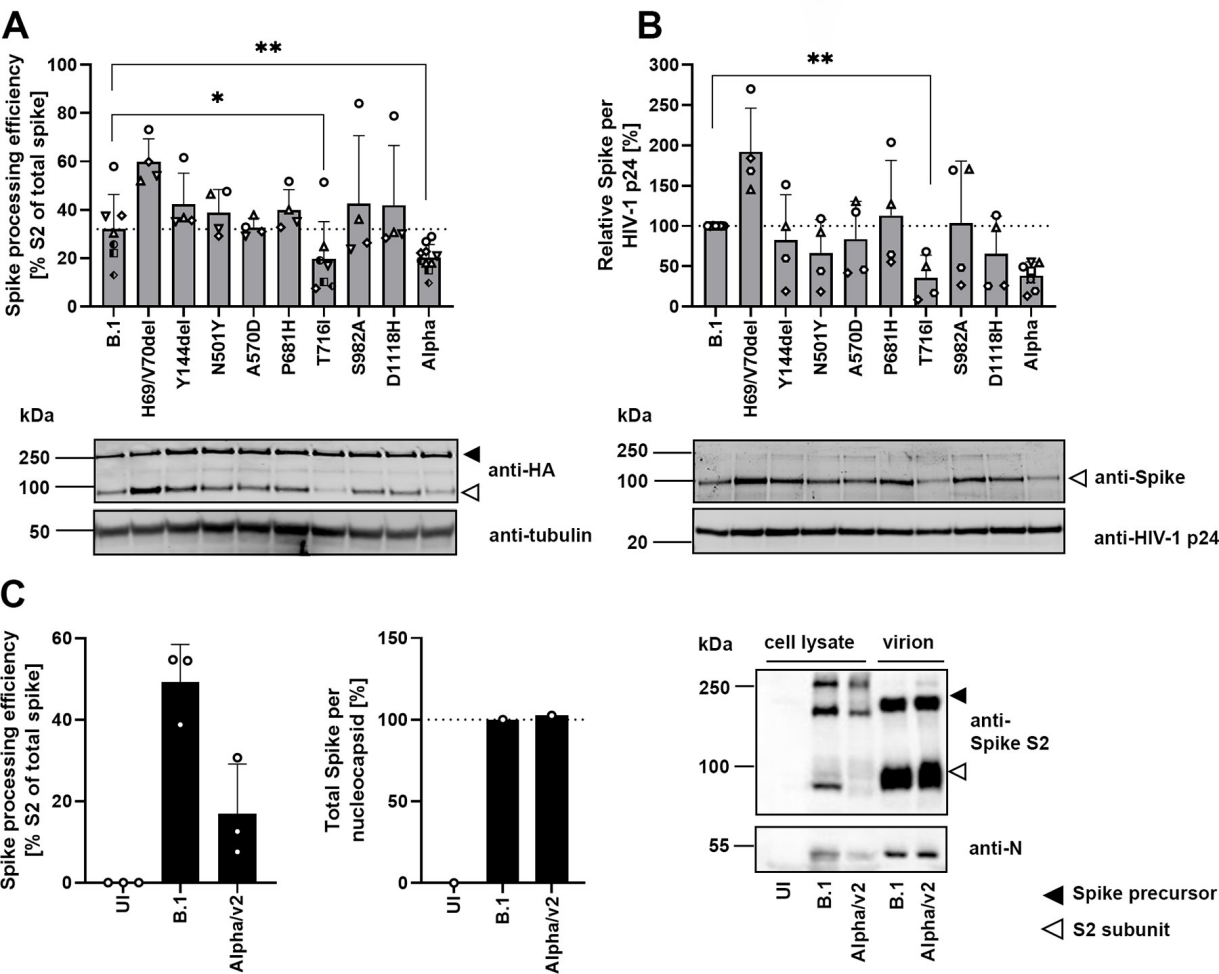
VOC Alpha spike protein shows decreased proteolytic processing. (**A**) Spike processing in lysates of HEK293T cells expressing empty vector or SARS-CoV-2 spike-HA encoding individual or all VOC Alpha-corresponding mutations was quantified by immunoblotting (upper panel). At least 4 independent biological replicates (using independent lentivirus particle preparations) were performed. Shown is 1 representative immunoblot (bottom panel) out of 4. (**B**) Protein in lysed lentiviral particles pseudotyped with SARS-CoV-2 spike-HA was quantified by immunoblotting (upper panel). Shown is 1 representative immunoblot (bottom panel) out of 4. (**C**) Vero E6 cells were infected with SARS-CoV-2 (MOI 5). Cells and virus-containing supernatants were harvested at 48 hours postinfection and processed for detection of spike and nucleocapsid by immunoblotting. Processing of spike in cell lysates (left panel) and spike incorporation in concentrated virion preparations (middle panel) was quantified. One representative blot out of 2 is shown (right panel). Black and white arrowheads indicate the bands of the uncleaved spike-HA precursor and of the cleaved S2-HA subunit, respectively. Statistical significance was calculated by a 2-tailed, paired Student *t* test. kDa, kilodalton; MOI, multiplicity of infection; SARS-CoV-2, Severe Acute Respiratory Syndrome Coronavirus 2; UI, uninfected; VOC, variant of concern. See S1 Data.

To identify potential consequences of VOC Alpha spike mutations on expression and proteolytic processing of the glycoprotein, we analyzed lysates of HEK293T cells transfected with plasmids expressing SARS-CoV-2 spike with an influenza A hemagglutinin gene fusion tag (-HA). Overall expression levels of spike constructs encoding individual or all VOC Alpha-specific mutations did not differ significantly from B.1 constructs in quantitative immunoblots (S5A Fig). However, quantification of the proportion of S2-HA spike relative to the total spike-HA signal revealed a 1.8-fold reduction of cleavage products of the VOC Alpha glycoprotein compared to that of B.1 (Fig 5A).

Deletion of H^69^/V^70^ showed a trend toward more efficient processing, in agreement with [34], and T^716^I showed a 1.6-fold decreased proteolytic processing as compared to B.1, suggesting that the latter mutation, located in the S1/S2 protease cleavage domain in proximity to the polybasic furin cleavage site, may render proteolytic processing of VOC Alpha spike less efficient. Reduced processing of VOC Alpha spike was accompanied by a 2.3-fold decrease of spike levels associated with lentiviral particles, when compared to particles containing B.1 spike (Fig 5B). Interestingly, the individual T^716^I mutation was sufficient for reduction of spike quantities associating to lentiviral particles. In accordance with previous reports [34,35], deletion of H^69^/V^70^ increased spike abundance in pseudotyped particles.

We next determined the efficiency of spike processing in the context of authentic SARS-CoV-2 infection. VOC Alpha spike in infected Vero E6 cells showed a 2.4-fold reduced abundance of cleavage product (Fig 5C, left panel and immunoblot, S5B Fig). In virus particles, the ratio of spike per nucleocapsid signal appeared intact, suggesting that inefficient proteolytic cleavage does not translate into a decreased association of mature S2 into virions (Fig 5C, middle panel and immunoblot) in contrast to lentiviral pseudotypes. Overall, expression levels of spike did not differ significantly from B.1 in quantitative immunoblots (S5C Fig).

### Enhanced cell–cell fusion and reduced virus particle entry by VOC Alpha SARS-CoV-2 spike

**Fig 6:**
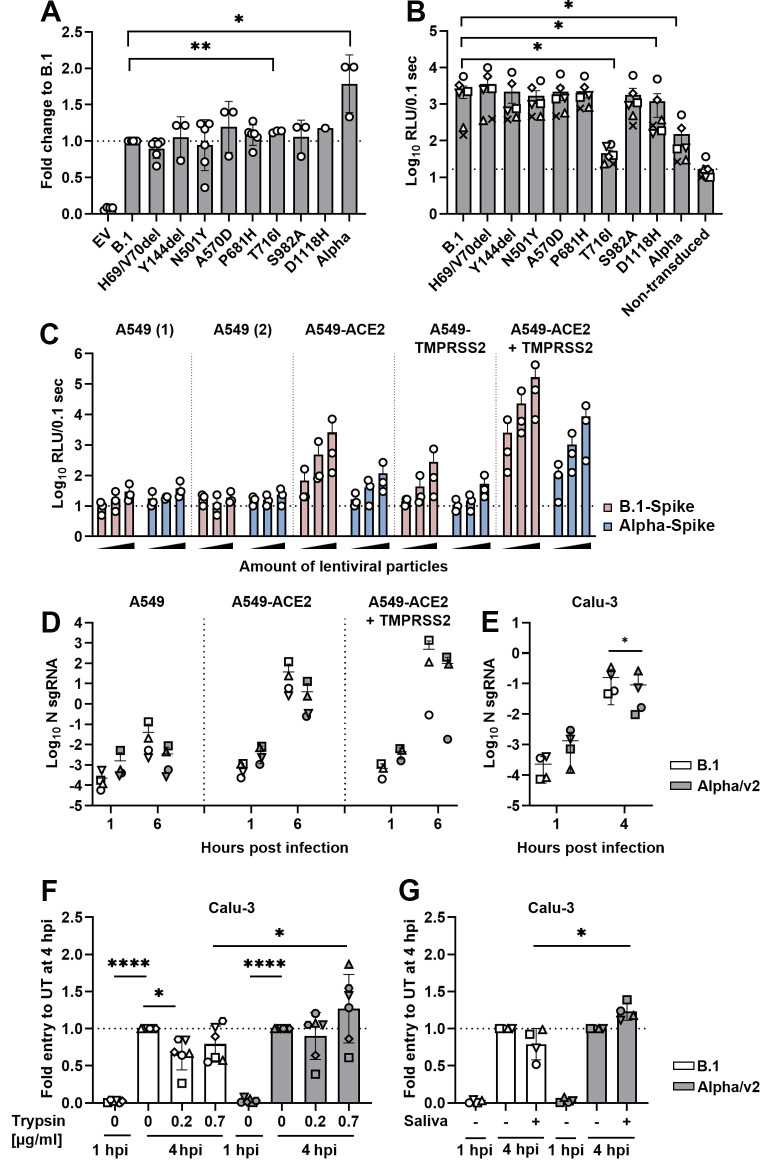
Enhanced cell–cell fusion and reduced virus particle entry by VOC Alpha SARS-CoV-2 spike. (**A**) For Tat-mediated cell–cell fusion assay, CHO cells were cotransfected with plasmids expressing indicated spike-HA and HIV-1 Tat. LTR-luciferase-expressing target TZM-bl cells were transfected with plasmids encoding human ACE2 and TMPRSS2. Transfected cells were cocultured for 8 hours and luciferase expression resulting from intercellular Tat transfer was quantified luminometrically. All values were normalized to B.1 spike (indicated by a dotted line). Shown are results from 3–6 biological replicates, each performed in triplicates. (**B**) Calu-3 cells were transduced with lentiviral pseudoparticles expressing luciferase and decorated with indicated spike-HA. Transduction efficiency was quantified luminometrically. Dotted line indicates background levels of luciferase nontransduced cultures. Shown are results from 6 independent biological replicates (using independent lentivirus particle preparations), each performed in triplicates, indicated by symbols. (**C**) Indicated A549 cells were transduced with increasing quantities (0.5 μl, 5 μl, and 50 μl) of lentiviral, luciferase-expressing particles pseudotyped with B.1- or VOC Alpha-spike. Transduction efficiency was determined luminometrically. Dotted line indicates luciferase background level of luciferase detected in nontransduced cells. Symbols represent individual values of 3 biological replicates, each performed in triplicates. (**D**, **E**) Indicated A549 (**D**) and Calu-3 (**E**) cells were infected at 4°C with B.1 or VOC Alpha isolates (MOI 1) to allow synchronized infection. Total cellular RNA was isolated at the indicated time points and nucleocapsid-encoding sgRNA was quantified by Q-RT-PCR. *N* = mean of 3–4 biological replicates, indicated by symbols. (**F**) Synchronized infection of Calu-3 cells was performed with B.1 and VOC Alpha virions that were pretreated with trypsin for 1 hour at 37°C. Data were normalized to the respective log_10_ relative N sgRNA level of the untreated (0 μg/ml) 4 hours postinfection sample (dotted line). Means +/− SD of 6 independently performed experiments are shown. (**G**) Synchronized infection of Calu-3 cells was performed with B.1 and VOC Alpha virions that were pretreated with saliva (pooled saliva from 3 healthy donors) for 1 hour at 37°C. Data were normalized to the respective log_10_ relative sgRNA N level of the untreated (-) 4 hours postinfection sample (dotted line). Results show means of 4 independently performed experiments, which were each performed in triplicates. Del, deletion; MOI, multiplicity of infection; Q-RT-PCR, quantitative real-time PCR; RLU, relative light units; SARS-CoV-2, Severe Acute Respiratory Syndrome Coronavirus 2; sgRNA N, subgenomic nucleocapsid RNA; TMPRSS2, transmembrane protease serine subtype 2; VOC, variant of concern. See S1 Data.

We next analyzed fusogenicity of individual spike proteins in a cell–cell fusion assay based on cocultures of CHO cells transiently expressing HIV-1 Tat and individual SARS-CoV-2 spike proteins, and ACE2/TMPRSS2-transfected, LTR-luciferase-expressing target TZM-bl cells. Compared to B.1 spike, no significant changes in membrane fusion activity were detected with any single mutation present in VOC Alpha spike (Fig 6A). However, full VOC Alpha spike was more prone to induce cell–cell fusion, corroborating a previous study [36]. Furthermore, entry of lentiviral pseudotypes mediated by the same set of spike proteins was quantified after transduction of Calu-3 cells using p24 capsid normalized inocula in a luciferase-based assay (Fig 6B). Whereas most individual mutations did not significantly alter the ability of SARS-CoV-2 spike to mediate entry into Calu-3 cells, T^716^I and the full VOC Alpha spike mediated reduced entry (Figs 6B and S6A). The inferior ability of VOC Alpha spike-containing lentiviral pseudotypes to transduce Calu-3 cells was corroborated in titration experiments on ACE2/TMPRSS2-expressing A549 cells (Fig 6C) and is potentially related to the lower levels of incorporated spike when compared to B.1 spike-pseudotyped particles.

We next investigated entry kinetics in ACE2/TMPRSS2-expressing A549 and Calu-3 cells with authentic SARS-CoV-2. In single-cycle entry experiments, virus inocula were absorbed at 4°C for 1 hour, cells were washed to remove excessive virus particles, and eventually incubated at 37°C to initiate synchronized entry. De novo-synthesized, cell-associated subgenomic transcripts served for detection of early signs of virus replication after entry (Fig 6D and 6E). Reminiscent of our results obtained with the lentiviral pseudotypes, VOC Alpha showed slightly lower levels of viral transcription in Calu-3, ACE2-, and ACE2/TMPRSS2-expressing A549 cells when compared to B.1. ACE2 was essential for virus entry into A549 cells, as levels of subgenomic viral RNA remained at background levels in parental A549. Entry of both viruses in Calu-3 cells depended on TMPRSS2 and furin and less on the cathepsin L-dependent pathway as judged by treatment with specific inhibitors. In addition, clathrin inhibition resulted in decreased entry of B.1 and VOC Alpha in Calu-3 cells (S6B Fig), as previously described for SARS-CoV [37]. These findings were confirmed with Calu-3-derived virus stocks to exclude the possibility that Vero E6-specific properties might alter proteolytic processing of spike (S6C Fig).

As tissue-specific salivary proteases are present in the extracellular milieu of the respiratory tract [38], we determined entry efficiency of SARS-CoV-2 virions into Calu-3 cells upon treatment of virions with exogenous trypsin prior to infection. While entry of B.1 was significantly reduced, VOC Alpha entry was largely unaffected by trypsin treatment with a slight trend toward increased entry upon treatment with 0.7 μg/ml of trypsin and statistically significant enhancement of entry efficiency for VOC Alpha as compared to B.1 (Fig 6F). To investigate how salivary proteases contribute to SARS-CoV-2 entry, we determined entry efficiency of SARS-CoV-2 into Calu-3 cells upon pretreatment of virions with pools of saliva from 3 healthy donors (without recent infection and/or vaccination to exclude the possibility of mucosal anti-spike antibodies) (Fig 6G and S1 Table). While entry of B.1 was slightly impaired by exposure of particles to saliva, VOC Alpha virions were resistant and even showed a slight trend toward increased entry. A direct comparison of entry efficiency of saliva-treated virions revealed a statistically significant advantage for Alpha.

In conclusion, VOC Alpha spike-pseudotyped lentiviral particles and authentic VOC Alpha virions were less efficient in entering susceptible target cells than their B.1 counterparts. This inefficiency may be related to reduced abundance of VOC Alpha spike cleavage product, which may translate in an enhanced resistance of spike to trypsin and salivary protease-mediated premature activation.

### VOC Alpha and Delta display a postentry, spike-dependent replication advantage in NCI-H1299 cells, a human bronchial cell line with undetectable ACE2 protein level

**Fig 7:**
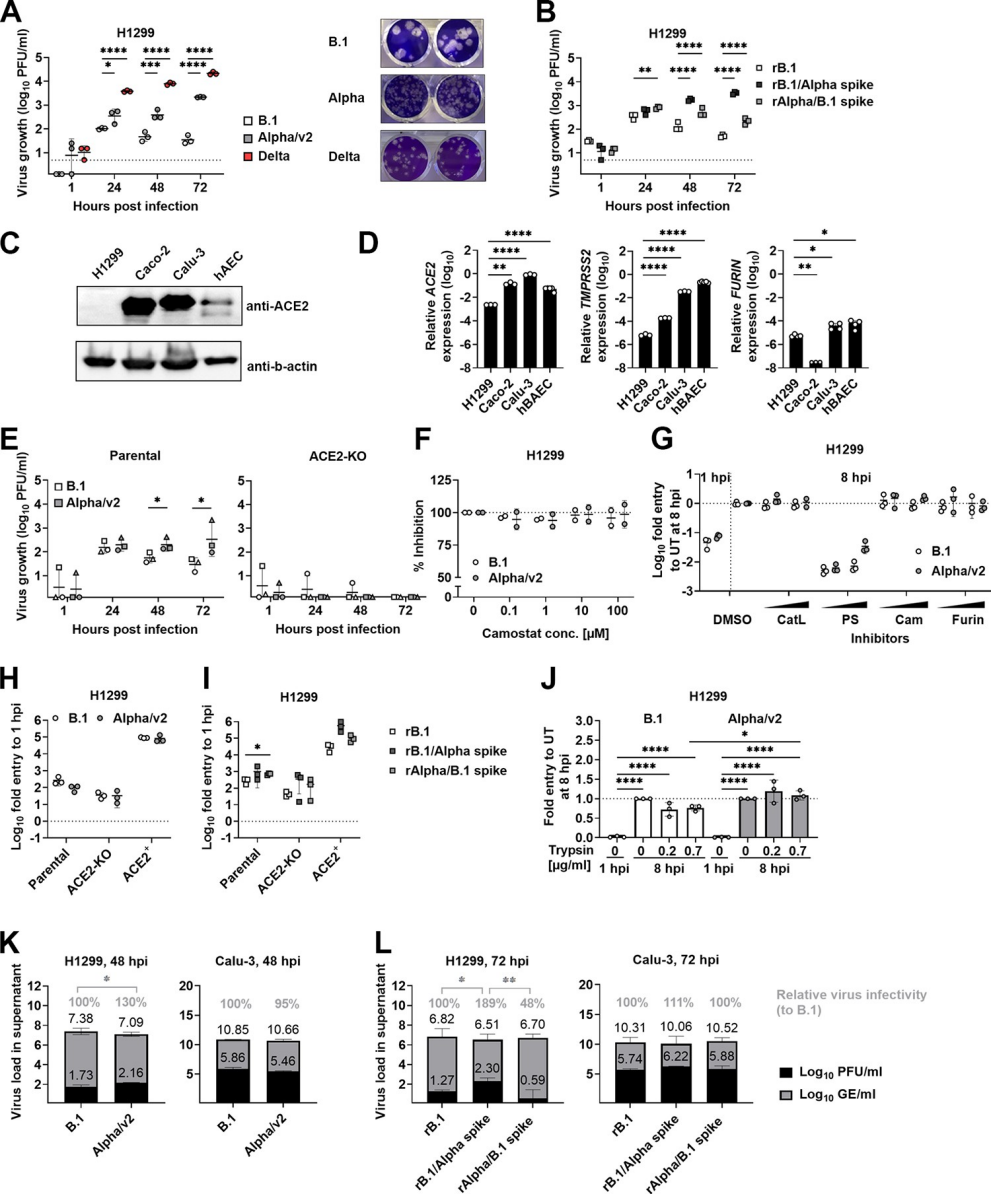
VOC Alpha and Delta display a postentry, spike-dependent replication advantage in NCI-H1299 cells, a human bronchial cell line with undetectable ACE2 protein level. (**A**) Virus growth of B.1, VOC Alpha, and Delta was assessed on NCI-H1299 cells. Cells were infected (MOI 0.01) and supernatants of indicated time points were titrated on Vero E6 cells (left panel). Plaque morphology of NCI-H1299-derived B.1, VOC Alpha, and Delta on Vero E6 cells is shown (right panel). Results from 1 representative experiment out of 3 is shown. (**B**) NCI-H1299 cells were infected with rB.1, rB.1/Alpha spike, or rAlpha/B.1 spike viruses (MOI 0.01) and supernatant was titrated on Vero E6 cells. The growth experiment was performed once in triplicates. (**C**) ACE2 expression levels were analyzed by immunoblotting. Beta-actin was used as a loading control. (**D**) Expression of *ACE2*, *TMPRSS2*, and *FURIN* was quantified by Q-RT-PCR in indicated cells. (**E**) Virus growth of B.1 and VOC Alpha was investigated on parental-NCI-H1299 (left) and respective ACE2-KO (right) cells. Cells were infected (MOI 0.01) and supernatants of indicated time points were titrated on Vero E6 cells. Three independent experiments, each in triplicates, were performed and are indicated by symbols. (**F**) NCI-H1299 cells were treated with increasing amounts of camostat mesylate (0–100 μM) for 2 hours at 37°C prior to infection with B.1 and VOC Alpha (MOI 0.01). After infection for 1 hour at 37°C, camostat mesylate was replenished to the infection medium and cells were incubated for 48 hours. Virus replication was determined from the supernatant by E gene assay. All values were normalized to the respective B.1 or VOC Alpha-infected, untreated control cells (dotted line at 100%). The experiment was performed once in duplicates. (**G**) NCI-H1299 cells were pretreated for 2 hours at 37°C prior to infection with B.1 and VOC Alpha (MOI 2) with MDL28170 (Cathepsin L inhibitor, 12.5 and 25 mM), pitstop II (clathrin inhibitor, 12.5 and 25 μM), camostat mesylate (TMPRSS2 inhibitor, 50 and 100 μM), or CMK (furin inhibitor, 2.5 and 5 μM). Entry efficiency was determined at 8 hours postinfection from cell lysates by sgN Q-RT-PCR. The experiment was performed once in triplicates. (**H**, **I**) NCI-H1299, ACE2-KO-H1299, and ACE2^+^-H1299 cells were infected in triplicates at 4°C with (**H**) B.1 and VOC Alpha isolates or (**I**) rB.1, rB.1/Alpha spike, and rAlpha/B.1 spike viruses (MOI 2) to allow synchronized entry. Relative quantities of cell-associated nucleocapsid-specific subgenomic RNA were determined by Q-RT-PCR. Three independent experiments were performed, each conducted in 4 replicates. Symbols represent the arithmetic means of each experiment. (**J**) Synchronized infection of NCI-H1299 cells was performed with B.1 and VOC Alpha virions that were pretreated with trypsin for 1 hour at 37°C. Data were normalized to the respective log_10_ relative sgRNA N transcription of the untreated (UT) 8 hours postinfection sample (dotted line). Results of 3 independently conducted experiments, which were each performed in triplicates, are shown. (**K**) NCI-H1299 and Calu-3 cells were infected with B.1 and VOC Alpha isolates (MOI 0.01) for 48 hours and supernatant was titrated by plaque assay on Vero E6 cells to determine PFU/ml. Genome equivalents (GE/ml) were determined by E gene assay. Two independent experiments, each in triplicates, were performed. Gray numbers indicate mean relative virus infectivity of VOC Alpha to B.1 of the independent experiments. (**L**) NCI-H1299 and Calu-3 cells were infected with rB.1, rB.1/Alpha spike, and rAlpha/B.1 spike viruses (MOI 0.01) for 72 hours and supernatant was titrated by plaque assay on Vero E6 cells to determine PFU/ml. Genome equivalents (GE/ml) were determined by E gene assay. Two independent experiments, each in triplicates, were performed. Gray numbers indicate mean relative virus infectivity to rB.1 of the independent experiments. Bars represent arithmetic means of independent experiments. KO, knock-out; MOI, multiplicity of infection; PFU, plaque-forming units; Q-RT-PCR, quantitative real-time PCR; VOC, variant of concern. See S1 Data.

Upon further exploration of susceptible cell lines, we identified that the human bronchial cell line NCI-H1299 yields a 24-fold higher replication level for VOC Alpha compared to B.1 in multicycle growth kinetics (Fig 7A). NCI-H1299 cells have an epithelial-like phenotype, were isolated from a lung-associated lymph node of a patient with a non-small cell lung carcinoma, and were reported to show largely intact cellular innate immunity functions [39,40]. Remarkably, VOC Delta grew to 264-fold (range, 37- to 595-fold) higher titers than B.1 and 24-fold (range, 3- to 59-fold) higher titers than VOC Alpha (Fig 7A) in this cell line, whereas Calu-3 cells and hBAEC cultures yielded similar or only slightly increased titers of Delta (S7A and S7B Fig). To analyze to which extent the NCI-H1299 cell-specific enhancement of VOC Alpha replication is determined by spike, we generated a recombinant B.1 virus (rB.1), a B.1 virus carrying the full spike protein of VOC Alpha (rB.1/Alpha spike) and a virus consisting of the VOC Alpha backbone but the B.1 spike (rAlpha/B.1 spike; Figs 7B, S7C and S7D). All recombinant viruses grew to similar titers in Vero E6 cells (S7C Fig). In Calu-3 cells, growth of rAlpha/B.1 spike was increased 9-fold over rB.1 at 72 hours postinfection, suggesting that the VOC Alpha backbone confers some intrinsic replication advantage that does not depend on the spike protein (S7D Fig). In NCI-H1299 cells, the virus expressing the VOC Alpha spike in the backbone of B.1 grew to considerably higher titers than the reciprocal virus and the original rB.1 virus (up to 65-fold and 15-fold increased titer, respectively; Fig 7B), indicating that the replicative advantage of VOC Alpha in NCI-H1299 cells is determined by the spike protein. Surprisingly, replication of VOC Alpha in NCI-H1299 cells occurred despite ACE2 being undetectable by immunoblotting (Figs 7C and S8A) and flow cytometry (S8B Fig). Also, *ACE2*, *TMPRSS2*, and *FURIN* mRNAs were only weakly expressed as compared to other SARS-CoV-2-susceptible cell cultures (Fig 7D). To elucidate the role of potentially minute amounts of ACE2 in entry into NCI-H1299 cells, we blocked ACE2 by antibodies. In Vero E6 and Calu-3 cells, incubation with any of 3 ACE2-neutralizing antibodies abolished and diminished SARS-CoV-2 infection (S8C Fig, left and middle panel). In NCI-H1299 cells, anti-ACE2 antibody treatment did not or only very modestly modulate susceptibility (S8C Fig, right panel, and S8D Fig, right panel), raising the question whether ACE2 might be dispensable for entry of SARS-CoV-2 in NCI-H1299.

Next, we *knocked-out ACE2* in NCI-H1299 cells (ACE2-KO-H1299) by the use of CRISPR/Cas9 technology (S8E Fig). Here, productive infection by B.1 and VOC Alpha was completely abolished by the *ACE2 knock-out* in a multicycle assay, reflected by absence of both secreted genomic RNA (S8F Fig) and infectivity (Fig 7E), and reconstitution of *knock-out* cells with ACE2 expression in *trans*, as opposed to an empty vector, rescued infection (S8G Fig). As deduced from experiments with TMPRSS2- (camostat mesylate) and clathrin (pitstop II)-specific inhibitors, entry in NCI-H1299 cells was TMPRSS2 independent and clathrin mediated (Figs 7F and 7G and S8H). However, in contrast to these assays measuring virus spread over several days, *ACE2 knock-out* did not prevent entry of B.1 and VOC Alpha virus isolates as well as chimeric SARS-CoV-2, as judged from synchronized entry assays, which revealed initial nucleocapsid-specific sgRNA transcription (Fig 7H and 7I). Together, a low basal, though similar level of de novo synthesized nucleocapsid-specific sgRNA in ACE2-KO-H1299 and parental cells upon infection with SARS-CoV-2 isolates and chimeric spike viruses suggests that NCI-H1299 cells provide other receptors than ACE2, whose usage nevertheless results in an abortive infection. Along this line, there was no advantage in entry of lentiviral particles based on the VOC Alpha spike protein in NCI-H1299 cells (S8I Fig), arguing for a postentry, spike-specific advantage of VOC Alpha in these cells. However, ACE2 was essential for a productive, spreading SARS-CoV-2 infection of NCI-H1299 cells. Of note, low entry and replication levels can be increased up to 340-fold (B.1) and 883-fold (VOC Alpha) or 22-fold (B.1 at 24 hours postinfection) and 43-fold (VOC Alpha at 72 hours postinfection) through heterologous expression of ACE2 in a stable and transient fashion, respectively (Figs 7H, 7I, S8A, S8B and S8G).

Similar to the situation in Calu-3 cells (Fig 6F), also in NCI-H1299 cells VOC Alpha was more resistant to trypsin pretreatment (Fig 7J). Finally, we reasoned that the growth advantage of VOC Alpha in NCI-H1299 cells, which manifested itself particularly at later time points of infection (48 and 72 hours postinfection; Fig 7A), could be related to enhanced stability of infectivity of released VOC Alpha virions and conducted an analysis of specific particle infectivity by calculating the ratio of infectivity per genomic RNA abundance in released virions (Figs 7K, 7L and S9). While virions released from B.1- and VOC Alpha-infected NCI-H1299 cells displayed stable levels of viral genomic RNA from 24 to 72 hours postinfection, infectivity of B.1 virions gradually decreased (from 2.29 to 1.39 log_10_ PFU/ml), as opposed to VOC Alpha infectivity (from 2.15 to 2.13 log_10_ PFU/ml) (S9A Fig). This VOC Alpha-specific prolonged and increased stability of infectivity was specifically detected in the context of NCI-H1299, but not Calu-3 cell infection (S9B Fig). For example, at 48 hours, stability of VOC Alpha versus B.1 particle infectivity was enhanced by 30% in NCI-H1299 and reduced by 5% in Calu-3 (Figs 7K, S9A and S9B). This observation was corroborated in the context of recombinant chimeric viruses, with prolonged stability of particle infectivity specifically observed for rB.1/Alpha spike, and not for rB.1 and rAlpha/B.1 spike (Figs 7L, S9C and S9D), reemphasizing the important contribution of spike and specifically occurring in NCI-H1299, but not Calu-3 cells. In addition, RNA replication, abundance of *nucleocapsid*-encoding sgRNAs, as well as expression of selected innate immune genes and pro-inflammatory cytokine genes were undistinguishable between B.1- and VOC Alpha in NCI-H1299 cells (S10A–S10C Fig).

In sum, the identified growth advantage of VOC Alpha in NCI-H1299 cells is related to enhanced VOC Alpha spike-specific stability of the infectious nature of particles, which might be facilitated by its lower abundance of processed spike. Together, prolonged infectious stability of particles may be specifically beneficial for infection of epithelial cells with low ACE2 expression levels.

## Discussion

To date, our understanding of viral and cellular determinants that underlie the rapid spread and/or increased pathogenicity of SARS-CoV-2 VOC Alpha is limited. As SARS-CoV-2 productively infects epithelial cells [41], and VOC Alpha-infected patients shed 10-fold more viral RNA [10], we initially expected that VOC Alpha has a replication advantage in human epithelial cell cultures. Although all immortalized, primary, and organoid cultures tested were highly susceptible to and permissive for SARS-CoV-2 infection, we failed to detect a VOC Alpha-specific growth advantage in almost all of these models. Our findings corroborate reports by others showing similar growth of VOC Alpha and B.1. viruses in common culture cells and primary human airway epithelial (HAE) cells [25,26,28]. Despite monitoring infected cultures for up to 10 days, we failed to observe temporally increased virus production in human organoid models as detected by others [42]. In vivo, VOC Alpha showed a fitness advantage in experimentally infected ferrets, in hACE2-K18Tg-transgenic mice, and in hACE2-KI-transgenic mice [25], but not in Syrian hamsters [25,27]. Complementing these reports, our data confirm the absence of significant growth differences between VOC Alpha and B.1 variants in experimentally infected dwarf hamsters [43]. However, a single study observed a transmission advantage of VOC Alpha particularly upon low dose infection of Syrian hamsters [44]. Finally, in intranasally infected African green monkeys, VOC Alpha infection generated higher levels of viral RNA in the respiratory tract than B.1 infection [29]. Together, the increased fitness of VOC Alpha can be recapitulated in some animal models but is not detectable in most cell culture systems.

Whereas increased VOC Alpha replication has been observed in patients, the initial phase of virus infection is difficult to capture in clinical observations due to late sampling, making cell culture studies potentially more insightful. On the contrary, cell cultures may fail to reflect differences in virus production in later stages of tissue infection due to the limiting effect of cytopathogenic effects in vitro. Reduced growth of VOC Alpha in cell culture does not necessarily contradict clinical observations. We observed a slower ramp-up of virus production and a delayed onset of cytopathic effect. Accordingly, infected air–liquid interface cultures of alveolar type 2 cells produced more infectious VOC Alpha virus when compared to B.1 only in a late phase of infection [42]. Also, replication of B.1 and VOC Alpha variants in the upper respiratory tract of experimentally infected African green monkeys differed only from day 5 postinfection onward [29]. Together, this suggests a delayed but extended phase of infectious VOC Alpha virion shedding, potentially resulting in a prolonged phase of heightened transmission probability, which is also supported by viral load studies [10,45].

B.1 SARS-CoV-2 productively infects susceptible cells via binding of the spike protein to ACE2 and TMPRSS2-mediated priming of spike [46]. Compared to B.1 viruses, VOC Alpha viruses and VOC Alpha spike-decorated lentiviral particles were equally or even less efficient in entering ACE2-expressing cell lines. Intriguingly, VOC Alpha spike appeared to be more fusogenic in cell–cell fusion assays, in accordance with published work [34,47]. This phenotypic property has been ascribed to changes in pathogenicity, e.g., in VOCs Delta and Omicron [20,48]. An increase of pathogenicity has been proposed on the basis of epidemiological data also for VOC Alpha [49]. Proteolytic processing and viral packaging of coronaviral spike can be rate limiting during infectious virus production and might be influenced by species-specific expression of cofactors and restriction factors. Our findings of reduced proteolytic cleavage in spike plasmid-transfected HEK293T cells and SARS-CoV-2-infected VeroE6 and Calu-3 cells, however, argue against a major species-specific difference of this process and are consistent with reduced furin-mediated processing of VOC Alpha spike as shown by a biochemical peptide cleavage assay [50], and challenge data reporting intact processing of cell-associated spike when expressed in spike plasmid-transfected cells [20]. Of note, the latter study used plasmids expressing spikes lacking 19 carboxyl-terminal amino acids, an experimental modification that has been widely accepted for artificial enhancement of cell surface expression and lentiviral incorporation of spike [51] and that we refrained to adopt in order to maintain the expression context as physiological as possible. Moreover, the reduced abundance of processed cleavage products of VOC Alpha mapped to the spike mutation T^716^I, which is located in close proximity to the S1/S2 cleavage site. It is conceivable that T^716^I either sterically hinders proteolytic spike processing or reduces exposure of the nearby furin cleavage motif. T^716^I mutation is a uniquely VOC Alpha-defining mutation (Nextstrain analysis conducted in August 2022), suggesting that the T^716^I-related spike stabilization strategy is unique to VOC Alpha.

The observation of reduced processing translated into lower levels of lentivirus-associated spike, which may be the cause of the reduced infectivity of particles. Even though impaired maturation did not detectably alter virion-associated spike levels under our experimental conditions, we cannot exclude that it may still modulate the kinetics of virus particle secretion and/or the quality of secreted SARS-CoV-2 particles.

Paradoxically, ACE2 expression levels in the respiratory tract are low and common cell culture models may not fully recapitulate in vivo properties of primary target tissues of SARS-CoV-2 infection [52]. Our systematic exploration of multiple cell cultures uncovered a cell line that has remained understudied with regard to SARS-CoV-2 infection phenotypes. While caveats and limitations of neoplastic cell lines apply, NCI-H1299 cells yielded higher levels of replication and infectious virus production not only for VOC Alpha, but also for VOC Delta. This cell line of epithelial morphology is devoid of detectable ACE2 protein and remains susceptible to SARS-CoV-2 infection. Along this line, early production of de novo synthesized nucleocapsid-specific sgRNA in ACE2-KO-H1299 and parental cells postinfection with isolates and chimeric viruses met our initial assumption that NCI-H1299 cells may possess alternative SARS-CoV-2 receptors. This finding is reminiscent of a recent study [53] that identified SARS-CoV-2 infection of the NCI-H522 lung cell line in an ACE2-independent fashion (in this study, the alternative way of infection could only be utilized by viruses carrying an E^484^D substitution within the spike protein RBD). However, although ACE2 protein is undetectable in NCI-H1299 cells, genetic *knock-out* of ACE2 resulted in the loss of virus growth in NCI-H1299 cells, which underlines the essential nature of ACE2 for productive and spreading infection in NCI-H1299 cells. Regarding TMPRSS2 dependence, we detected only minor expression level and comparison of entry efficiencies in the presence of a TMPRSS2 entry inhibitor demonstrated a preference for clathrin-mediated endosomal entry in B.1 and VOC Alpha-infected NCI-H1299 cells, an entry route that is more frequently utilized by closely related sarbecoviruses with monobasic furin cleavage sites.

Surprisingly, while experiments with reciprocal chimeras established that the VOC Alpha growth superiority in NCI-H1299 cells is mediated to a large extent by its spike, the mere entry process per se was similarly or even less efficient for VOC Alpha than for B.1. These findings suggest a postentry, yet largely spike-dependent replication advantage of VOC Alpha that manifests itself in NCI-H1299 cells. We assume that this VOC Alpha-specific advantage may be due to enhanced stability of virion infectivity in the supernatant of infected NCI-H1299 cells. Compared to VOC Alpha, particle infectivity significantly decreased in B.1-infected NCI-H1299 cells over 72 hours, whereas it remained stable in Calu-3 cells. We hypothesize that reduced VOC Alpha spike proteolytic processing endows virions with stable particle infectivity in the extracellular milieu. The respiratory mucosa is a protease-rich environment [38] that could contribute to antiviral defense by proteolytic spike inactivation, rendering spike proteins nonfunctional for ACE2-dependent endosomal fusion after some time, including in emitted droplets and aerosols. In an attempt to mimic the respiratory mucosal environment by pretreating virions with exogenous trypsin and saliva, we show that B.1 virions lose their infectivity more rapidly than VOC Alpha virions under proteolytic conditions. Of note, the sensitivity of B.1 to trypsin-mediated inactivation has already been described [41]. The improved protection against proteolytic premature activation of VOC Alpha spike may be particularly beneficial for infection of cells expressing low ACE2 and TMPRSS2 levels, as represented by NCI-H1299 cells. With low ACE2 levels, a limited number of ACE2 molecules is available for productive endosomal infection by intact virions. This small number of ACE2 molecules may be more efficiently utilized by VOC Alpha due to its higher infection stability, whereas for B.1, a higher number of defective particles during the later phase of infection increases the likelihood that ACE2 molecules will be wasted for an abortive, nonproductive infection. These effects are likely to be rather neglectable when infecting cells with high ACE2 levels with or without TMPRSS2 as represented by Calu-3 and A549-reconstituted cells. However, since, paradoxically, only a minority of cells of the respiratory tract expresses *ACE2*, and dual ACE2/TMPRSS2-positive cells are even more rare [41,52,54] and extracellular proteases are present in high density in the mucosa [38], VOC Alpha may have adapted to these antiviral conditions by decreasing spike processing efficiency.

Remarkably, VOC Delta also displayed a similar level of replication in common ACE2-positive cell lines as compared to B.1 but a specific increase of replication in NCI-H1299 cells. In addition, VOC Delta showed a growth advantage in human BAECs that manifested itself not prior to 4 days post-infection. The extent of growth advantage for VOC Delta in NCI-H1299 cells was higher than for VOC Alpha, which is in line with the observed differences in epidemic growth rates for these VOCs. As other studies demonstrated improved processivity for the VOC Delta spike [17,55,56], convergent adaptation between VOCs Alpha and Delta spike can be excluded. For VOC Omicron sublineage BA.1, however, a decreased proteolytic processing of spike relative to VOC Delta was seen, the extent of which (although a direct comparison is lacking) might be similar to that of VOC Alpha observed in the present study. Decreased proteolytic processability was associated with a switch in BA.1’s entry route preference that changed from TMPRSS2-dependent cell surface fusion to ACE2-mediated endosomal entry [57]. VOCs Alpha and Omicron BA.1, rather than Delta, may have undergone convergent adaptations to the human upper respiratory tract with low ACE2 and TMPRSS2 levels.

Together, we propose that the stability of the spike in the extracellular milieu can be different for SARS-CoV-2 variants, which mainly affects the efficiency of infection of cells with low ACE2 levels. In this context, we identified a human bronchial cell line, NCI-H1299 cells, with undetectable ACE2 protein expression levels to be used as a model cell line for extended SARS-CoV-2 infection stability studies in the future. Further research on cell line-specific proteases is required to enhance our understanding on antiviral defense mechanisms of the extracellular environment of the respiratory mucosa.

## Materials and methods

### Samples from COVID-19 patients, vaccinees, and saliva samples

Sera were available through a study on convalescent plasma donors, who recovered from mild to moderate COVID-19 before the emergence of any SARS-CoV-2 VOCs [58]. Additional sera were available through a study on SARS-CoV-2 infection and COVID-19 vaccination [59], a prospective observational cohort study Pa-COVID-19 [60] including its study arm RECAST (Understanding the increased resilience of children compared to adults in SARS-CoV-2 infection) and from RT-PCR confirmed VOC Alpha-infected patients. The use of clinical samples (sera) was approved by the Institutional Review Board at Charité - Universitätsmedizin Berlin (EA1/068/20, EA2/092/20, and EA2/066/20) and is in accordance with the Berlin State Hospital Law, allowing for pseudonymized scientific analysis of routine patient data.

Saliva samples were taken from healthy donors whose COVID-19 vaccination and/or infection was at least 6 months ago to avoid spike-neutralizing mucosal immunity. Briefly, for sample generation, oral mucosa was gently scraped (to stimulate saliva production) with a swab before a 500 μl saliva sample was given. Saliva was then resuspended in 500 μl OptiPro serum-free medium containing 1% Penicillin-Streptomycin (Thermo Fisher Scientific) and 1% Amphotericin B (Thermo Fisher Scientific). The use of clinical saliva samples was approved by the Institutional Review Board at Charité - Universitätsmedizin Berlin (Convalescents; EA2/092/20, COVIMMUN; EA1/068/20, EICOV; EA4/245/20 and Pa-COVID-19; EA2/066/20).

### Cells and culture conditions

A549 parental (ATCC CCL-185), ACE2^+^-A549, TMPRSS2^+^-A549, ACE2^+^/TMPRSS2^+^-A549 [61], Caco-2 (ATCC HTB-37), Calu-3 (HTB-55), CHO (HIV Reagent Program ARP-2238), NCI-H1299 (ATCC CRL-5803), ACE2-KO-NCI-H1299, ACE2^+^-NCI-H1299, HEK 293T (ATCC CRL-3216), TZM-bl (HIV Reagent Program ARP-8129), and Vero E6 (ATCC CRL-1586) cells were maintained at 37°C and 5% CO_2_ in a humidified atmosphere and cultured in Dulbecco’s Modified Eagle’s Medium (DMEM, ThermoFisher Scientific) supplemented with 10% fetal bovine serum (FBS, Thermo Fisher Scientific), 1% nonessential amino acids 100× concentrate (NEAA, Thermo Fisher Scientific), and 1% sodium pyruvate 100 mM (NaP, Thermo Fisher Scientific) and split twice a week. For seeding and cultivation, cells were washed with phosphate buffered saline (PBS, Thermo Fisher Scientific) and detached with 0.05% trypsin-EDTA solution (Thermo Fisher Scientific).

### Generation of NCI-H1299 with ACE2 knock-out and ACE2-overexpressing cells

*ACE2*-targeting sgRNAs (CGGCCAGTTGATTGAAGATG) were cloned into plentiCRISPRv2 (Addgene #52961) using Esp3I restriction sites as described earlier [62]. Lentiviral particles were produced in HEK293T cells using FuGene6 (Promega) according to the manufacturer’s suggestions. Briefly, plentiCRISPRv2_ACE2 and packaging plasmids pMD2.G and psPAX2 (Addgene plasmids #12259 and #12260) were cotransfected in a 4:3:1 ratio into HEK293T cells at 60% confluency in 10 cm cell culture dishes. Medium was exchanged the next morning. Lentiviral particles were harvested 24 hours later, centrifuged at 300 x *g* at 4°C for 10 minutes to pellet cell debris. The supernatant was filtered through a 0.45-μm low protein binding membrane and used to transduce parental NCI-H1299 cells in presence of 8 μg/ml polybrene. After puromycin selection, single-cell clones were generated by limiting dilution. Sanger sequencing was used to confirm genomic modification of the target site (Fwd: 5’-TCCCAGCAAGGCTAATCTATGT-3’, Rev: 5’-GAATGCCTTTAGTCACTGTCCC-3’). For overexpression of human *ACE2* in NCI-H1299 parental and *ACE2 knock-out* cells, retroviral particles were generated using plasmid pQCXIP_hACE2 (pQCXIP from Clontech) encoding human ACE2, and the packaging plasmid pCMVi MLV gag pol [63] and pMD2.G. Overexpression of *ACE2* in pool populations was determined by immunoblotting and flow cytometry.

### Virus strains

Infection experiments were conducted with BetaCoV/Munich/ChVir984/2020 (B.1, EPI_ISL_406862), hCoV-19/Germany/BY-ChVir21652/2020 (VOC Alpha/v1, EPI_ISL_802995), BetaCoV/Baden-Wuerttemberg/ChVir21528/2021 (VOC Alpha/v2, EPI_ISL_754174), and hCoV-19/Germany/BW-ChVir22131/2021 (B.1.351, EPI_ISL_862149). A virus of the B.1.617.2 (“Delta”) clade (hCoV-19/Germany/SH-ChVir25702_4/2021) was isolated from a patient in Schleswig-Holstein, Germany, and its sequence deposited in Gisaid (EPI_ISL_2500366). Due to the observation of rapid cell culture-induced mutations at the spike polybasic furin cleavage motif (spike amino acid no. 681–685), all virus stocks were sequenced by next-generation sequencing to confirm the absence of minority variants. Unless otherwise stated, only virus stocks with no or variant frequencies below 20% of all sequence reads were included in downstream infection experiments. Virus isolation and all SARS-CoV-2-related infection experiments were performed under Biosafety Level 3 (BSL-3) conditions with enhanced respiratory personal protection equipment. RVFV cl.13, an RVFV-mutant devoid of the IFN induction antagonist NSs, was applied as an induction control in gene expression analysis experiments [64,65].

### Plasmids

Codon-optimized, carboxyl-terminally tagged spike cDNAs in pCG were generated using pCG-SARS-CoV-2 spike Wuhan as a template [46] in which the amino terminus was repaired and D^614^G was introduced via site-directed mutagenesis. The individual VOC Alpha-characteristic mutations were introduced individually and in combination by site-directed mutagenesis. All constructs were confirmed by Sanger sequencing.

### Virus isolation

Virus was isolated from naso- or oropharyngeal swabs using Vero E6 cells. Cells were seeded at a density of 175,000 cells per well in 24-well plates 1 day prior to isolation. For virus isolation, the medium was removed and cells were rinsed once with 1× PBS (Thermo Fisher Scientific) and inoculated with 200 μl of swab sample. After 1 hour incubation, 800 μl of isolation medium (DMEM, supplemented with 2% FBS, 1% penicillin-streptomycin, and 1% amphotericin B, Thermo Fisher Scientific) was added to each well. Cells were monitored for CPE every day. Four days postinoculation, viral RNA was isolated and quantified from the supernatant as described below. Isolation success was determined when CPE was detectable and viral RNA concentrations were above a threshold of 100,000 genome equivalents per μl. Virus stocks were produced from all positive cultures.

### Virus stock production

Vero E6 cells were seeded in T175 tissue culture flasks allowing the cells to reach 90% confluence on the following day. Cells were washed once with PBS and inoculated with 100 μl of low passage (passage 1–2) virus stock solution (approximately 1,000,000 PFU per ml) in 20 ml virus infection medium (DMEM supplemented with 2% FBS, 1% NEAA, 1% sodium phosphate). Three days postinoculation, supernatant was harvested and virus particles were purified from cytokines and concentrated using Vivaspin 20 (Sartorius, filtration units with a size exclusion of 100 kDa) according to the manufacturer’s instructions. Virus concentrate was resuspended in 2 to 3 ml PBS, diluted 1:2 in virus preservation medium (0.5% gelatine in OptiPRO serum-free medium) and stored at −80°C. Infectious titers were determined in 3 independent plaque titration experiments and viral RNA concentration was quantified by real-time RT-PCR (E gene assay). All stocks were sequenced by next-generation sequencing methods and the absence of additional mutations was confirmed to occur in less than 20% of the virus-specific reads.

### Virus infection and virus growth kinetics in cell cultures

Vero E6, Caco-2, NCI-H1299, and A549 cells were seeded at a density of 350,000 cells per ml and Calu-3 cells at a density of 500,000 cells per ml 1 day prior to infection. For infection, virus stocks were diluted in OptiPRO SFM (Thermo Fisher Scientific) serum-free medium according to the desired MOI. For virus adsorption, 200 μl (24-well) or 1 ml (6-well) of virus master mix was added to the cells and incubated at 37°C in a 5% CO_2_ atmosphere with 95% humidity. After 1 hour, virus dilutions were removed, cells were washed 3 times with PBS, and wells were refilled with DMEM infection medium. To determine infectious titers, supernatants were harvested at the indicated time points, and diluted 1:2 in virus preservation medium and stored at −80°C until conducting plaque titration assay.

### Infection of bronchial epithelial cells

hBAEC (SmallAir) cultures applied in Fig 2B were obtained from Epithelix Sàrl, Geneva Switzerland. All other experiments were conducted with hBAECs isolated from explanted lungs, which were obtained from the Hannover Lung Transplant Program after patients informed consent, ethical vote 2923–2015. For isolation of hBAECs, human bronchial tissue was cut into small pieces in Hank’s buffer (Thermo Fisher Scientific) containing 0.18% protease XIV and incubated for 2 hours at 37°C. After thorough pipetting with a 25/50 ml serological pipette, cell solution was filtered through a 100 μm cell strainer (Corning) to remove clumps and 10 ml RPMI supplemented with 10% FCS (Thermo Fisher Scientific) was added. After centrifugation for 10 minutes at 500 *g* and 4°C, supernatant was removed and cells were resuspended in SAGM (Lonza) + Primocin (InvivoGen) + Penicillin-Streptomycin (P/S) (Sigma-Aldrich). For air–liquid interface cultures, 200,000 hBAECs were seeded onto PureCol- (Advanced BioMatrix) coated 12-well inserts (Greiner Bio-One) in SAGM + Primocin + P/S. Culture medium in apical and basal chamber was changed to PneumaCult-ALI medium (STEMCELL Technologies) 48 hours postseeding. Air-lift was performed 48 hours later by gently removing medium from the apical chamber. Homogenous distributed cilia were visible latest 3 weeks after air-lift and inserts were used for infections.

For infection, the apical surface was washed up to 5 times with 200 μl PBS to remove mucus. Virus stocks were diluted in OptiPRO and hBAEC were infected with an absolute infectious dose of 50,000 PFU (SmallAir) or 100,000 PFU (in-house hBAECs). Cells were incubated for 1.5 hours at 37°C in a 5% CO_2_ atmosphere with 95% humidity. After adsorption, virus dilutions were removed and the cells were washed 3 times with 200 μl PBS. Samples were taken at the indicated time points from the apical surface by applying 200 μl PBS to the cells. PBS was incubated on the cells for 10 minutes at 37°C to ensure that virus particles diffuse into the solution before collecting the supernatant samples. Basolateral medium (SmallAir Medium for SmallAir cultures or PneumaCult-ALI for in-house hBAECs) was exchanged every 48 hours.

### Infection of nasal airway epithelial cells

Primary human nasal airway epithelial cells (hNAECs) were collected from healthy individuals by nasal brushings. Informed consent was obtained from all volunteers and the study was approved by the Charité Ethics Committee (EA2/161/20, EA2/066/20). Cultivation of hNAECs was performed as previously described [66]. Briefly, cells were expanded using the conditionally reprogrammed cell (CRC) culture method, then p.2 or p.3 cells were seeded on porous Transwell or Snapwell 1.1 cm^2^ supports (Corning) in UNC-ALI medium and differentiated at air–liquid interface for at least 3 weeks prior to infection. Approximately 200,000 hNAECs were infected with SARS-CoV-2 B.1 or VOC Alpha/v2 at an MOI of 0.1 in 150 μl PBS containing 0.3% BSA for 1 hour at 37°C in a 5% CO_2_ atmosphere with 95% humidity. Afterwards, cells were washed apically with PBS and fresh medium was added basolaterally. Samples were taken at the indicated time points from the apical surface by applying 100 μl PBS to the cells. PBS was incubated on the cells for 30 minutes at 37°C to ensure that virus particles diffuse into the solution before collecting the supernatant samples. A 250 μl sample was taken from the basolateral side and medium was replenished. All samples were titrated on Vero E6 cells by plaque assay to determine infectious titers.

### Infection of lung organoids

Human lung organoids were established as previously published [67]. Informed consent was obtained from all volunteers and the study was approved by the Charité Ethics Committee (project 451, EA2/079/13). For infection, Matrigel was liquefied and removed on ice and organoids were broken up by repeated resuspension using a disposable syringe with needle (27G). Virus stocks were diluted at the desired MOI in organoid infection medium (Advanced DMEM/F12 with 10 mM HEPES and 1× GlutaMax, Thermo Fisher Scientific) and dilution was inoculated for 1 hour at 37°C and in a 5% humidified CO_2_ atmosphere. After infection, organoids were washed twice with PBS and resuspended in Cultrex 3-D Culture Matrix (R&D Systems) for 30 minutes before organoid medium (as described above) was added. Samples were taken from supernatants at the indicated time points and analyzed by plaque titration assay as described previously.

### Infection of intestinal organoids

Human normal colon organoids were established from noncancerous parts of colorectal cancer resection tissue and cultured as previously published [68] under the ethics approval no. EA4/164/19 (to Markus Morkel). For infection studies, organoids were harvested; Matrigel (Corning, #356231) was removed by resuspension and centrifugation. Subsequently, organoids were infected in solution with an MOI of 0.05 (SARS-CoV-2 B.1 and VOC Alpha strains) at 37°C for 1 hour. Infected organoids were seeded in Matrigel and were supplemented with medium. Samples were taken from supernatants at 24, 48, and 72 hours postinfection and analyzed by real-time RT-PCR as described [69].

### Synchronized infection experiments

Synchronized infection experiments were performed to determine entry efficiency of the virus variants. Infection of cells was performed on ice and cells were immediately transferred to 4°C for 1 hour after virus dilutions were added to ensure synchronized virus uptake and start of replication. After virus adsorption, cells were washed 5 times with PBS to remove excess virus particles. Cells were lysed either immediately or incubated with infection medium until 4 or 6 hours postinfection. At the indicated time points, medium was removed and cells were lysed with MagNA Pure 96 external lysis buffer (Roche, Penzberg, Germany). Isolation of RNA from cell lysates and quantitative RT-PCR on subgenomic nucleocapsid RNA was performed as described elsewhere [69,70]. Entry inhibitors were dissolved in DMSO in the indicated concentrations, added 1 hour prior to virus infection, and were supplied for the entire duration of the experiment. Pretreatment of virions with TPCK-treated trypsin (Sigma Aldrich) or saliva (1:10 diluted in serum-free medium) was performed for 1 hour at 37°C prior to virus infection.

### Plaque assay

Plaque assay was performed to determine the titer of stocks and the infectious dose of supernatants harvested from infected cells. A total of 175,000 Vero E6 cells were seeded in a 24-well plate 1 day prior to infection. After washing the cells once with PBS, cells were inoculated in duplicates with 200 μl of 1:10 serially diluted cell culture supernatants from infected cells. After adsorption for 1 hour at 37°C, the virus dilutions were removed and 500 μl of a highly viscous overlay (1:1 mix of 2.4% avicel and 2× concentrated DMEM supplemented with 5% FBS, 2% NEAA, and 2% NaP) was added to each well. The overlay was discarded at 3 days postinfection. Cells were fixed for 30 minutes in 6% formaldehyde, washed once with PBS, and stained for 15 minutes with crystal violet solution. Plaques were counted from 1 to 2 dilutions for which distinct plaques (in a range between 1 and 100 plaques) were detectable. To calculate the titer, the number of all plaques counted was divided by the respective inoculation volume and multiplied with the inverse dilution factor.

Unless otherwise indicated, Vero E6 were applied for virus stock production and titration. Vero E6-determined virus titers were unaffected by the smaller plaque morphology of VOC Alpha in Vero E6 as demonstrated by comparative titration of B.1 and VOC Alpha-stocks on Vero E6 and Calu-3 (S11A Fig). Inocula were back-titrated on Vero E6 cells to ensure that equal amounts of virus were used for infection. Titers of all inocula are summarized in S2 Table.

To ensure that equal doses were applied, infectious titers (PFU/ml) were additionally compared to genome equivalents (GE/ml). For 5 VOC Alpha- and 12 B.1 stocks, neither GE nor infectious titer were significantly different between both viruses (S11B Fig).

### TCID_50_

Where indicated, virus titers were determined as TCID_50_ per ml in confluent Vero E6 and/or Calu-3 cells in 96-well microtiter plates. Briefly, supernatant was serially diluted (1:10) in quadruplicates and cells were infected with 50 μl virus-containing dilution as previously described in the plaque assay section. After adsorption, cells were washed once with PBS and supplemented with 100 μl DMEM containing 2% FCS. At 3 days postinfection, medium was removed and cells were fixed in 6% formaldehyde for 30 minutes, washed once with PBS, and stained for 15 minutes with crystal violet solution. For analysis, all wells were either considered virus positive or virus negative, depending on the presence or absence of a CPE, respectively. The TCID_50_ was then calculated by the method of Reed and Muench [71,72].

### Competition assay

Calu-3 cells were infected in 24-well plates with a mixture of 2 SARS-CoV-2 variants, using 3 different ratios (1:1 and 9:1 and 1:9) and an initial, total infectious dose of 10,000 PFU (corresponding to an MOI of 0.04). Serial infections were performed by sampling the supernatant of the previous passage at 24 hours postinfection and infecting naive Calu-3 cells with a 1:50 dilution of this sample. This process was repeated until completion of 5 passages. As a control for genome stability over 5 passages, single infections were performed. Viral RNA was isolated from the initial inoculum and from the supernatant of all 5 passages. To confirm that the virus is detectable over 5 passages, concentration of viral RNA was analyzed by quantitative RT-PCR (E gene assay) from each passage. To determine the variant frequency in each passage, RNA samples were sequenced using next generation sequencing techniques (Illumina technology). For virus sequence analysis, the raw sequences were trimmed, matched, and presorted for SARS-CoV-2-specific sequence reads. The processed sequence reads were mapped to the BetaCoV/Munich/ChVir984/2020 genome (here referred to as SARS-CoV-2 2019-nCoV strain) in Geneious (version 9.1.8). The 2 virus variants in each sample were distinguished from each other by their lineage-specific mutations. For evaluation, the relative variant frequencies were calculated for each variable position. This was conducted for a total of 19 lineage-specific mutations, which were distributed over the entire genome.

### Next-generation sequencing of virus stocks and for competition assay

Viral RNA was extracted using MagNA Pure 96 System (Roche, Penzberg, Germany) according to the manufacturers’ recommendations. The RNA-seq library was prepared from viral RNA extracts using the KAPA RNA HyperPrep Kit (Roche, Penzberg, Germany) and KAPA DI adaptors according to the manufacturers’ instructions. The RNA library was subjected to next-generation sequencing on a NextSeq System (Illumina) using a NextSeq 500/550 v2.5 Kit (Illumina). Sequences were analyzed using the geneious software, version 9.1.8, and sequence reads were assembled by mapping reads to the respective reference sequences.

### Live cell imaging

For live cell imaging, Vero E6 cells were infected at an MOI of 0.01 or 0.001 for 1 hour with subsequent replacement of the inoculum with full culture medium. Cells were imaged with the Zeiss LSM800 Airyscan Confocal Microscope over 72 hours with 30-minute intervals in a 5% CO_2_ supplemented, humidified environment. Images were analyzed for the onset of visible cytopathic effects and merged using Zeiss ZEN Blue 3.0 and ImageJ 1.53c.

### In vivo infections

#### Animals

Animal procedures were performed according to the European Guidelines for Animal Studies after approval by the relevant state authority (Landesamt für Gesundheit und Soziales, Berlin, permit number 0086/20). Per group, 9 male and female Roborovski dwarf hamsters (*Phodopus roborovskii*) obtained via the German pet trade were used. Animals were housed in groups of 3 to 6 hamsters in GR-900 IVC cages (Tecniplast, Buguggiate, Italy) and provided with bountiful enrichment and nesting materials (Carfil, Oud-Turnhout, Belgium). Hamsters of the same sex were randomly distributed into experimental groups and individually marked with a subcutaneously implanted IPTT-300 transponder (BMDS, Seaford (DE), USA) that allows remote identification and measurement of body temperature.

#### Infection and transmission experiments

To determine virus production in vivo, 9 hamsters were inoculated with 100,000 PFU of either B.1 or VOC Alpha, as previously described [73]. Briefly, anaesthetized hamsters received 100,000 PFU SARS-CoV-2 in 20 μL MEM by intranasal instillation. At 24 hours postinoculation, contact to uninfected hamsters was enabled by placing 3 infected animals into a cage containing 3 uninfected animals of the same sex. Hamsters were monitored twice daily for clinical signs of infection. Body weight and temperature was recorded daily. Hamsters were sacrificed to determine virological parameters of infection on days 2, 3, 5, and 7 postinfection or contact, or once an individual reached a defined humane endpoint.

#### Virus titrations, RNA extractions, and RT-qPCR

To determine virus titers from 50 mg of lung tissue, tissue homogenates were prepared using a bead mill (Analytic Jena) and 10-fold serial dilutions were prepared in MEM, which were then added to Vero E6 cells in 12-well plates. The dilutions were removed after 2 hours and cells were overlaid with 1.25% microcrystalline cellulose (Avicel) in MEM supplemented with 10% FBS and penicillin/streptomycin. Two days later, cells were formalin fixed, stained with crystal violet, and plaques were counted. RNA was extracted from 25 mg of lung homogenates and oropharyngeal swabs using the innuPREP Virus RNA kit (Analytic Jena). Viral RNA copies were quantified in 10% of the obtained eluate volume with a 1-step RT-qPCR reaction using a standard curve and the Luna Universal Probe One-Step RT-qPCR kit (New England Biolabs) and previously published TaqMan primers and probe [69] on a StepOnePlus RealTime PCR System (Thermo Fisher Scientific).

### Reverse genetics

We employed the previously described in-yeast transformation-associated recombination (TAR) cloning method [74] for the generation of infectious SARS-CoV-2 VOC Alpha cDNA clones. Overlapping DNA fragments were obtained by first strand cDNA synthesis of viral RNA extracts from infected Vero E6 cells using SuperScript III reverse transcriptase (Invitrogen) followed by a nested Phusion PCR (Invitrogen). Primers for TAR fragment generation were used as previously described [74] with VOC Alpha-specific deviations for 2 fragments, as specified in S3 Table. For generation of the D^614^G mutant we performed site-directed mutagenesis PCR (New England Biolabs) on synthetic viral subgenomic fragments cloned into pUC57 vectors [74]. Assembly of purified DNA fragments was performed by TAR cloning as previously described [74]. Clones were screened for correctly assembled DNA fragments by multiplex PCR using the QIAGEN Multiplex PCR kit (QIAGEN) according to the manufacturers’ instructions. Clones tested positive for all junctions were expanded, plasmid DNA was extracted, linearized and subjected to T7-based in vitro RNA transcription (Thermo Fisher Scientific). Capped viral RNA was electroporated into baby hamster kidney cells, and supernatant was subsequently transferred to Vero E6 cells 1 day after electroporation for stock production. Successful virus rescue was confirmed by SARS-CoV-2-specific RT-PCR. Virus stocks were harvested 3 days postinfection, purified and deep sequenced as described above.

### Isolation of viral RNA and quantitative real-time RT-PCR assay

For isolation of viral RNA, 50 μl of supernatant was diluted in 300 μl of MagNA Pure 96 external lysis buffer (Roche, Penzberg, Germany). All samples were heat inactivated for 10 minutes at 70°C prior to export from the BSL-3. Isolation and purification of viral RNA was performed using the MagNA Pure 96 System (Roche, Penzberg, Germany) according to the manufacturers’ recommendations. Viral RNA was quantified using real-time RT-PCR (E gene assay) as previously described [69].

### Isolation of total RNA, cDNA synthesis, and quantitative PCR

For extraction of total RNA, the MagNa Pure 96 System (Roche, Penzberg, Germany) was used according to the manufacturers’ instructions. Briefly, cells were washed once with PBS before 350 μl of external lysis buffer (Roche, Penzberg, Germany) was added to the cells. Lysed cells were resuspended 2 to 3 times and transferred to the reaction tube. Samples were heat inactivated for 10 minutes at 70°C and exported from the BSL-3 laboratory. For quantitative RT-PCR, a 12.5-μl reaction with 2.5 μl RNA was done with the SuperScript III 1-step reverse transcriptase-PCR system (Invitrogen) with the Platinum Taq DNA polymerase according to the manufacturers’ protocol and the primers indicated in S4 Table. Probes contained a 5′ FAM-520 reporter dye and a ZEN/Iowa Black FQ 3′ quencher (Integrated DNA Technologies). The RT-PCR was performed using a thermocycling protocol with reverse transcription for 15 minutes at 55°C and a subsequent denaturation step for 2 minutes at 95°C to restore *Taq* DNA polymerase activity, followed by PCR amplification by 45 cycles of 95°C for 15 seconds and 58°C for 30 seconds. Fluorescence signals were detected after the elongation step of each cycle. The mean fold change in gene expression was calculated by the delta-delta ct method and by using expression of TATA-binding protein (TBP) as a reference.

To determine early virus replication, a quantitative RT-PCR targeting the subgenomic RNA encoding the nucleocapsid (sgN) was performed. Viral RNA was extracted from cell lysates, which were previously lysed by external lysis buffer (Roche, Penzberg, Germany) as described above. RT-PCR was done with the following primers and probe: nCoV sgN Fwd: 5′-CGA TCT CTT GTA GAT CTG TTC TC-3′, nCoV sgN Rev: 5′-CAG TAT TAT TGG GTA AAC CTT GG-3′ and nCoV sgN prb: 5′-56-FAM/ CAG TAA CCA GAA TGG AGA ACG CAG /3BHQ-1-3′ [70]. For quantification, values were normalized to the housekeeping gene TBP levels by delta ct method.

### RNA-Seq analysis

Calu-3 cells were infected in triplicates with B.1 or VOC Alpha viruses with MOI 2 and harvested 24 hours postinfection. Total RNA was extracted with MagNa Pure 96 System (Roche, Penzberg, Germany) and libraries produced using the Kapa RNA HyperPrep (Roche Molecular Diagnostics, Basel, Switzerland) were pooled at equimolar ratio and paired-end sequenced using the 150-cycle NextSeq reagent v3 HighOutput cartridge (Illumina, San Diego, USA) according to manufacturer’s instructions. The produced reads were trimmed using AdapterRemoval [75] v2.3.2, merged using FLASH [76] v1.2.11 and reads mapping against the human genome were removed using bwa [77] v0.7.17 and samtools [78] v1.12. Reads that mapped against the transcriptional regulatory sequence (TRS) ‘AUUAAAGGUUUAUACCUUCCCAGGUAACAAACCAACCAACUUU CGAUCUCUUGUAGAUCUGUUCUCUAAACGAAC’ using Geneious Mapper were extracted, and the region matching the TRS was removed. The resulting reads were then mapped against the SARS-CoV-2 genome NC_045512 using Geneious Mapper and the number of reads mapping immediately after the genomic TRS or at the start of of S, ORF3a, E, M, ORF6, ORF7, ORF8, N, ORF9b, and N* sgRNAs was counted.

### Tat-mediated cell–cell fusion assay

CHO and TZM-bl cells were retrieved from the NIH AIDS Reagent Program and propagated as recommended. CHO cells were transiently transfected with expression plasmids for HIV-1 Tat and individual pCG-spike-HA or empty vector control for 48 hours, using Lipofectamine LTX Reagent with PLUS Reagent (Invitrogen). TZM-bl cells, stably expressing LTR-driven luciferase, were transfected with a plasmid encoding human ACE2 and human myc-TMPRSS2. CHO and TZM-bl cells were cocultured for 8 hours. Subsequently, cells were washed once with PBS, lysed using cell culture lysis buffer (Promega), and Tat-dependent increase of luciferase enzyme activity in cell lysates was determined with the Luciferase Assay system (Promega). Luminometric activity was analyzed with a Mithras luminometer.

### Lentivirus production and transduction experiments

SARS-CoV-2 spike-HA-pseudotyped lentiviral particles were produced in triple-transfected HEK293T cells. Cells were transfected with individual pCG-SARS-CoV-2 spike-HA plasmids, the HIV-1-based packaging plasmid deltaR8.91 [79] and the luciferase transfer plasmid pCSII-luciferase [80] via calcium phosphate precipitation. Virus-containing supernatant was harvested 40 and 64 hours posttransfection and sterile-filtered. Particles were concentrated via ultracentrifugation through a 20% sucrose cushion. Indicated cell lines were transduced for 72 hours with identical p24 capsid equivalents as quantified by immunoblotting of particle lysates. Transduction efficiency was quantified luminometrically 3 days posttransduction. For each biological replicate, individual DNA preparations were used for generation of independent lentiviral pseudotype stocks.

### Immunoblotting

To determine incorporation and processing of spike in lentiviral particles, transduced cells and lentiviral particles were lysed with M-PER Mammalian Protein Extraction Reagent (Pierce) and Triton X-100, respectively. The lysate was mixed with Laemmli buffer and boiled for 10 minutes at 95°C. Proteins were separated on a 10% SDS-PAGE and immobilized on a nitrocellulose membrane (GE Healthcare) using the Trans-Blot Turbo system (BioRad). Blocked membranes were incubated with the following antibodies: mouse anti-HIV-1 p24 capsid (ExBio, 1:1,000), rabbit anti-S2 spike (Novusbio, NB100-56578, 1:1,000), mouse anti-HA (Sigma, H3663, 1:1,400), rabbit anti-tubulin (Cell Signaling Technology, 2144S, 1:1,000). Secondary antibodies conjugated to Alexa680/800 fluorescent dyes were used for detection and quantification by Odyssey Infrared Imaging System (LI-COR Biosciences). Spike processing efficiency was calculated as the percentage of S2 from total spike signal. Relative levels of spike-HA abundance in lentiviral pseudotypes were quantified by calculating the signal intensity of S2-HA per HIV-1 p24 capsid.

To determine the processing and incorporation of spike from infected cells, cells and purified virus particles were lysed with RIPA (Thermo Fisher Scientific) buffer supplemented with complete protease inhibitor cocktail (Roche) for 30 minutes at 4°C. Subsequently, lysates were centrifuged for 15 minutes at 4°C and 15,000 rpm to remove cell debris. The supernatants were mixed with 4× Laemmli buffer, which was supplemented with 10% beta-mercaptoethanol, and lysates were boiled for 10 minutes at 95°C to ensure protein denaturation and virus inactivation. Protein concentration was determined by BCA protein assay (Thermo Fisher Scientific) and 20 μg total protein was loaded. Proteins were separated by SDS-PAGE on a 6% gel and transferred to a nitrocellulose membrane (0.45 μm pore size, GE Healthcare) by Trans-Blot Turbo system (BioRad). Membranes were blocked with 5% dried milk in 0.1% PBS-Tween (0.9% NaCl, 10 mM Tris-HCl [pH 7.5], 0.1% Tween 20) for 30 minutes at room temperature. Blocked membranes were incubated with the following antibodies: rabbit anti-S2 spike (Novusbio, NB100-56578, 1:1,000), rabbit anti-SARS-CoV-2 nucleocapsid (GeneTex, GTX135361, 1:1,000). Secondary antibodies conjugated with horseradish peroxidase (HRP) were used for chemiluminescence-based detection by Fusion Fx7 (Peqlab Biotechnologie GmbH). Detection was performed using SuperSignal West Femto substrate (Thermo Fisher Scientific). Quantification was done by the use of ImageJ 1.48v software. Spike processing efficiency was calculated as the percentage of S2 from total spike signal. Relative levels of spike abundance in concentrated virion preparations were quantified by calculating the signal intensity of S2 per nucleocapsid.

### Flow cytometry analysis

A total of 600,000 Calu-3 cells were seeded in 6-well plates 1 day prior to infection with B.1 and VOC Alpha (MOI of 2). At 24 hours postinfection, supernatant was removed, cells were washed with PBS and trypsinized (Thermo Fisher Scientific). Detached cells were PFA fixed and immunostained for intracellular SARS-CoV-2 nucleocapsid expression using rabbit anti-nucleocapsid antibody (Genetex, GTX635680) in primary antibody staining solution (0.1% Triton-X/PBS) followed by incubation with goat anti-rabbit Alexa-488 secondary antibody dilution (Thermo Fisher Scientific, A11008) for 20 minutes at 4°C. Flow cytometry was performed on a BD FACSCelesta and data were analyzed with FlowJo V10 software.

### MagPix Luminex

To assay cytokine levels in airway epithelial cell supernatant, 25 μl of supernatant were sampled prior to infection and at 24 hours and 48 hours postinfection with SARS-CoV-2 B.1, VOC Alpha/v1, and VOC Alpha/v2. Cytokine quantification was performed using a Human Cytokine/Chemokine/Growth Factor Panel A 48-Plex Premixed Magnetic Bead Multiplex Assay (Merck Millipore), using the Luminex MAGPIX System in 96-well plate format, according to the manufacturer’s instructions. Plate washing steps were performed using HydroFlex Microplate Washer (Tecan). Calibration, verification, and quality control checks were met for all of the analytes. However, Analyte 15 (FGF-2) was omitted because its standard curve had an R^2^ value of 0.82 and Analyte 53 (IL-17F) was omitted because it had a high limit of detection and several extreme outliers from the rest of the dataset. All other analytes were reported.

### PRNT assays

Plaque reduction neutralization tests (PRNTs) were performed as previously described [30,70]. Briefly, heat-inactivated sera were serially diluted starting at 1:40 in OptiPro, mixed 1:1 with 200 μl virus solution containing 200 plaque-forming units of SARS-CoV-2 (strains B.1, VOC Alpha/v1, and B.1.351), and 200 μl of the mix were incubated in duplicates on Vero E6 cells (160,000 cells per well) seeded in 24-well plates on the previous day. After 1 hour incubation at 37°C, the supernatant was discarded and cells were washed with PBS and overlaid with 1.2% Avicel solution in supplemented DMEM. After 3 days at 37°C, the supernatants were removed and cells were inactivated and fixed with a 6% formaldehyde/PBS solution and stained with crystal violet. Serum dilutions with a mean plaque reduction of 50% and 90% are referred to as PRNT_50_ or PRNT_90_. For numerical calculations, titers <40 were set to 20, and titers >1:640 were set to 1:1,280.

### Surrogate neutralization assay

Neutralizing capacity of patients’ sera against B.1, VOC Alpha, and B.1.351 was assessed by a surrogate virus neutralization test (cPass Assay, Medac, Wedel, Germany) as described previously [81,82]. Briefly, sera of infected and vaccinated patients were diluted 1:10 with sample dilution buffer, mixed 1:1 with B.1-HRP-RBD, VOC Alpha-HRP-RBD, and B.1.351-HRP-RBD (provided by Medac, Wedel, Germany) solution, and incubated at 37°C for 30 minutes. Afterwards, the mixture was added to the hACE2-coated plate and incubated at 37°C for 15 minutes. After washing, 3′3,5,5-tetramethylbenzidine solution was added, and the plate was incubated in the dark at room temperature for 15 minutes. Stop solution was then added and the optical density at 450 nm was measured using a Tecan Infinite 200 PRO plate reader. For calculation of the relative inhibition of ACE2/RBD binding, the following formula was applied: inhibition score (%) = (1 − OD value sample/OD value negative control) × 100%. Values below zero were set to zero.

### Data presentation and statistical analysis

If not stated otherwise, bars and symbols show the arithmetic mean of the indicated amount of independent replicates. Error bars indicate SD from at least 3 or SEM from the indicated amount of individual experiments. Statistical analysis was performed with GraphPad Prism (V 8.3.0 or 9.1.2) using 2-tailed unpaired Student *t* tests or for comparing neutralizing activities the Friedman test and Dunn’s multiple comparison unless indicated differently. *P* values <0.05 were considered significant (*), <0.01 (**), <0.001 (***), <0.0001 (****); n.s. = not significant (≥0.05). Only statistically significant results are highlighted in the figures.

## Supporting information

S1 Fig Competition assay, additional targets. Calu-3 cells were infected with a mixture of B.1 and VOC Alpha at indicated ratios (B.1:VOC Alpha/v1 ratio of 1:1, 9:1, and 1:9) with a total infectious dose of 10,000 PFU (corresponding to an MOI of 0.04). After serial passaging, viral RNA from the supernatant was isolated, sequenced, and the relative proportion of B.1- and VOC Alpha-corresponding sequences, discriminated by mutations in NSP3 (**A**), Spike amino acid positions 501 (**B**) and 681 (**C**) was plotted. Data show individual values of triplicates of 1 experiment. MOI, multiplicity of infection; PFU, plaque-forming units; p0-p5, passage 0–passage 5; VOC, variant of concern. See S1 Data.
(TIF)

S2 Fig VOC Alpha fails to escape from neutralizing antibodies. (**A**) Neutralizing titers against the indicated virus strains were determined in PRNTs. Red line indicates median titers per group. (**B**) Inhibition of ACE2/RBD interaction was measured using surrogate virus neutralization assays. Sera were tested using RBD proteins of B.1, VOC Alpha-, and Beta-VOC as indicated. Red lines indicate median values. The same set of samples was measured in (**A**) and (**B**), vaccinees *n* = 19, non-VOC convalescent donors *n* = 50, B1.1.7 patients *n* = 13. PRNT, plaque reduction neutralization test; VOC, variant of concern. See S1 Data.
(TIF)

S3 Fig Relative sgRNA level normalized to total RNA reads and infection efficiency in B.1- and VOC Alpha-infected Calu-3 cells. (**A**) RNA-seq analysis was conducted from total cell lysates that were obtained 24 hours postinfection to quantify sgRNA proportions in SARS-CoV-2-infected cells (MOI of 2). Canonical, as well as ORF9b and N* sgRNAs were quantified from the RNA-seq dataset. Data were normalized to total RNA reads. (**B**, **C**) Number of SARS-CoV-2 nucleocapsid (N)-positive Calu-3 cells was determined by flow cytometry. Calu-3 were left either UI or were infected with B.1 and VOC Alpha (MOI of 2) for 24 hours, permeabilized and immunostained with rabbit-anti-SARS-CoV-2 nucleocapsid antibody, followed by goat anti-rabbit Alexa 488 secondary antibody. (**B**) Percentage of SARS-CoV-2 N-positive cells. (**C**) Gating strategy of living-, single-, and N-positive cells is depicted for UI, B.1-, and VOC Alpha-infected cells. MOI, multiplicity of infection; SARS-CoV-2, Severe Acute Respiratory Syndrome Coronavirus 2; sgRNA, subgenomic RNA; UI, uninfected; VOC, variant of concern. See S1 Data.
(TIF)

S4 Fig Growth, replication, and gene expression in infected, parental, and ACE2/TMPRSS2-expressing A549 cells. (**A**) Virus growth was quantified in parental and ACE2/TMPRSS2-expressing A549 cells infected at an MOI of 0.01. Supernatant collected at the respective time points was titrated by plaque assay on Vero E6 cells. Growth kinetic experiments were performed once in triplicates. (**B**) Expression of cell-associated *envelope* was determined in parental and ACE2/TMPRSS2-expressing A549 cells at 24 and 48 hours postinfection by Q-RT-PCR. (**C**) Expression of cell-associated sgN in Calu-3 cells at 24 and 48 hours postinfection was determined by Q-RT-PCR. TBP was used for normalization. (**D**) Expression of the indicated genes was determined by Q-RT-PCR. Shown is the mean fold change +/− SD of 3 biologically independent experiments that were each conducted in quadruplicates. RVFV cl.13, which is devoid of its IFN antagonist NSs, was included for the analysis of expression of *IFNs* and *ISGs*. A^+^/T^+^, ACE2/TMPRSS2-expressing A549 cells; GE, genome equivalents; par, parental; IFN, interferon; MOI, multiplicity of infection; Q-RT-PCR, quantitative real-time PCR; RVFV cl.13, Rift Valley Fever Virus clone 13; sgN, subgenomic nucleocapsid; TBP, TATA-binding protein. See S1 Data.
(TIF)

S5 Fig B.1 and VOC Alpha spike are expressed at similar levels. (**A**) Expression of total spike in HEK 293T cells. Symbols represent independently performed experiments. (**B**) Vero E6 cells were infected with SARS-CoV-2 (MOI 5). Cells and virus-containing supernatants were harvested at 48 hours postinfection and processed for detection of spike by immunoblotting. (**C**) Expression of total spike in Vero E6 cells was quantified by the use of ImageJ 1.48v. MOI, multiplicity of infection; SARS-CoV-2, Severe Acute Respiratory Syndrome Coronavirus 2; VOC, variant of concern. See S1 Data.
(TIF)

S6 Fig VOC Alpha spike is not superior in mediating entry compared to B.1 spike. (**A**) Calu-3 cells were transduced for 72 hours with increasing amounts of lentiviral particles (0.1 μl, 1 μl, and 10 μl) pseudotyped with either B.1 or VOC Alpha spike proteins. Pseudotype entry was analyzed luminometrically in cell lysates. (**B**) Calu-3 cells were pretreated with 25 μM MDL28170 (Cathepsin L inhibitor), 25 μM pitstop II (clathrin inhibitor), 100 μM Camostat (TMPRSS2 inhibitor), or 15 μM CMK (furin inhibitor), infected and entry efficiency was determined by sgN Q-RT-PCR. (**C**) Calu-3 cells were infected with Calu-3-derived virus stocks. Entry efficiency was determined by sgN-specific Q-RT-PCR from cell lysates at 4 hours postinfection. Cam, Camostat mesylate; CatL, Cathepsin L; DMSO, Dimethylsulfoxid; PS: PitStop; Q-RT-PCR, quantitative real-time PCR; sgN, subgenomic nucleocapsid; TMPRSS2, transmembrane protease serine subtype 2; VOC, variant of concern. See S1 Data.
(TIF)

S7 Fig Enhanced, VOC Alpha spike-dependent replication in NCI-H1299 cells. (**A**) Virus growth of B.1, VOC Alpha/v2, and Delta isolates (MOI 0.01) was quantified in Calu-3 cells. (**B**) Virus growth of B.1, VOC Alpha/v2, and Delta isolates (MOI 0.01) was quantified in hBAECs. (**C**, **D**) Vero E6 (**C**) and Calu-3 (**D**) cells were infected with rB.1, rB.1/Alpha spike, and rAlpha/B.1 spike (MOI 0.01) and supernatant was titrated on Vero E6 cells. The growth experiment in Vero E6 cells was performed once in duplicates. Growth experiments in Calu-3 cells was performed once in triplicates. Dashed horizontal lines indicate the lower limit of detection of the plaque assay. hBAEC, human bronchial airway epithelial cell; MOI, multiplicity of infection; VOC, variant of concern. See S1 Data.
(TIF)

S8 Fig SARS-CoV-2 spread requires ACE2 in NCI-H1299 cells although ACE2 levels are undetectable at the protein level. (**A**) ACE2 expression levels of the indicated NCI-H1299 cells, Caco-2 and Calu-3 were analyzed by immunoblotting. Beta-actin was used as a loading control. (**B**) ACE2 expression levels of the indicated NCI-H1299 cells were determined by flow cytometry. % numbers of ACE2-positive NCI-H1299 cells are indicated in the respective dot plots. (**C**) Vero E6 (left), Calu-3 (middle), and NCI-H1299 (right) cells were pretreated with 4 different anti-ACE2 antibodies (each applied at final concentration of 20 μg/ml) for 1 hour prior to infection with B.1- and VOC Alpha isolates (MOI of 0.01). At 24 hours postinfection, viral replication was quantified from the supernatant by the use of E-gene assay. Replication was normalized to the respective untreated cells. Results from 1 experiment, conducted in triplicates, are shown. (**D**) Calu-3 (left) and NCI-H1299 (right) cells were pretreated with 20 μg/ml anti-ACE2 antibody for 1 hour prior to infection with B.1 and VOC Alpha/v2 isolates (MOI of 0.01). At 48 hours postinfection, viral replication was quantified from the supernatant by the use of E-gene assay. Results from 2 independently performed experiments, each conducted in triplicates, are shown. (**E**) Sequencing chromatogram covering the *ACE2* gene locus of NCI-H1299 and ACE2-KO-H1299 cells, expressing ACE2 protein from aa no. 220 ff. Knock-out of *ACE2* in ACE2-KO-H1299 cells was induced by CRISPR/Cas9 technology by the use of a synthetic guide RNA (sgRNA) targeting the genomic *ACE2* region as indicated in the chromatogram. A frameshift mutation was generated through the insertion of 2 nucleotides, which leads to the expression of a truncated ACE2 protein (from aa 225 ff) due to a premature stop codon in the *ACE2*-encoding reading frame (black boxes). (**F**) Virus replication of B.1 and VOC Alpha was investigated on parental-NCI-H1299 (left) and respective ACE2-KO (right) cells. Cells were infected (MOI 0.01) and genome equivalents were determined by E gene assay from the supernatants at indicated time points. Two independent experiments, each in triplicates, were performed and are indicated by symbols. (**G**) ACE2-KO-H1299 cells were transiently *trans*-complemented with human ACE2 or empty vector control plasmid prior to infection with B.1 and VOC Alpha (MOI 0.01). Supernatants of indicated time points were titrated on Vero E6 cells. The experiment was performed once in triplicates. (**H**) Calu-3 cells were pretreated with 0–100 μM camostat mesylate prior to infection (MOI 0.01) with B.1 and VOC Alpha. Virus replication was determined from the supernatant by the use of E gene assay at 48 hours postinfection. The experiment was performed once in triplicates. (**I**) NCI-H1299 cells were transduced with lentiviral particles pseudotyped with indicated spike proteins. Pseudotype entry was analyzed luminometrically in cell lysates. Data from 2 biological replicates, each performed in triplicates, are shown. White symbols represent arithmetic means of the biological replicates. aa, amino acid; KO, knock-out; MOI, multiplicity of infection; Non-transd., Non-transduced; SARS-CoV-2, Severe Acute Respiratory Syndrome Coronavirus 2; VOC, variant of concern. See S1 Data.
(TIF)

S9 Fig Enhanced stability of VOC Alpha virion infectivity in the supernatant of infected NCI-H1299 cells. NCI-H1299 and Calu-3 cells were infected (MOI of 0.01) with either B.1 and VOC Alpha isolates (**A**, **B**) or rB.1, rB.1/Alpha spike, and rAlpha/B.1 spike (**C**, **D**). Supernatants were collected at 24, 48, and 72 hours postinfection and titrated by plaque assay on Vero E6 cells to determine PFU/ml. Genome equivalents (GE/ml) were determined by E gene assay. Two independent experiments, each conducted in triplicates, were performed. Gray numbers indicate mean relative virus infectivity relative to B.1 or rB.1, respectively, of the independent experiments. Bars represent arithmetic means of independent experiments. Graphs in (**A**, **B**) depicting relative particle infectivity at 48 hours postinfection and graphs in (**C**, **D**) depicting relative particle infectivity at 72 hours postinfection are additionally shown in the main figures. GE, genome equivalents; MOI, multiplicity of infection; PFU, plaque-forming units; VOC, variant of concern. See S1 Data.
(TIF)

S10 Fig Similar abundance of sgN RNAs, genome replication, and low but similar expression of IFNs, proinflammatory cytokines, and ISGs in B.1 and VOC Alpha-infected NCI-H1299 cells. NCI-H1299 cells were infected with B.1 or VOC Alpha (MOI of 2), and viral replication, viral transcription, and expression of innate immune genes were determined by Q-RT-PCR from cell lysates at 24 and 48 hours postinfection. (**A**) Expression of cell-associated *envelope*. (**B**) Expression of cell-associated sgN RNA. *TBP* was used for normalization. (**C**) Expression of the indicated genes was determined by specific Q-RT-PCR. *TBP* was used for normalization. Shown is the mean fold change +/− SD of 3 biologically independent experiments that were each conducted in quadruples. RVFV cl.13, which is devoid of its IFN antagonist NSs, was included for the expression of IFNs, ISGs, and pro-inflammatory cytokines. GE, genome equivalents; IFN, interferon; ISG, IFN-stimulated gene; Q-RT-PCR, quantitative real-time PCR; RVFV cl.13, Rift Valley Fever Virus clone 13; sgN, subgenomic nucleocapsid; TBP, TATA-binding protein. See S1 Data.
(TIF)

S11 Fig Evaluation of titration method on Vero E6 and Calu-3 cells for the calibration of B.1 and VOC Alpha seeding doses and comparison of E gene copy numbers versus infectious units (determined by Q-RT-PCR and plaque assay on Vero E6 cells) in SARS-CoV-2 stocks. (**A**) No significant differences in the titers were observed when titers of B.1 and VOC Alpha SARS-CoV-2 stocks were compared on Calu-3 versus Vero E6 using TCID_50_ titration method. Although plaque morphology of B.1- and VOC Alpha-infected Vero E6 cells differ, Vero E6 cells are suitable to determine titers by plaque titration assay. *N* = 3 biologically independent experiments each conducted in triplicates. (**B**) Overview on virus infectivity and viral RNA concentrations, determined by plaque assay (log_10_ PFU/ml) and E gene assay (log_10_ GE/ml), of all virus stocks is shown. Direct comparison of all B.1 and VOC Alpha stocks. Statistical analysis was conducted between both viruses for genomic E gene and infectious titers, respectively. Stocks applied in gene expression analysis (triangle) and growth kinetics (square) are highlighted by symbols. GE, genome equivalents; n.s., not significant; PFU, plaque-forming units; Q-RT-PCR, quantitative real-time PCR; SARS-CoV-2, Severe Acute Respiratory Syndrome Coronavirus 2; VOC, variant of concern. See S1 Data.
(TIF)

S1 Table Salivary samples from healthy donors applied in this study. (XLSX)

S2 Table Inoculum data from all experiments conducted in this study. (XLSX)

S3 Table Primers used for reconstruction of recombinant SARS-CoV-2. (XLSX)

S4 Table Primers used for quantitative RT-PCR. (XLSX)

S1 Movie Delayed cytopathic onset of VOC Alpha SARS-CoV-2 infection. Vero E6 cells were infected with B.1, VOC Alpha/v1, and VOC Alpha/v2 (MOI 0.01). Onset of CPE was monitored by live cell imaging until 70 hours postinfection. CPE, cytopathogenic effect; MOI, multiplicity of infection; SARS-CoV-2, Severe Acute Respiratory Syndrome Coronavirus 2; VOC, variant of concern.
(MP4)

S2 Movie Delayed cytopathic onset of VOC Alpha SARS-CoV-2 infection. Vero E6 cells were infected with B.1, VOC Alpha/v1, and VOC Alpha/v2 (MOI 0.001). Onset of CPE was monitored by live cell imaging until 70 hours postinfection. CPE, cytopathogenic effect; MOI, multiplicity of infection; SARS-CoV-2, Severe Acute Respiratory Syndrome Coronavirus 2; VOC, variant of concern.
(MP4)

S1 Raw Images Raw immunoblots corresponding to indicated figures. (PDF)

S1 Data Raw numeric values corresponding to plotted data in corresponding figures. (XLSX)

## Abbreviations

AEC: airway epithelial culture
BSL-3: Biosafety Level 3
CPE: cytopathogenic effect
CRC: conditionally reprogrammed cell
DMEM: Dulbecco’s Modified Eagle’s Medium
FBS: fetal bovine serum; GE, genome equivalents
HAE: human airway epithelial
hBAEC: human bronchial airway epithelial cell
hNAEC: human nasal airway epithelial cell
HRP: horseradish peroxidase
IFN: interferon; ISG, IFN-stimulated gene
MOI: multiplicity of infection
PBS: phosphate buffered saline
PRNT: plaque reduction neutralization test
RVFV cl.13: Rift Valley Fever Virus clone 13
SARS-CoV-2: Severe Acute Respiratory Syndrome Coronavirus 2
sgRNA: subgenomic RNA
TAR: transformation-associated recombination
TBP: TATA-binding protein
TMPRSS2: transmembrane protease serine subtype 2
TRS: transcriptional regulatory sequences
VOC: variant of concern
