# Supplementary figures and images for: SARS-CoV-2 variant Alpha has a spike-dependent replication advantage over the ancestral B.1 strain in human cells with low ACE2 expression
Source: PLoS Biol. 2022 Nov 16;20(11):e3001871. doi: 10.1371/journal.pbio.3001871 (PMC9710838; doi:10.1371/journal.pbio.3001871)

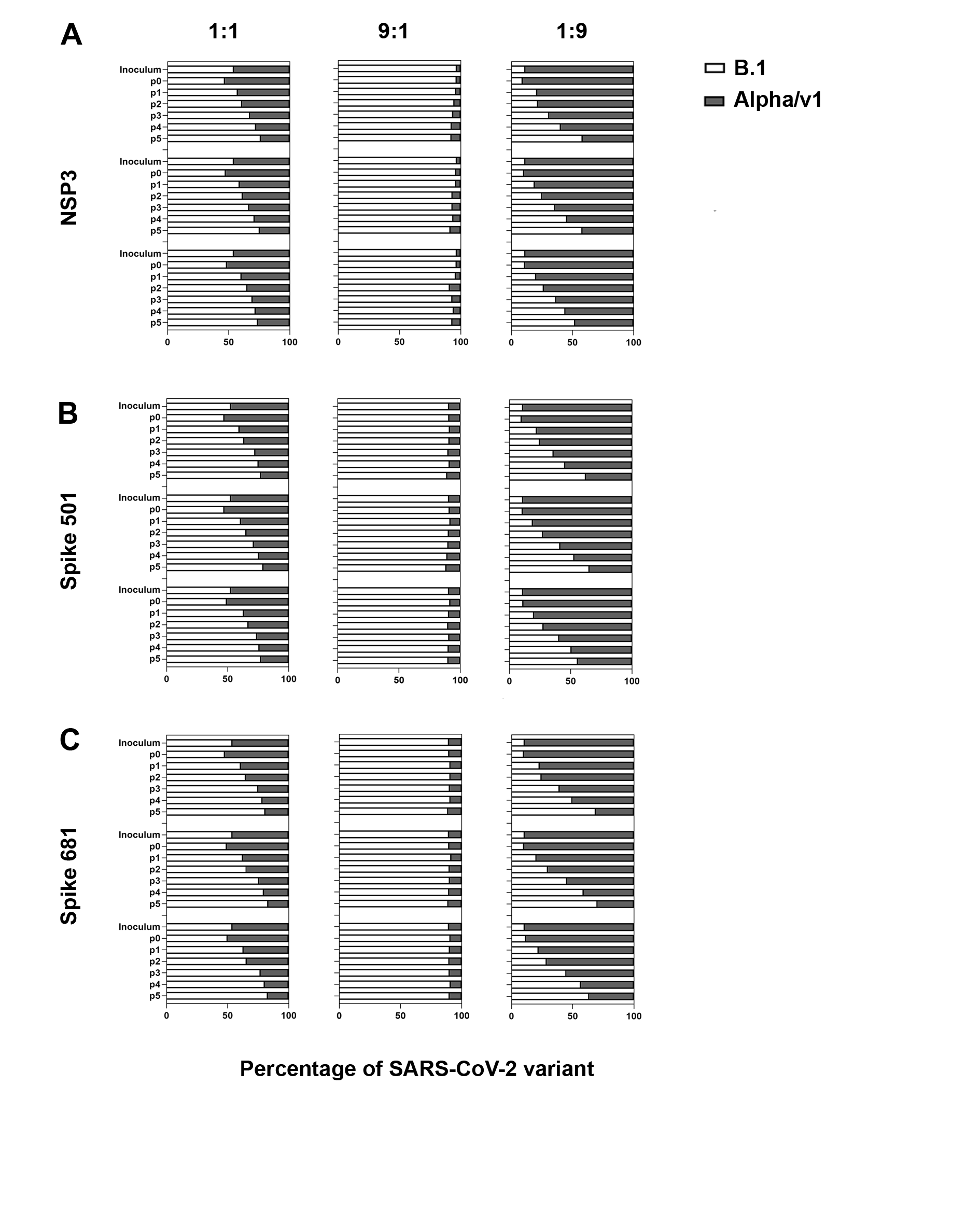

Supplement: S1 Fig — Calu-3 cells were infected with a mixture of B.1 and VOC Alpha at indicated ratios (B.1:VOC Alpha/v1 ratio of 1:1, 9:1, and 1:9) with a total infectious dose of 10,000 PFU (corresponding to an MOI of 0.04). After serial passaging, viral RNA from the supernatant was isolated, sequenced, and the relative proportion of B.1- and VOC Alpha-corresponding sequences, discriminated by mutations in NSP3 (A), Spike amino acid positions 501 (B) and 681 (C) was plotted. Data show individual values of triplicates of 1 experiment. MOI, multiplicity of infection; PFU, plaque-forming units; p0-p5, passage 0–passage 5; VOC, variant of concern. See S1 Data. (TIF) [file pbio.3001871.s001.tif]

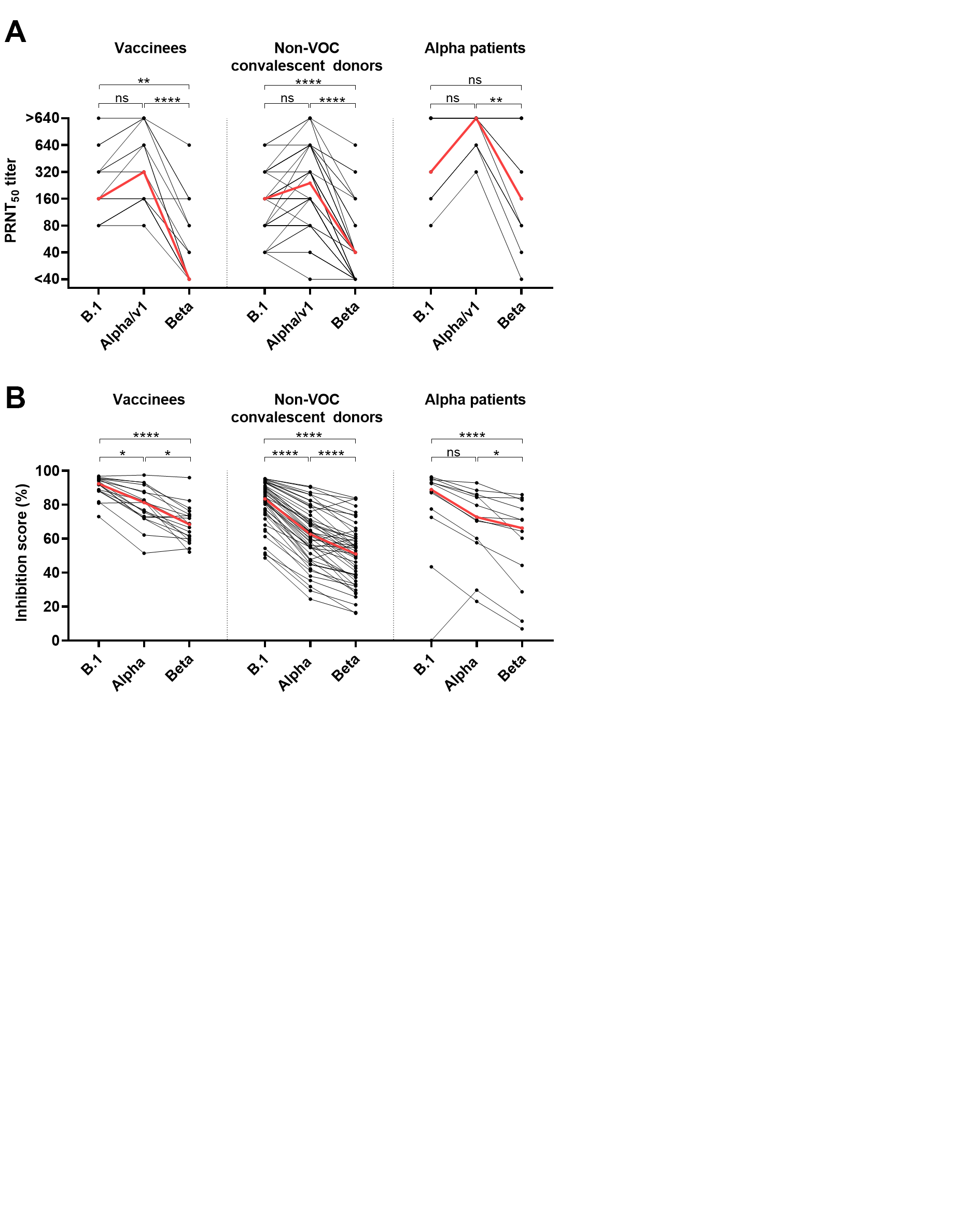

Supplement: S2 Fig — (A) Neutralizing titers against the indicated virus strains were determined in PRNTs. Red line indicates median titers per group. (B) Inhibition of ACE2/RBD interaction was measured using surrogate virus neutralization assays. Sera were tested using RBD proteins of B.1, VOC Alpha-, and Beta-VOC as indicated. Red lines indicate median values. The same set of samples was measured in (A) and (B), vaccinees n = 19, non-VOC convalescent donors n = 50, B1.1.7 patients n = 13. PRNT, plaque reduction neutralization test; VOC, variant of concern. See S1 Data. (TIF) [file pbio.3001871.s002.tif]

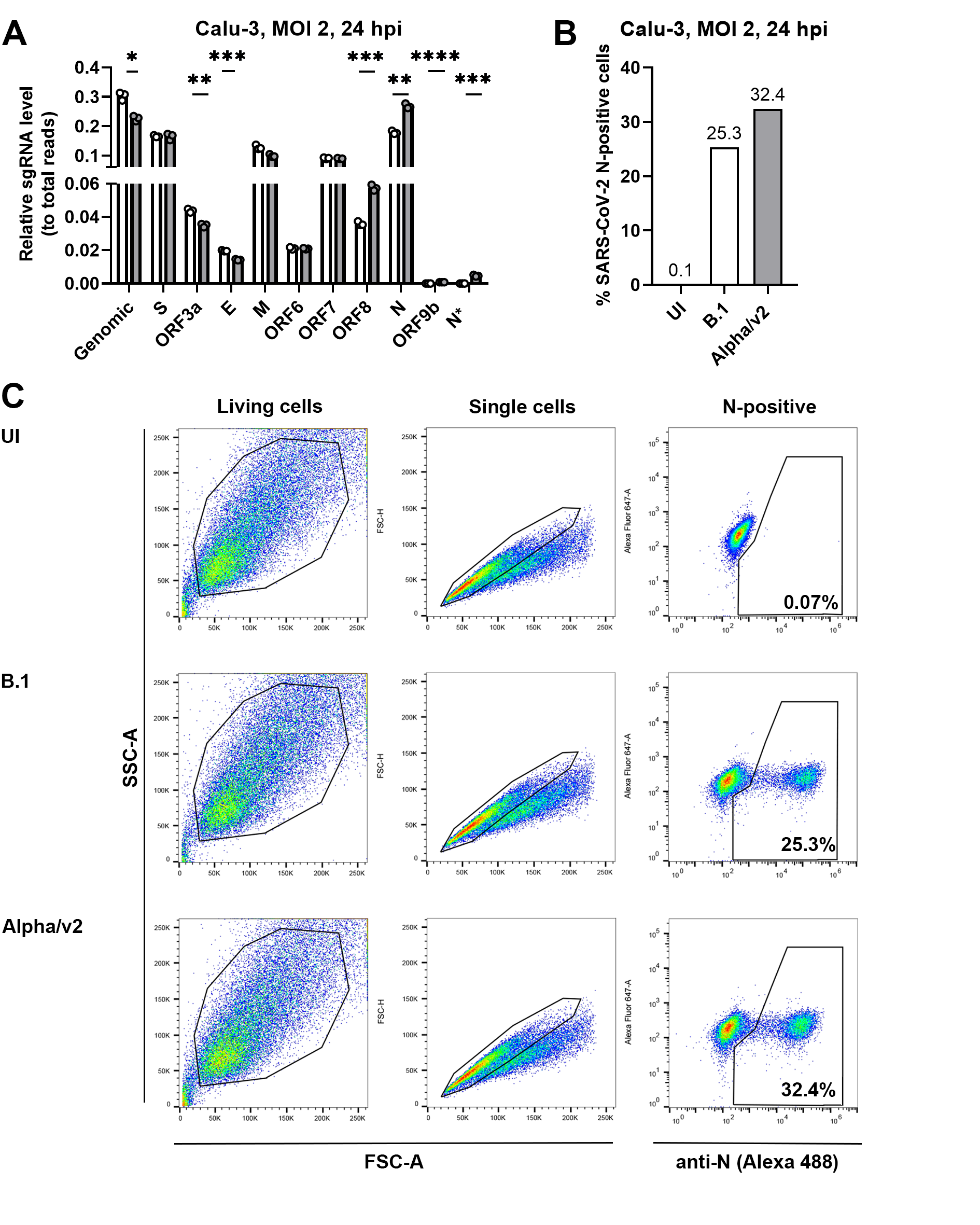

Supplement: S3 Fig — (A) RNA-seq analysis was conducted from total cell lysates that were obtained 24 hours postinfection to quantify sgRNA proportions in SARS-CoV-2-infected cells (MOI of 2). Canonical, as well as ORF9b and N* sgRNAs were quantified from the RNA-seq dataset. Data were normalized to total RNA reads. (B, C) Number of SARS-CoV-2 nucleocapsid (N)-positive Calu-3 cells was determined by flow cytometry. Calu-3 were left either UI or were infected with B.1 and VOC Alpha (MOI of 2) for 24 hours, permeabilized and immunostained with rabbit-anti-SARS-CoV-2 nucleocapsid antibody, followed by goat anti-rabbit Alexa 488 secondary antibody. (B) Percentage of SARS-CoV-2 N-positive cells. (C) Gating strategy of living-, single-, and N-positive cells is depicted for UI, B.1-, and VOC Alpha-infected cells. MOI, multiplicity of infection; SARS-CoV-2, Severe Acute Respiratory Syndrome Coronavirus 2; sgRNA, subgenomic RNA; UI, uninfected; VOC, variant of concern. See S1 Data. (TIF) [file pbio.3001871.s003.tif]

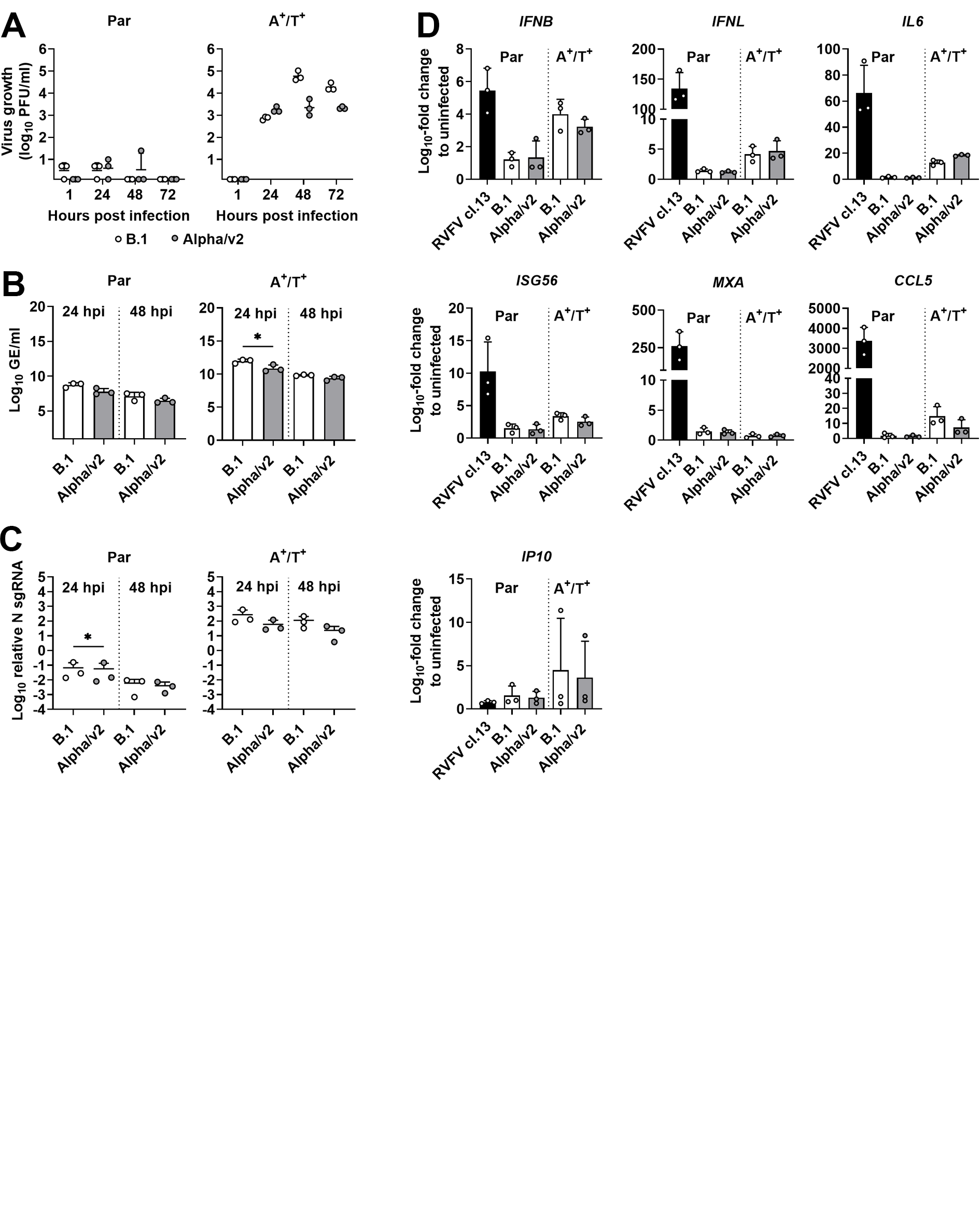

Supplement: S4 Fig — (A) Virus growth was quantified in parental and ACE2/TMPRSS2-expressing A549 cells infected at an MOI of 0.01. Supernatant collected at the respective time points was titrated by plaque assay on Vero E6 cells. Growth kinetic experiments were performed once in triplicates. (B) Expression of cell-associated envelope was determined in parental and ACE2/TMPRSS2-expressing A549 cells at 24 and 48 hours postinfection by Q-RT-PCR. (C) Expression of cell-associated sgN in Calu-3 cells at 24 and 48 hours postinfection was determined by Q-RT-PCR. TBP was used for normalization. (D) Expression of the indicated genes was determined by Q-RT-PCR. Shown is the mean fold change +/− SD of 3 biologically independent experiments that were each conducted in quadruplicates. RVFV cl.13, which is devoid of its IFN antagonist NSs, was included for the analysis of expression of IFNs and ISGs. A+/T+, ACE2/TMPRSS2-expressing A549 cells; GE, genome equivalents; par, parental; IFN, interferon; MOI, multiplicity of infection; Q-RT-PCR, quantitative real-time PCR; RVFV cl.13, Rift Valley Fever Virus clone 13; sgN, subgenomic nucleocapsid; TBP, TATA-binding protein. See S1 Data. (TIF) [file pbio.3001871.s004.tif]

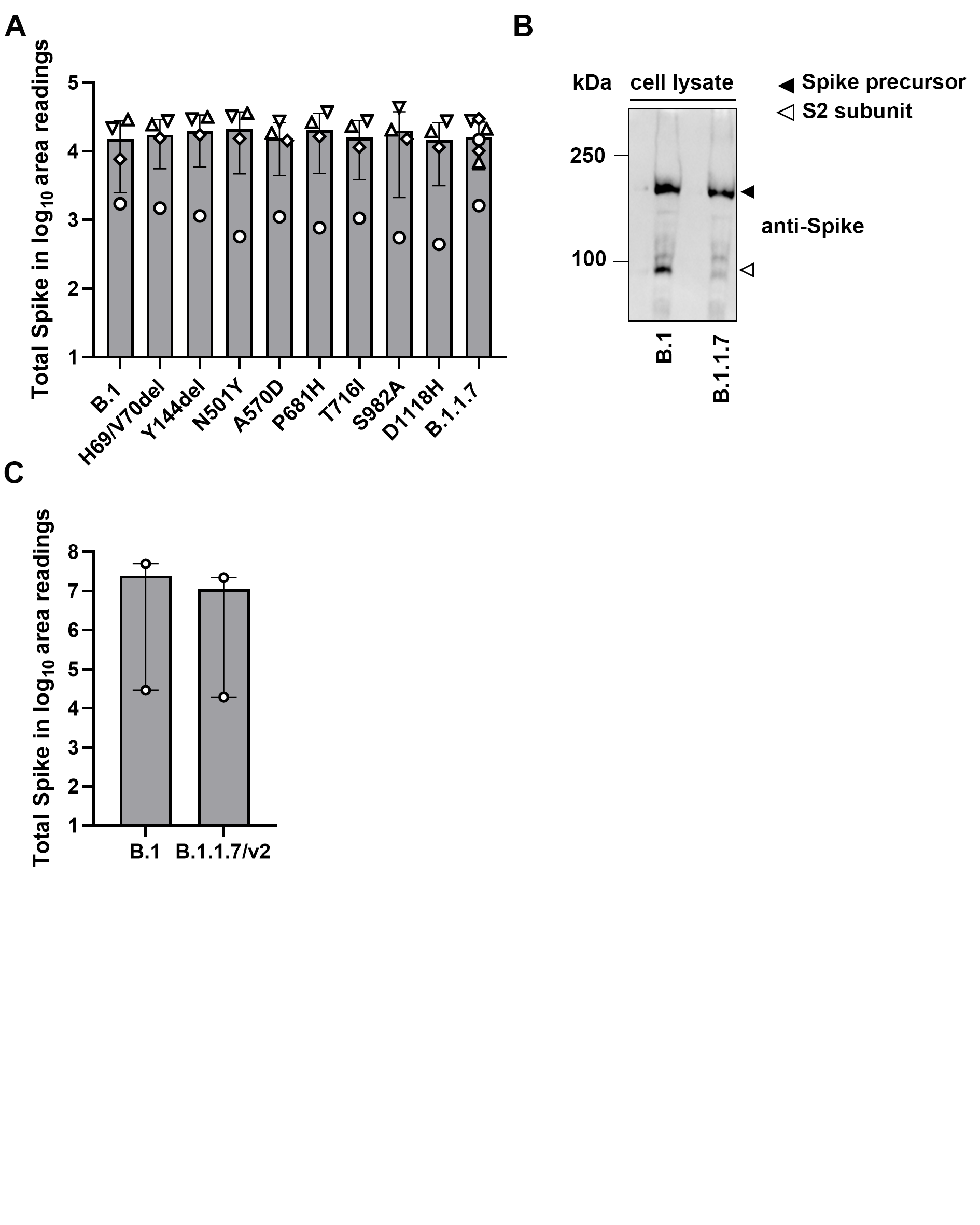

Supplement: S5 Fig — (A) Expression of total spike in HEK 293T cells. Symbols represent independently performed experiments. (B) Vero E6 cells were infected with SARS-CoV-2 (MOI 5). Cells and virus-containing supernatants were harvested at 48 hours postinfection and processed for detection of spike by immunoblotting. (C) Expression of total spike in Vero E6 cells was quantified by the use of ImageJ 1.48v. MOI, multiplicity of infection; SARS-CoV-2, Severe Acute Respiratory Syndrome Coronavirus 2; VOC, variant of concern. See S1 Data. (TIF) [file pbio.3001871.s005.tif]

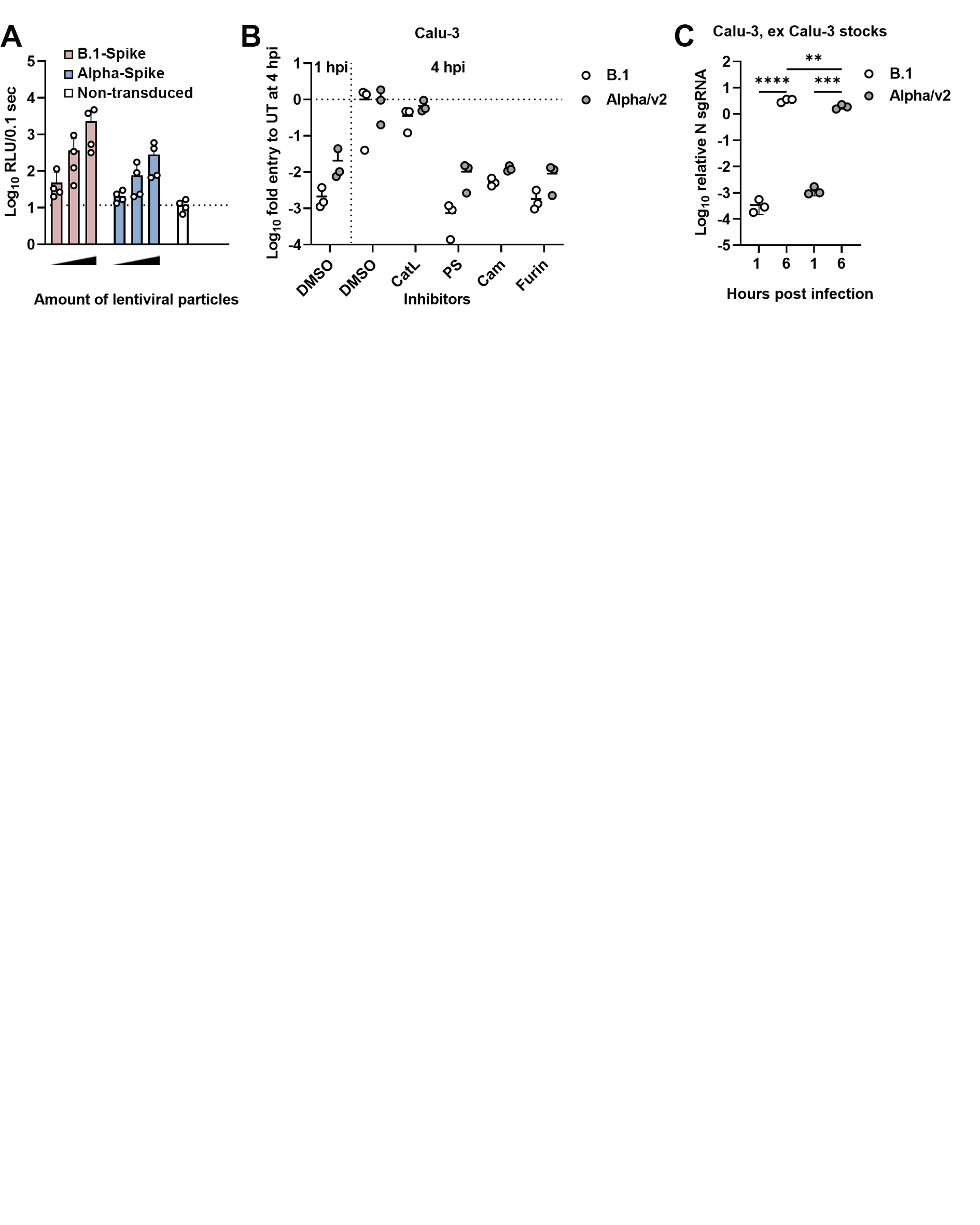

Supplement: S6 Fig — (A) Calu-3 cells were transduced for 72 hours with increasing amounts of lentiviral particles (0.1 μl, 1 μl, and 10 μl) pseudotyped with either B.1 or VOC Alpha spike proteins. Pseudotype entry was analyzed luminometrically in cell lysates. (B) Calu-3 cells were pretreated with 25 μM MDL28170 (Cathepsin L inhibitor), 25 μM pitstop II (clathrin inhibitor), 100 μM Camostat (TMPRSS2 inhibitor), or 15 μM CMK (furin inhibitor), infected and entry efficiency was determined by sgN Q-RT-PCR. (C) Calu-3 cells were infected with Calu-3-derived virus stocks. Entry efficiency was determined by sgN-specific Q-RT-PCR from cell lysates at 4 hours postinfection. Cam, Camostat mesylate; CatL, Cathepsin L; DMSO, Dimethylsulfoxid; PS: PitStop; Q-RT-PCR, quantitative real-time PCR; sgN, subgenomic nucleocapsid; TMPRSS2, transmembrane protease serine subtype 2; VOC, variant of concern. See S1 Data. (TIF) [file pbio.3001871.s006.tif]

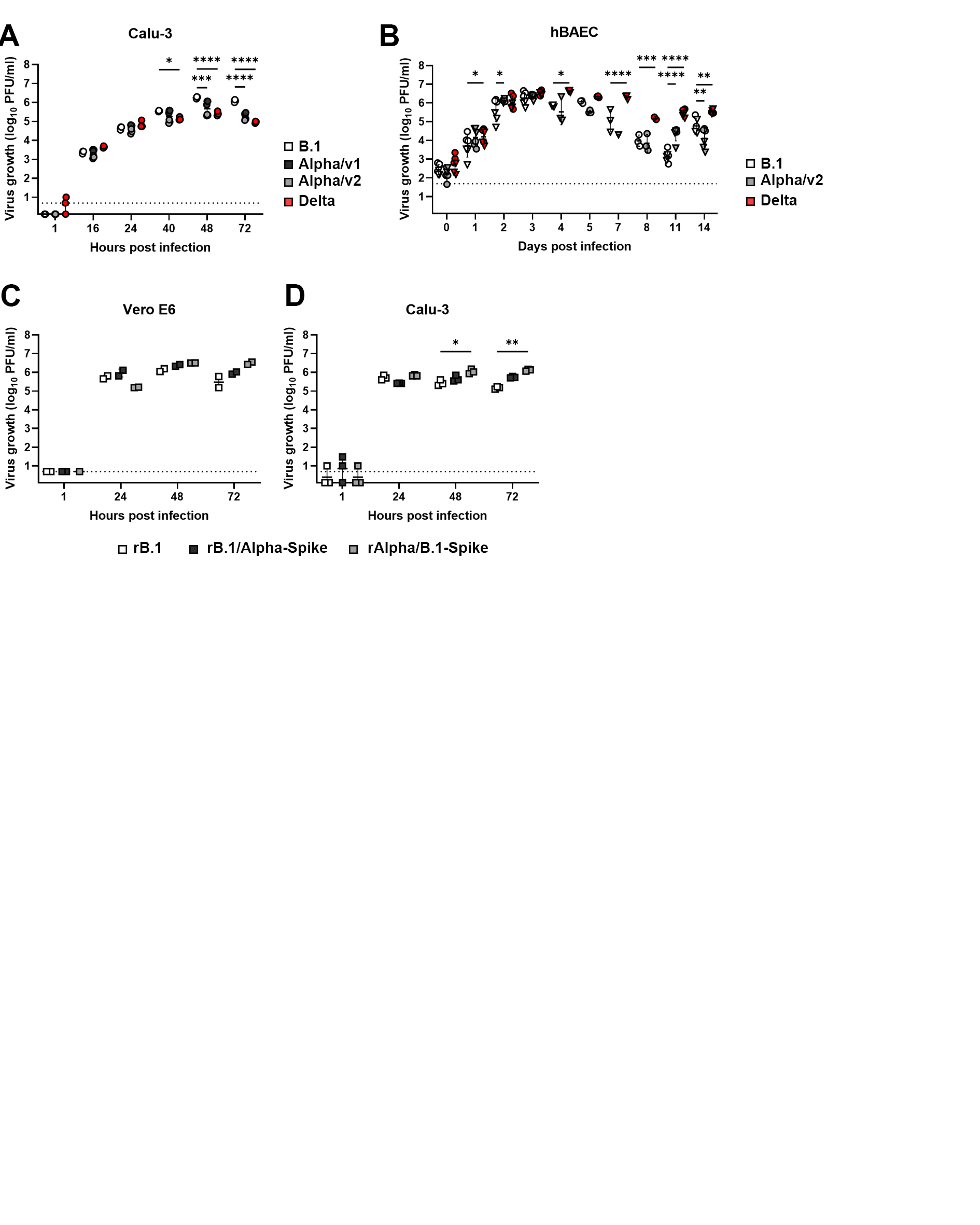

Supplement: S7 Fig — (A) Virus growth of B.1, VOC Alpha/v2, and Delta isolates (MOI 0.01) was quantified in Calu-3 cells. (B) Virus growth of B.1, VOC Alpha/v2, and Delta isolates (MOI 0.01) was quantified in hBAECs. (C, D) Vero E6 (C) and Calu-3 (D) cells were infected with rB.1, rB.1/Alpha spike, and rAlpha/B.1 spike (MOI 0.01) and supernatant was titrated on Vero E6 cells. The growth experiment in Vero E6 cells was performed once in duplicates. Growth experiments in Calu-3 cells was performed once in triplicates. Dashed horizontal lines indicate the lower limit of detection of the plaque assay. hBAEC, human bronchial airway epithelial cell; MOI, multiplicity of infection; VOC, variant of concern. See S1 Data. (TIF) [file pbio.3001871.s007.tif]

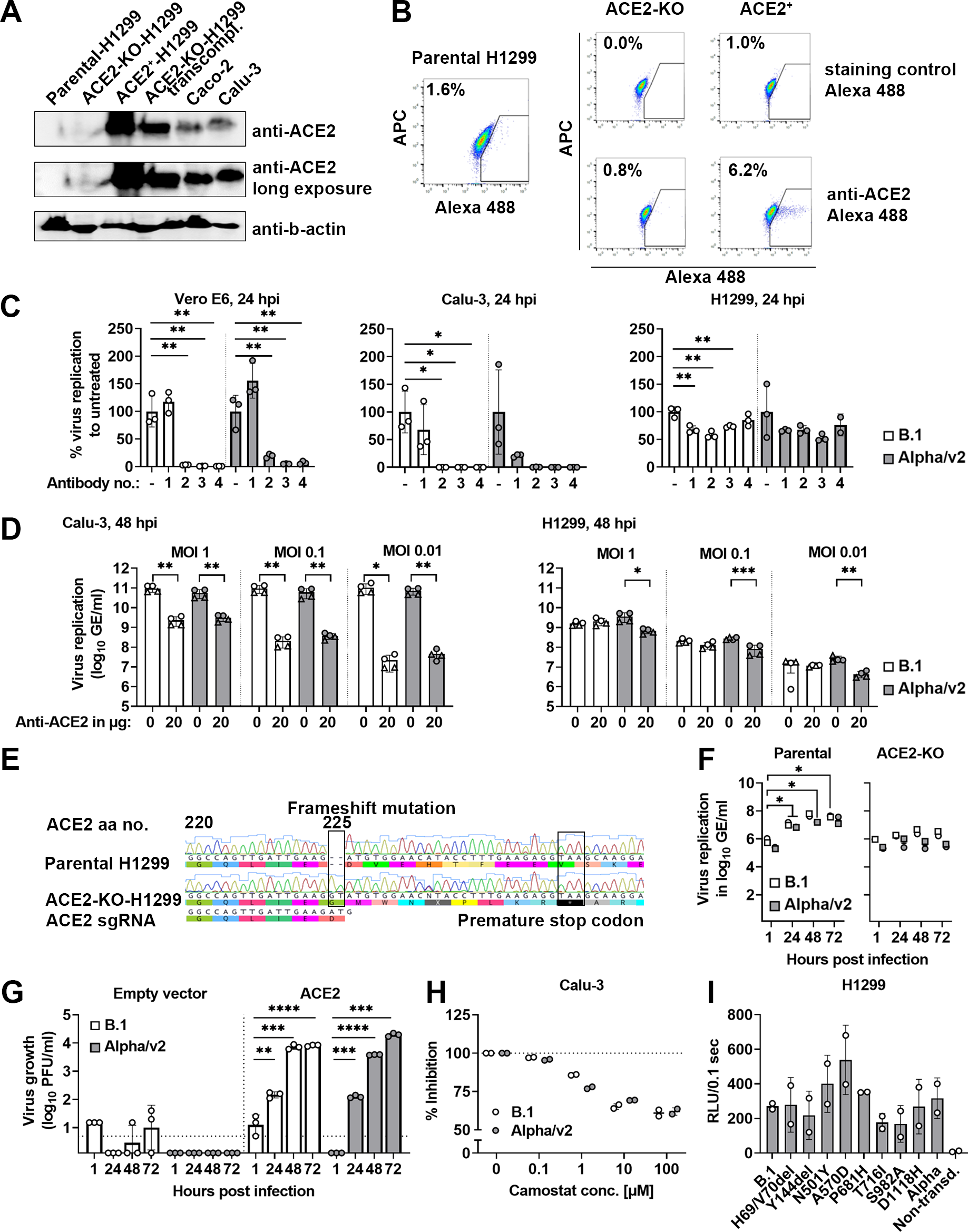

Supplement: S8 Fig — (A) ACE2 expression levels of the indicated NCI-H1299 cells, Caco-2 and Calu-3 were analyzed by immunoblotting. Beta-actin was used as a loading control. (B) ACE2 expression levels of the indicated NCI-H1299 cells were determined by flow cytometry. % numbers of ACE2-positive NCI-H1299 cells are indicated in the respective dot plots. (C) Vero E6 (left), Calu-3 (middle), and NCI-H1299 (right) cells were pretreated with 4 different anti-ACE2 antibodies (each applied at final concentration of 20 μg/ml) for 1 hour prior to infection with B.1- and VOC Alpha isolates (MOI of 0.01). At 24 hours postinfection, viral replication was quantified from the supernatant by the use of E-gene assay. Replication was normalized to the respective untreated cells. Results from 1 experiment, conducted in triplicates, are shown. (D) Calu-3 (left) and NCI-H1299 (right) cells were pretreated with 20 μg/ml anti-ACE2 antibody for 1 hour prior to infection with B.1 and VOC Alpha/v2 isolates (MOI of 0.01). At 48 hours postinfection, viral replication was quantified from the supernatant by the use of E-gene assay. Results from 2 independently performed experiments, each conducted in triplicates, are shown. (E) Sequencing chromatogram covering the ACE2 gene locus of NCI-H1299 and ACE2-KO-H1299 cells, expressing ACE2 protein from aa no. 220 ff. Knock-out of ACE2 in ACE2-KO-H1299 cells was induced by CRISPR/Cas9 technology by the use of a synthetic guide RNA (sgRNA) targeting the genomic ACE2 region as indicated in the chromatogram. A frameshift mutation was generated through the insertion of 2 nucleotides, which leads to the expression of a truncated ACE2 protein (from aa 225 ff) due to a premature stop codon in the ACE2-encoding reading frame (black boxes). (F) Virus replication of B.1 and VOC Alpha was investigated on parental-NCI-H1299 (left) and respective ACE2-KO (right) cells. Cells were infected (MOI 0.01) and genome equivalents were determined by E gene assay from the supernatants at indica [file pbio.3001871.s008.tif]

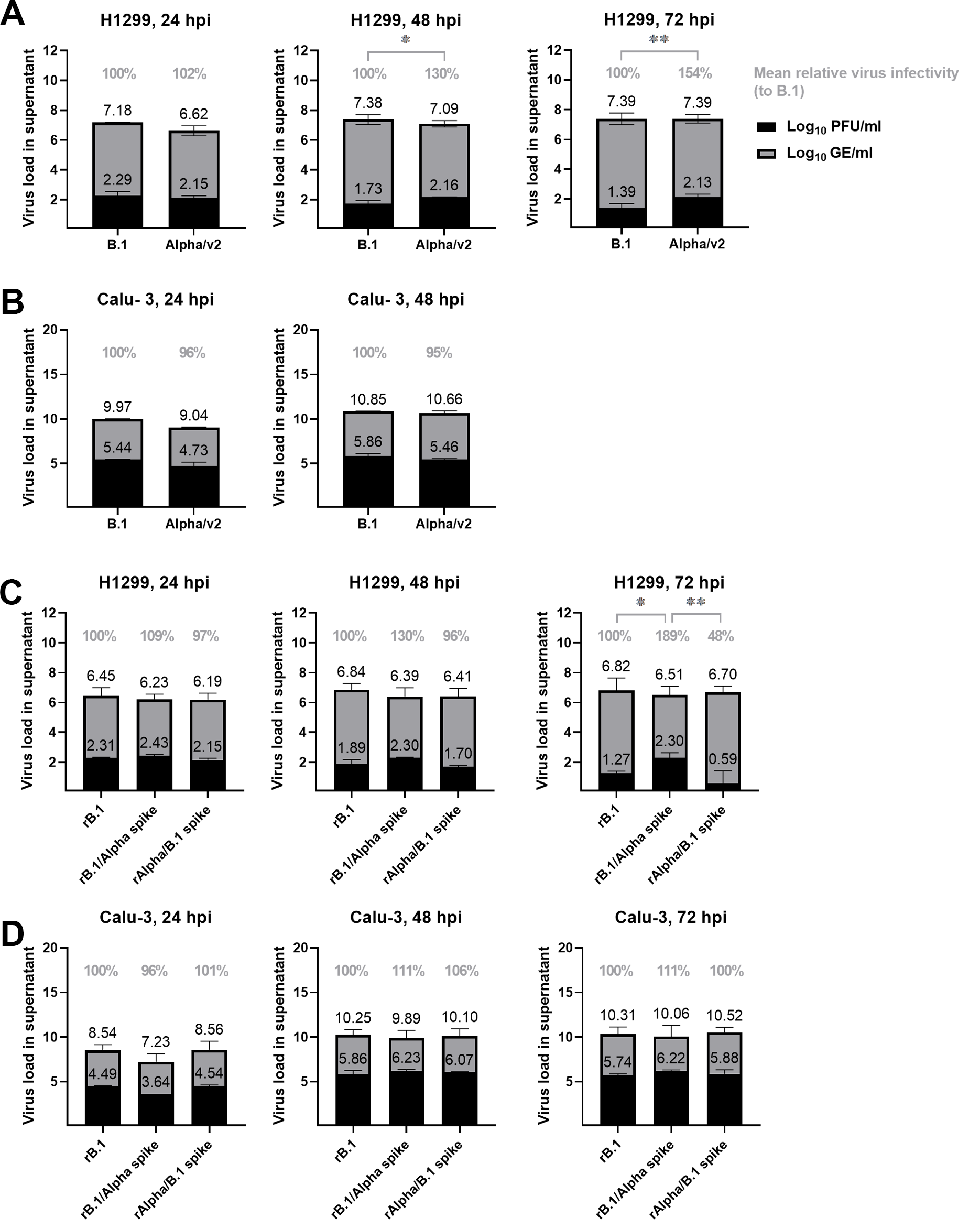

Supplement: S9 Fig — NCI-H1299 and Calu-3 cells were infected (MOI of 0.01) with either B.1 and VOC Alpha isolates (A, B) or rB.1, rB.1/Alpha spike, and rAlpha/B.1 spike (C, D). Supernatants were collected at 24, 48, and 72 hours postinfection and titrated by plaque assay on Vero E6 cells to determine PFU/ml. Genome equivalents (GE/ml) were determined by E gene assay. Two independent experiments, each conducted in triplicates, were performed. Gray numbers indicate mean relative virus infectivity relative to B.1 or rB.1, respectively, of the independent experiments. Bars represent arithmetic means of independent experiments. Graphs in (A, B) depicting relative particle infectivity at 48 hours postinfection and graphs in (C, D) depicting relative particle infectivity at 72 hours postinfection are additionally shown in the main figures. GE, genome equivalents; MOI, multiplicity of infection; PFU, plaque-forming units; VOC, variant of concern. See S1 Data. (TIF) [file pbio.3001871.s009.tif]

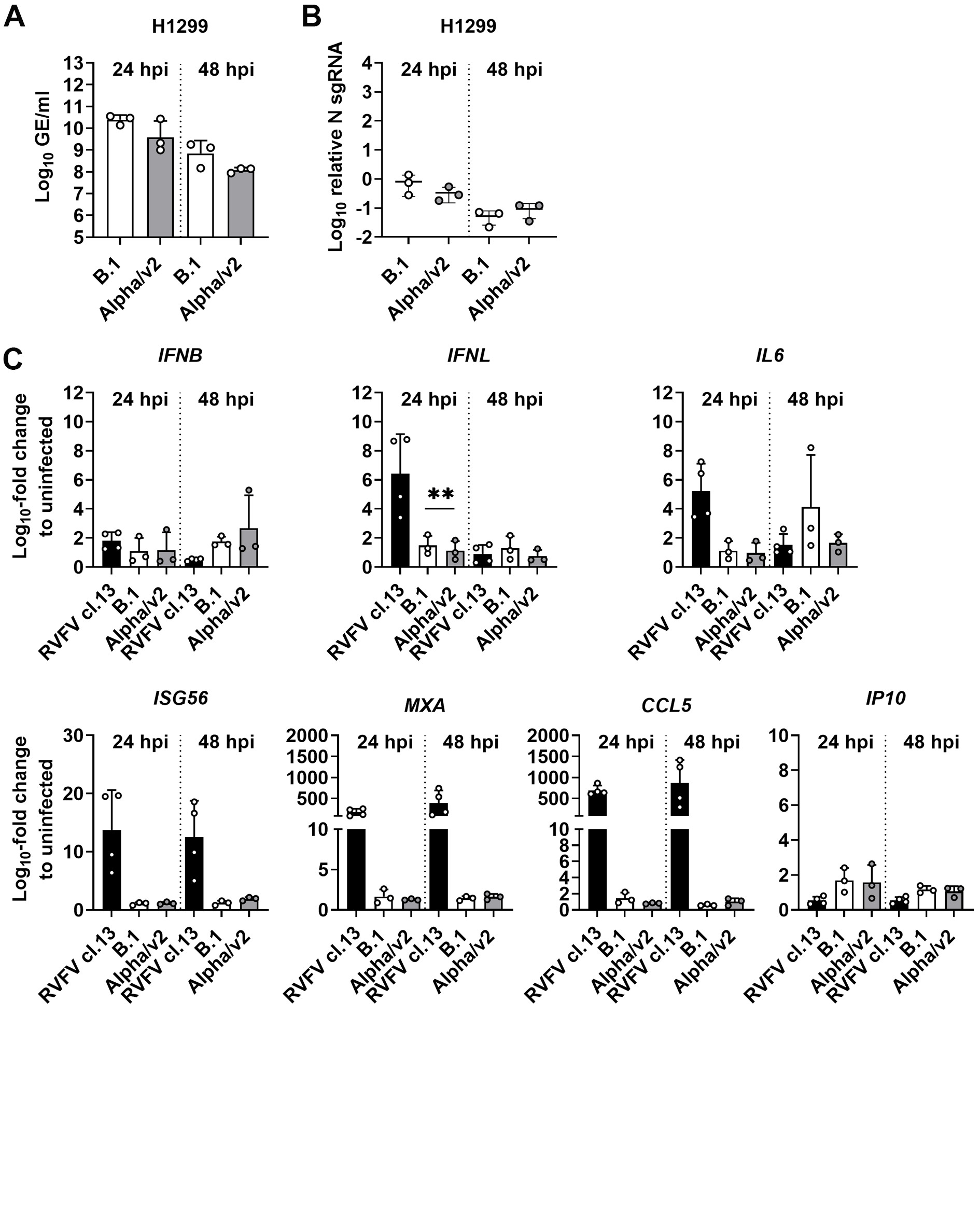

Supplement: S10 Fig — NCI-H1299 cells were infected with B.1 or VOC Alpha (MOI of 2), and viral replication, viral transcription, and expression of innate immune genes were determined by Q-RT-PCR from cell lysates at 24 and 48 hours postinfection. (A) Expression of cell-associated envelope. (B) Expression of cell-associated sgN RNA. TBP was used for normalization. (C) Expression of the indicated genes was determined by specific Q-RT-PCR. TBP was used for normalization. Shown is the mean fold change +/− SD of 3 biologically independent experiments that were each conducted in quadruples. RVFV cl.13, which is devoid of its IFN antagonist NSs, was included for the expression of IFNs, ISGs, and pro-inflammatory cytokines. GE, genome equivalents; IFN, interferon; ISG, IFN-stimulated gene; Q-RT-PCR, quantitative real-time PCR; RVFV cl.13, Rift Valley Fever Virus clone 13; sgN, subgenomic nucleocapsid; TBP, TATA-binding protein. See S1 Data. (TIF) [file pbio.3001871.s010.tif]

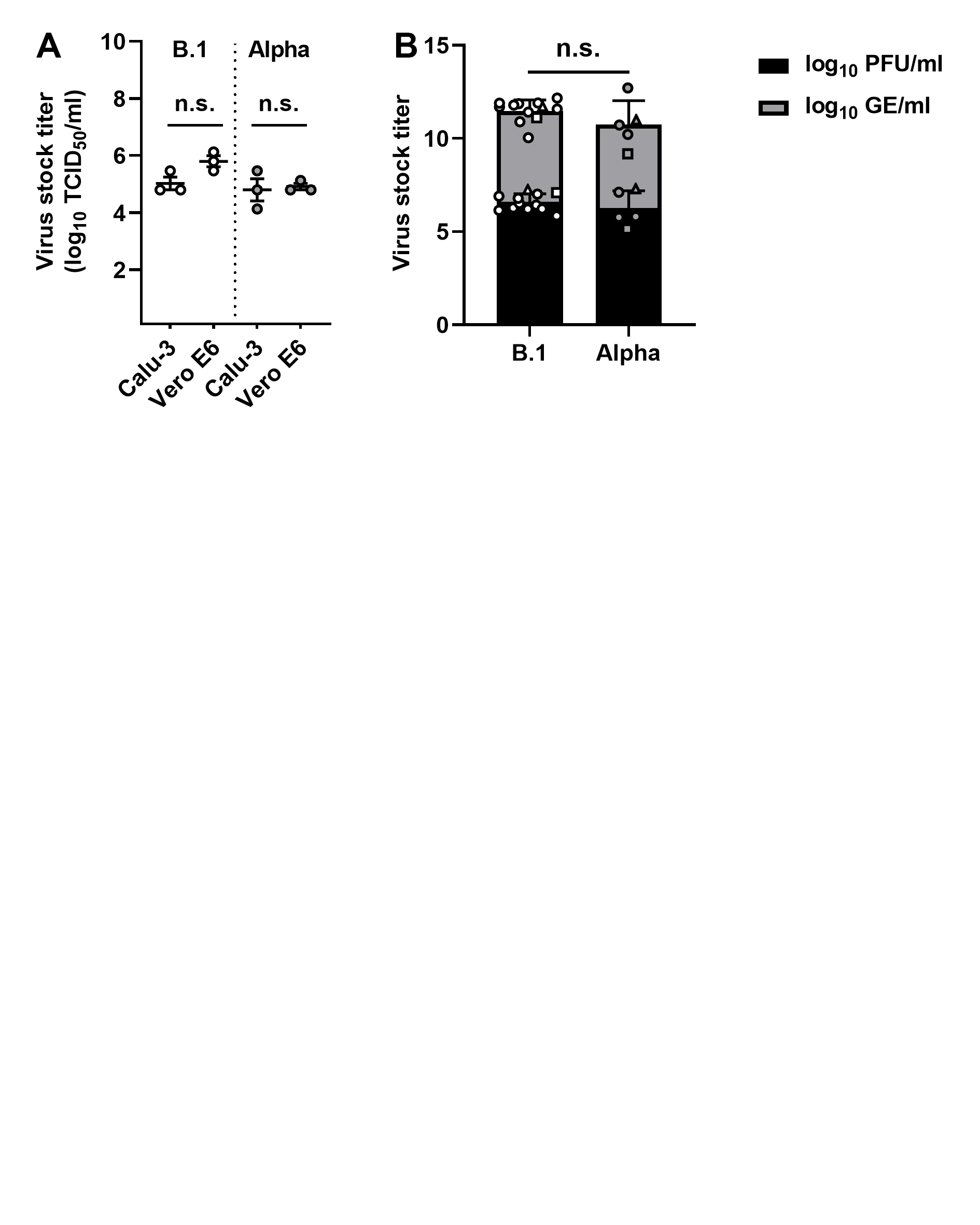

Supplement: S11 Fig — (A) No significant differences in the titers were observed when titers of B.1 and VOC Alpha SARS-CoV-2 stocks were compared on Calu-3 versus Vero E6 using TCID50 titration method. Although plaque morphology of B.1- and VOC Alpha-infected Vero E6 cells differ, Vero E6 cells are suitable to determine titers by plaque titration assay. N = 3 biologically independent experiments each conducted in triplicates. (B) Overview on virus infectivity and viral RNA concentrations, determined by plaque assay (log10 PFU/ml) and E gene assay (log10 GE/ml), of all virus stocks is shown. Direct comparison of all B.1 and VOC Alpha stocks. Statistical analysis was conducted between both viruses for genomic E gene and infectious titers, respectively. Stocks applied in gene expression analysis (triangle) and growth kinetics (square) are highlighted by symbols. GE, genome equivalents; n.s., not significant; PFU, plaque-forming units; Q-RT-PCR, quantitative real-time PCR; SARS-CoV-2, Severe Acute Respiratory Syndrome Coronavirus 2; VOC, variant of concern. See S1 Data. (TIF) [file pbio.3001871.s011.tif]
