# Supplementary material for: SARS-CoV-2 variant Alpha has a spike-dependent replication advantage over the ancestral B.1 strain in human cells with low ACE2 expression
Source: PLoS Biol. 2022 Nov 16;20(11):e3001871. doi: 10.1371/journal.pbio.3001871 (PMC9710838; doi:10.1371/journal.pbio.3001871)
Supplement: S1 Raw Images — (PDF) [file pbio.3001871.s018.pdf]

# Figure 5A

Replicate 1:

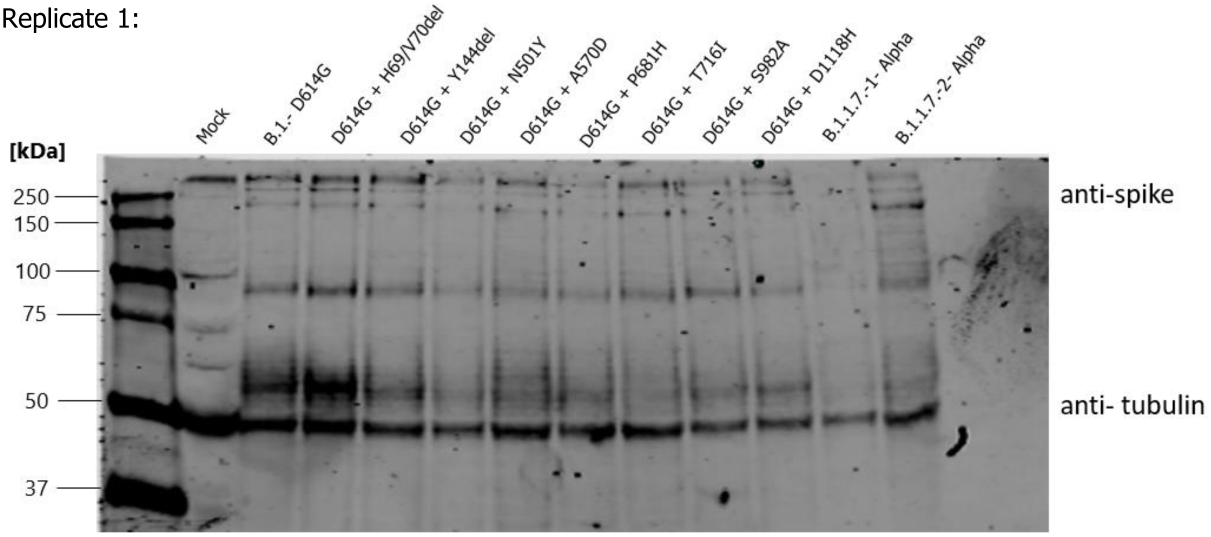


Replicate 2:

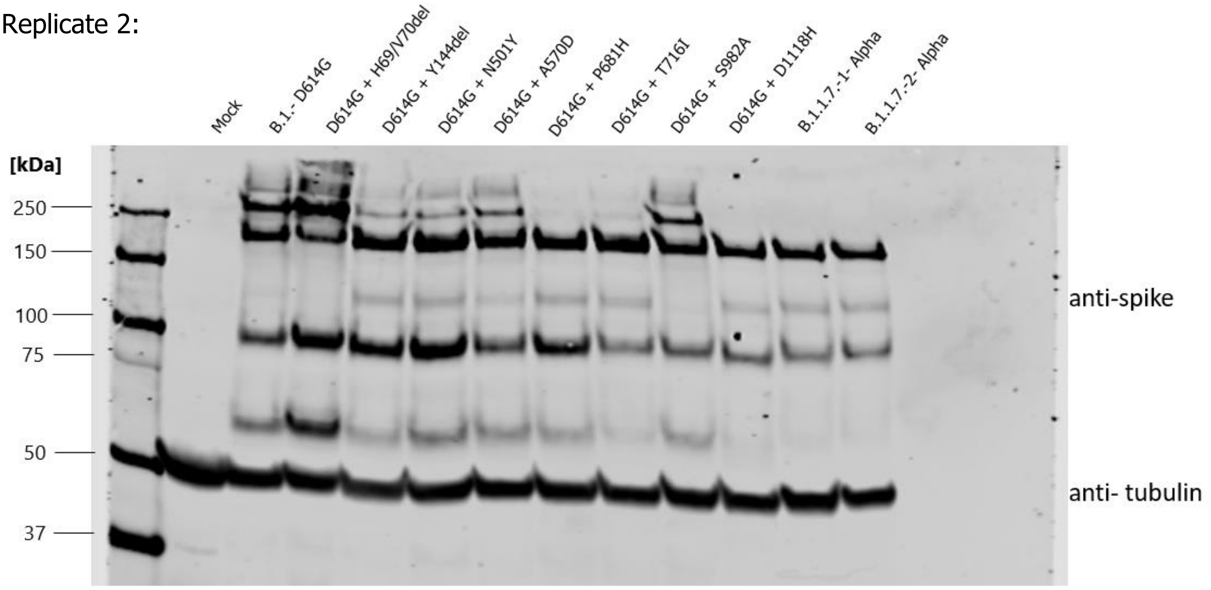


Replicate 3:

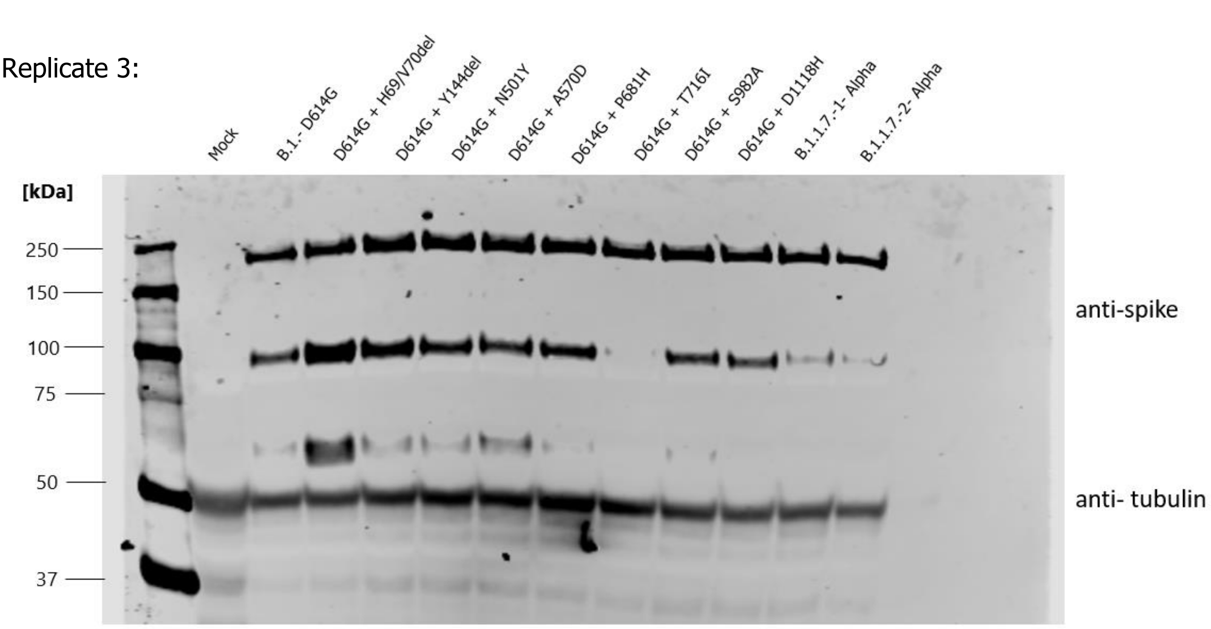

# Figure 5A

Replicate 4:

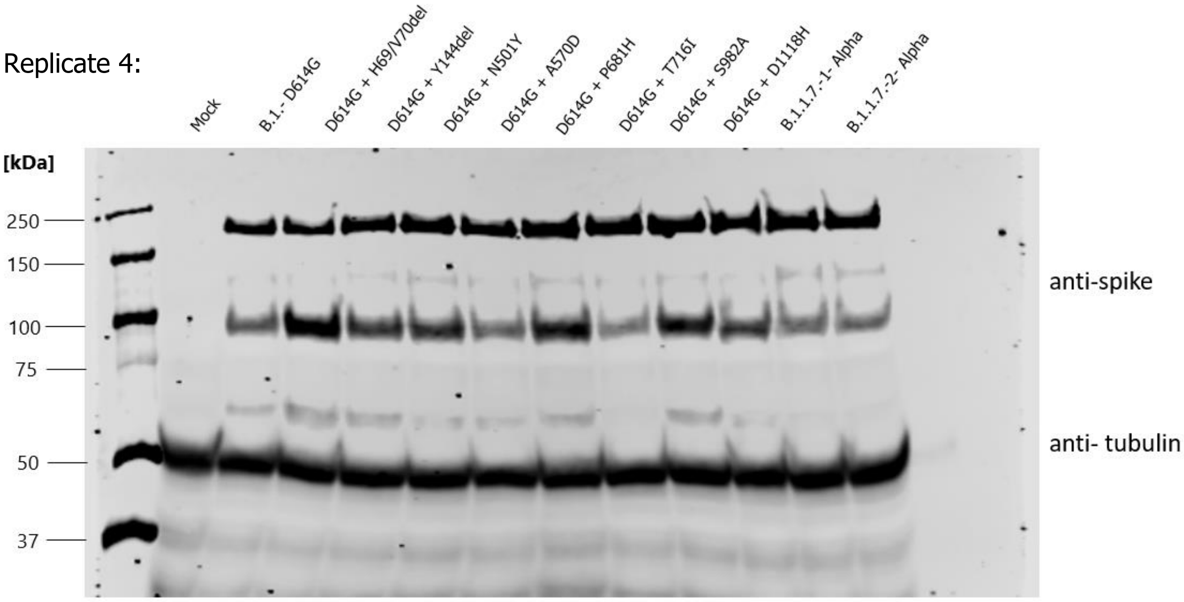


Replicate 5:

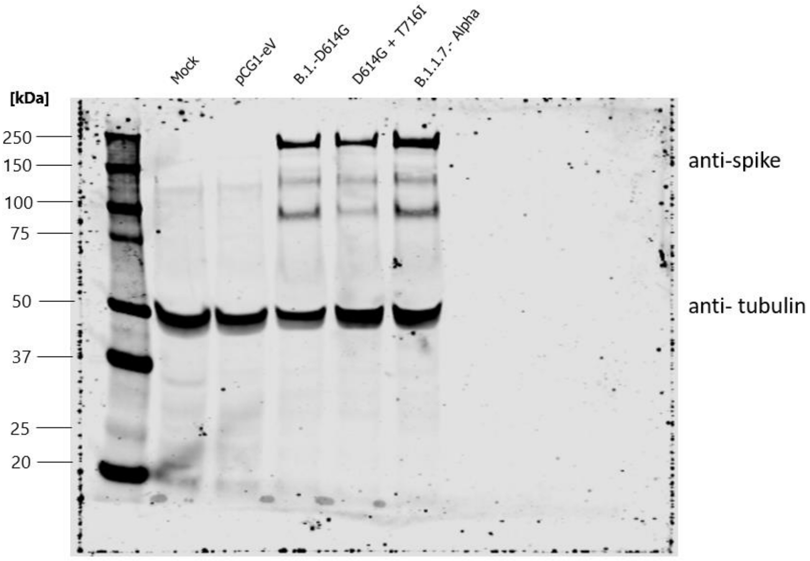


Replicate 6:

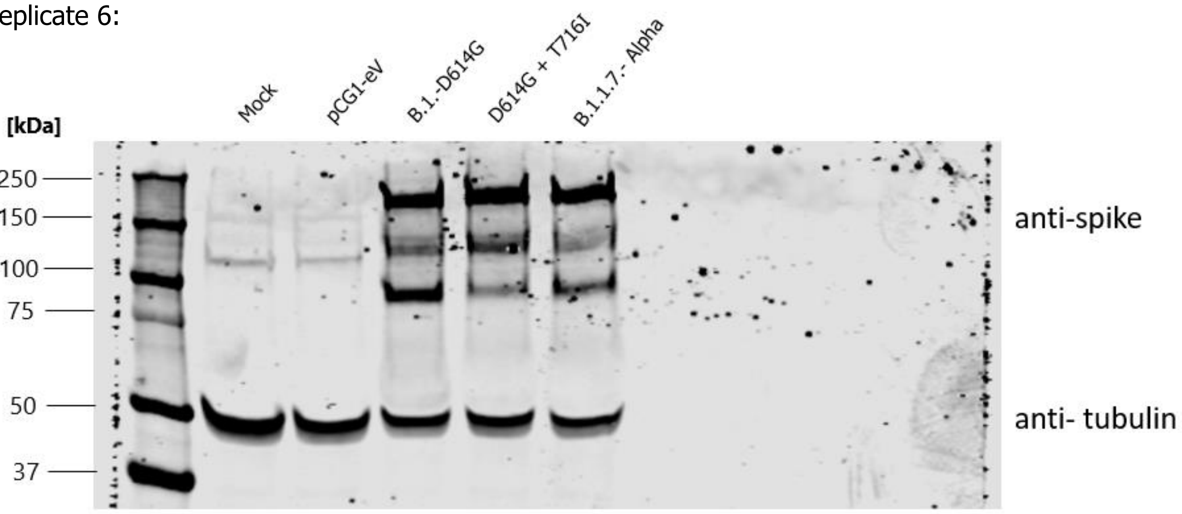

# Figure 5A

Replicate 7:

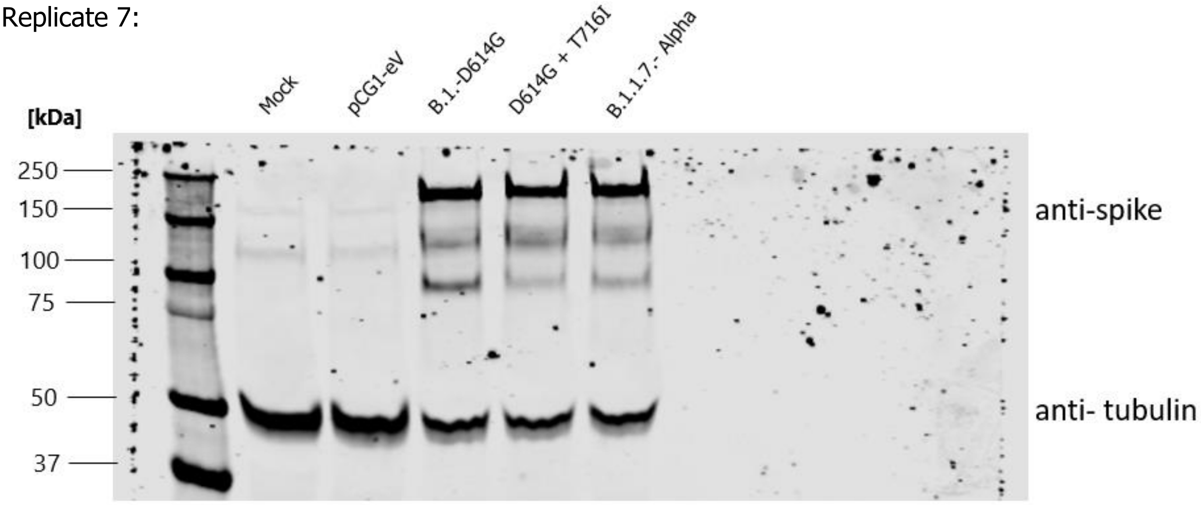

# Figure 5B

Replicate 1:

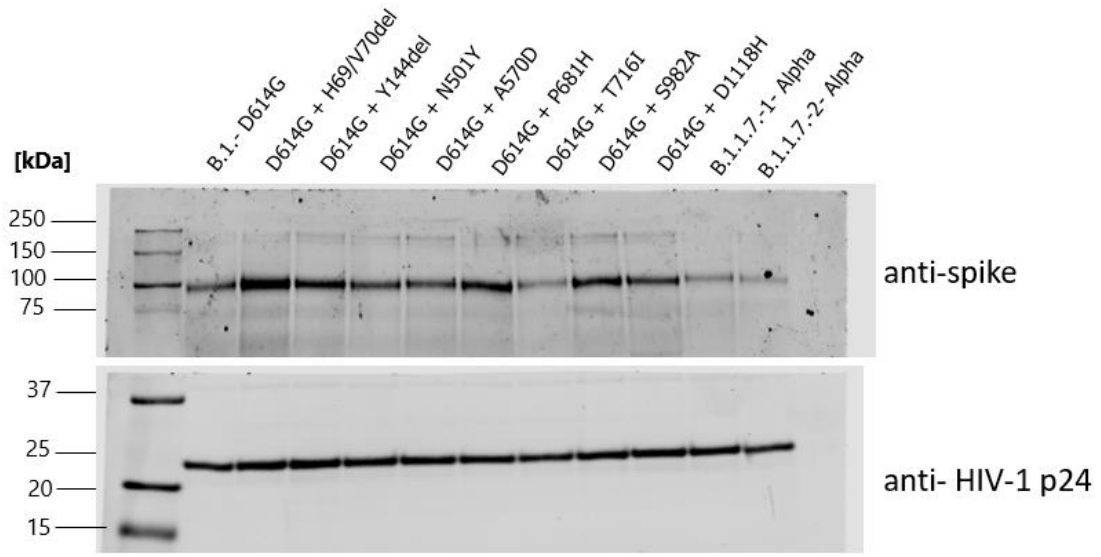


Replicate 2:

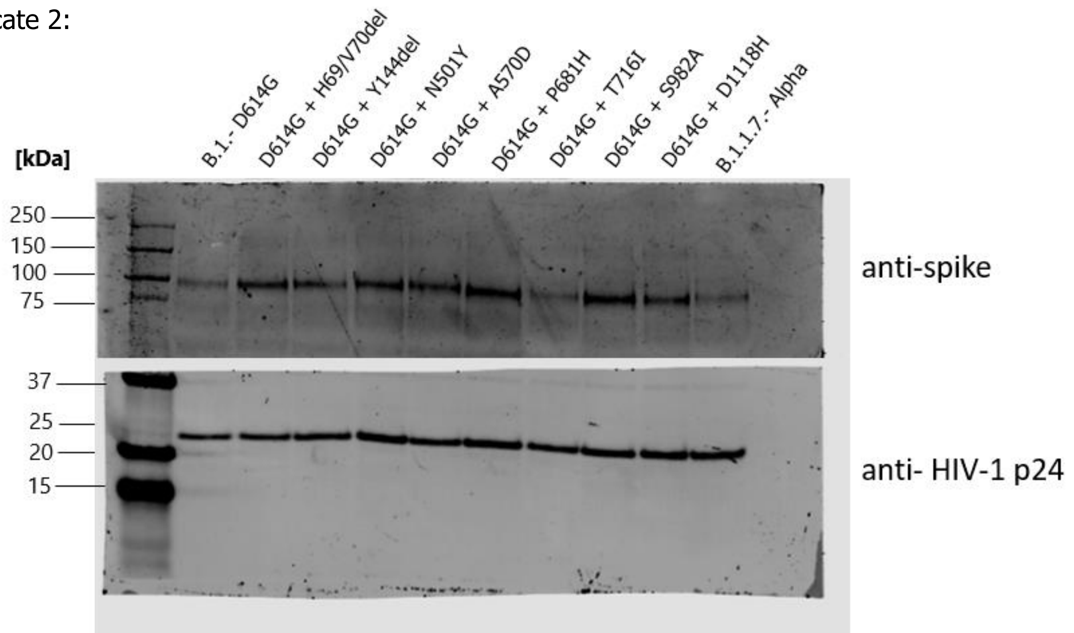

## Figure 5B

Replicate 3:

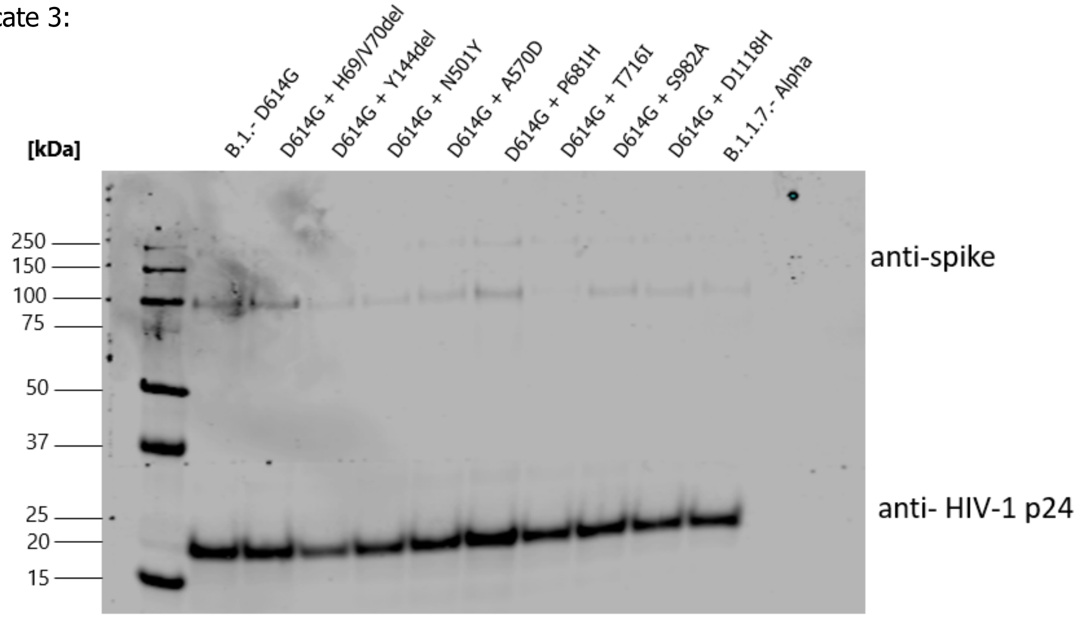


Replicate 4:

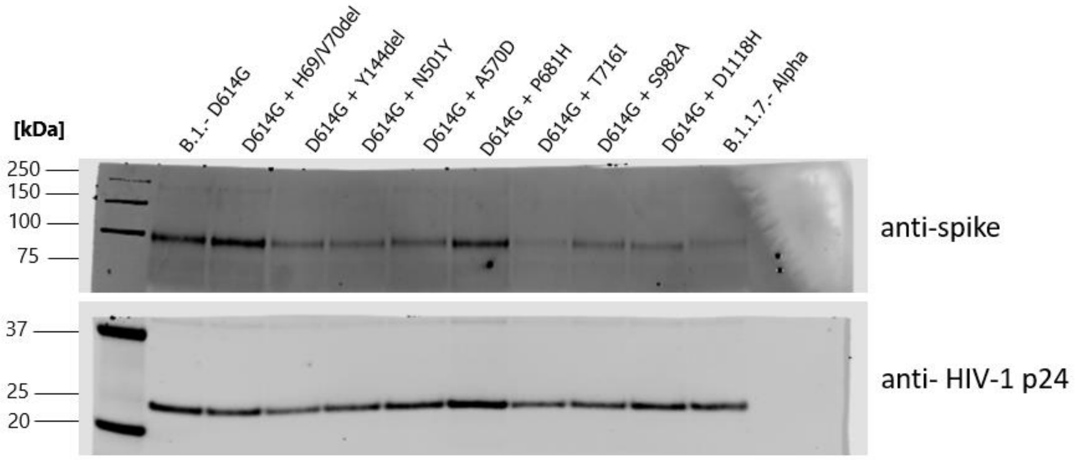


Replicate 5-7:

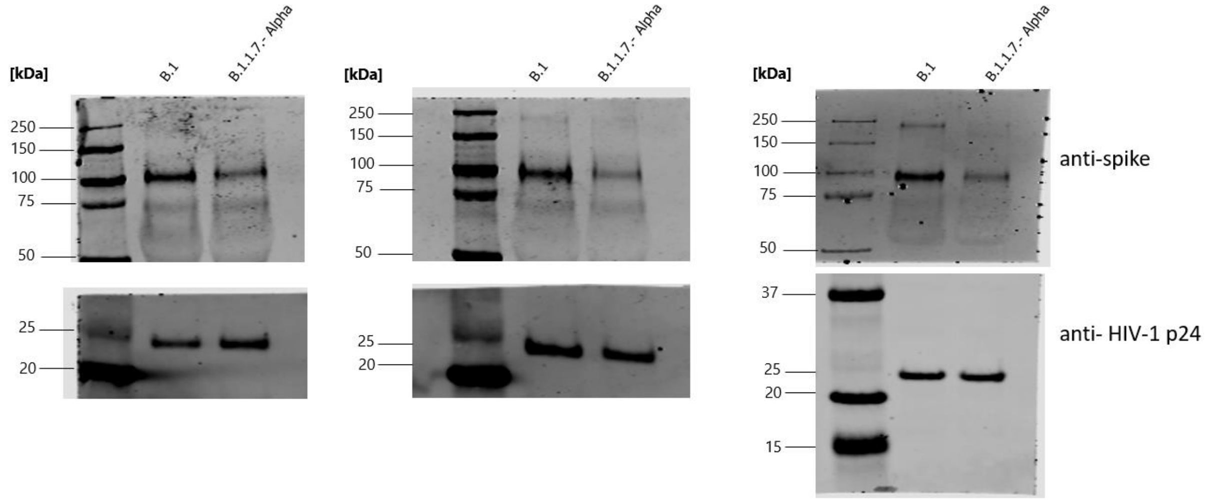

## Figure 5C

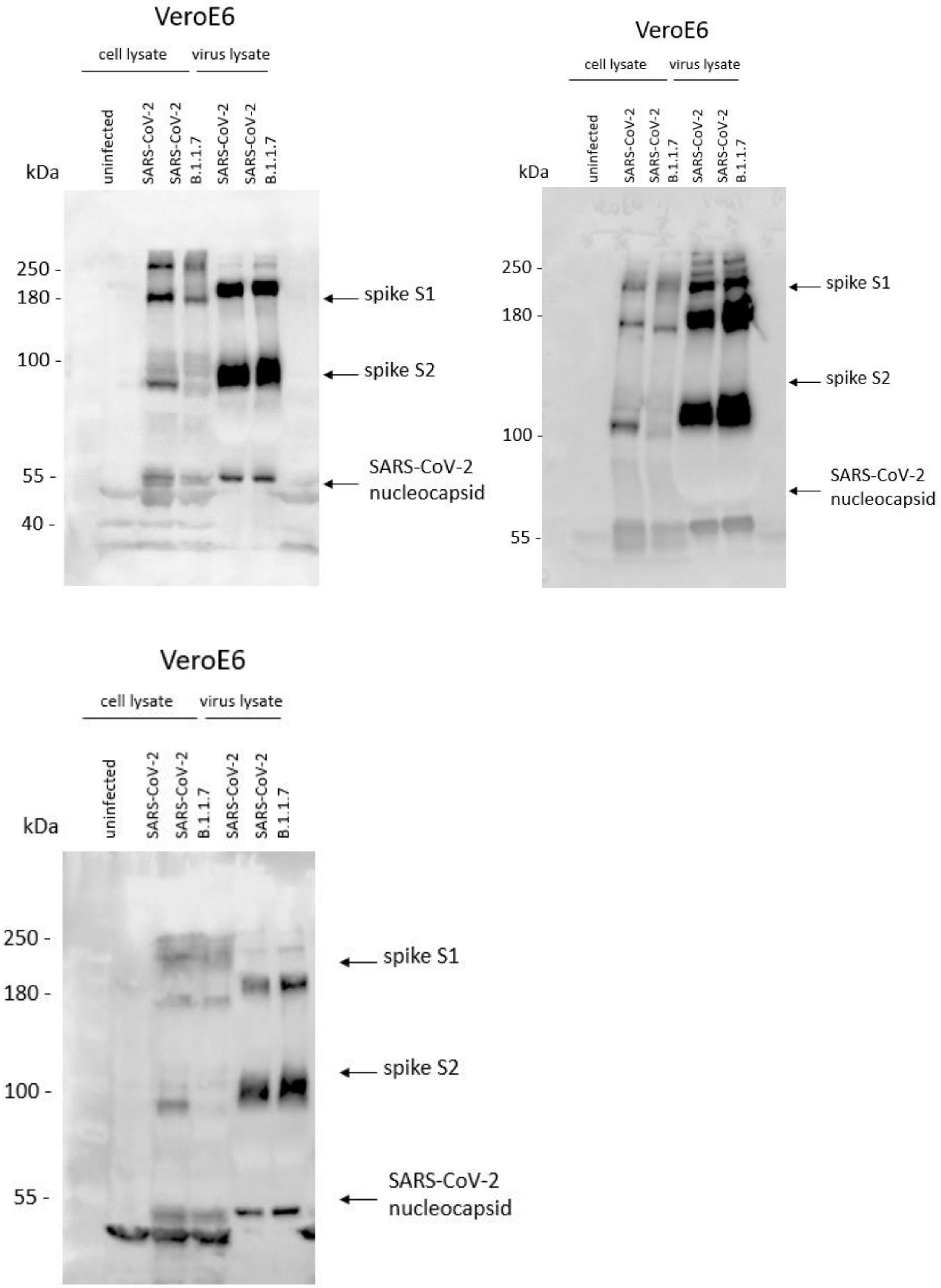

VeroE6
cell lysate
virus lysate
uninfected
SARS-CoV-2
SARS-CoV-2 B.1.1.7
SARS-CoV-2
SARS-CoV-2 B.1.1.7
kDa
250 -
180 -
100 -
55 -
40 -
spike S1
spike S2
SARS-CoV-2 nucleocapsid
VeroE6
cell lysate
virus lysate
uninfected
SARS-CoV-2
SARS-CoV-2 B.1.1.7
SARS-CoV-2
SARS-CoV-2 B.1.1.7
kDa
250 -
180 -
100 -
55 -
spike S1
spike S2
SARS-CoV-2 nucleocapsid
VeroE6
cell lysate
virus lysate
uninfected
SARS-CoV-2
SARS-CoV-2 B.1.1.7
SARS-CoV-2
SARS-CoV-2 B.1.1.7
kDa
250 -
180 -
100 -
55 -
spike S1
spike S2
SARS-CoV-2 nucleocapsid

**Figure 7C**

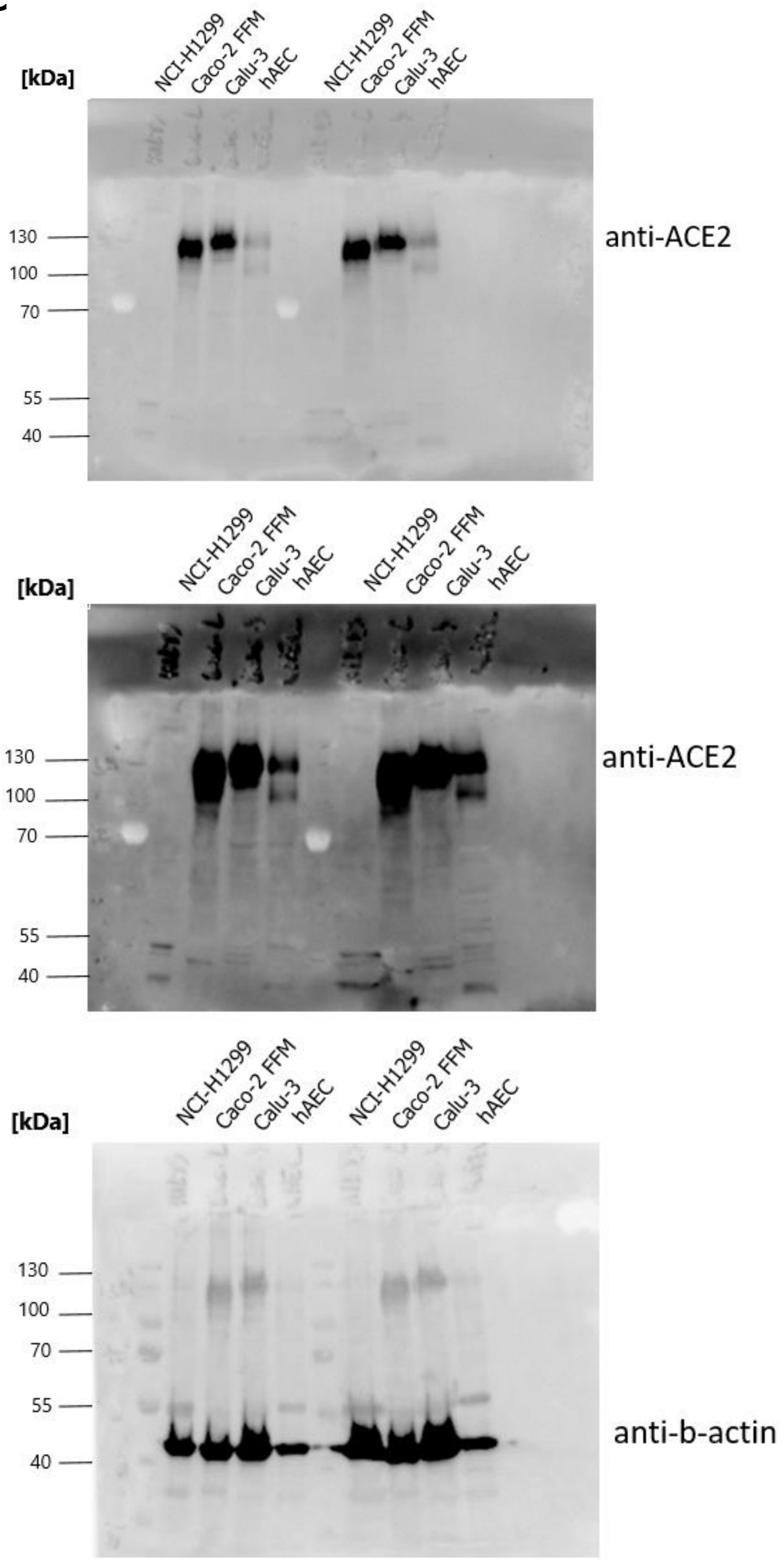
[kDa]
NCI-H1299
Caco-2 FFM
Calu-3
hAEC
130
100
70
55
40
anti-ACE2
anti-b-actin

## Figure S5B

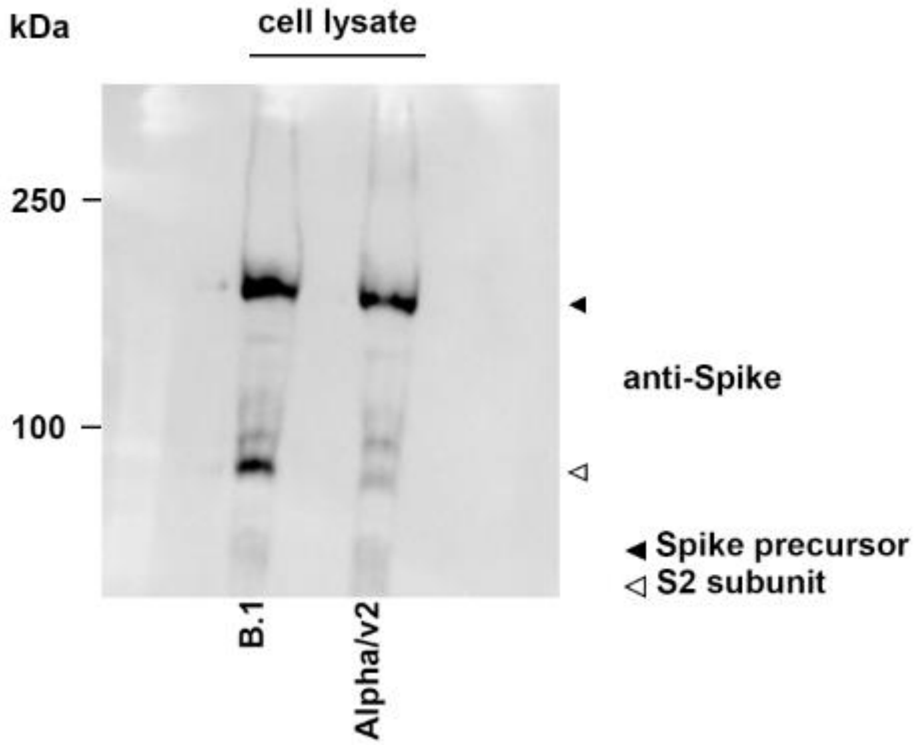

kDa
cell lysate
250
100
anti-Spike
◄ Spike precursor
◁ S2 subunit
B.1
Alpha/v2

**Figure S8A**

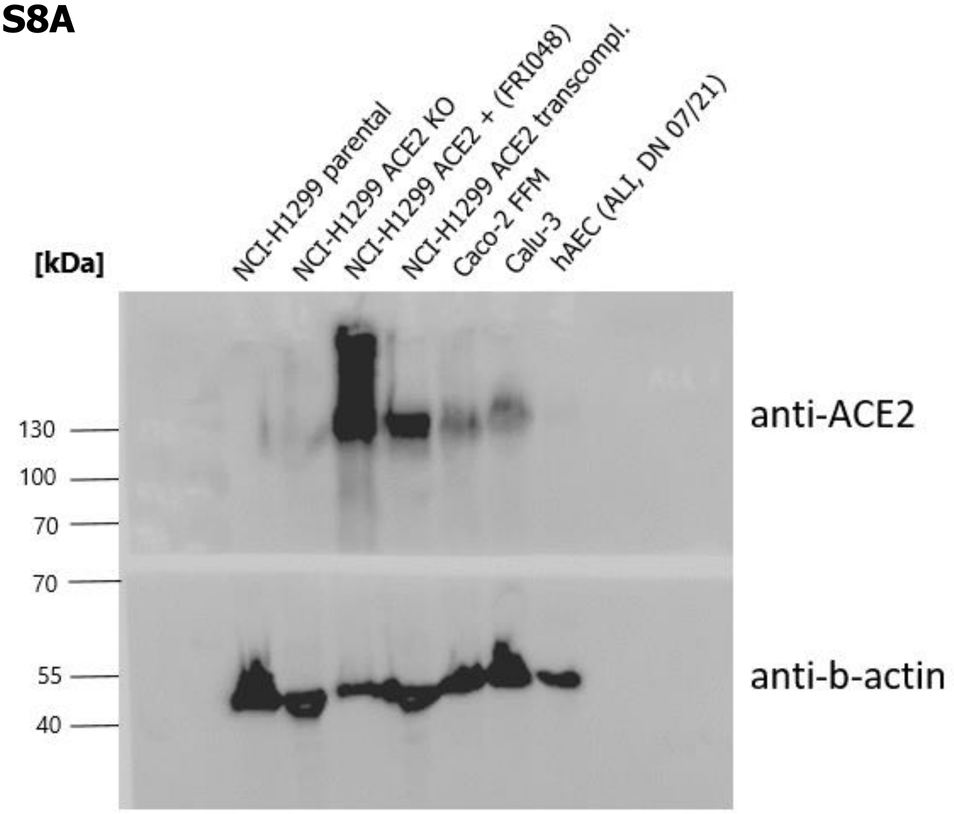
[kDa]
NCI-H1299 parental
NCI-H1299 ACE2 KO
NCI-H1299 ACE2 + (FRI048)
NCI-H1299 ACE2 transcompl.
Caco-2 FFM
Calu-3
hAEC (ALI, DN 07/21)
130
100
70
70
55
40
anti-ACE2
anti-b-actin

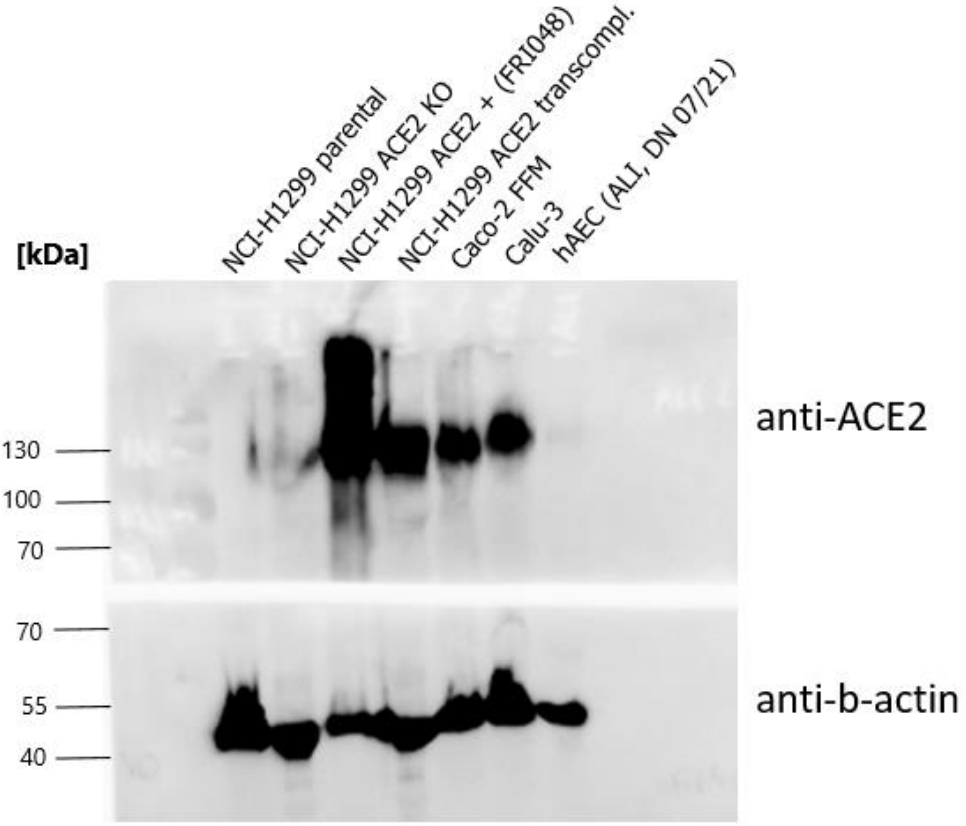
[kDa]
NCI-H1299 parental
NCI-H1299 ACE2 KO
NCI-H1299 ACE2 + (FRI048)
NCI-H1299 ACE2 transcompl.
Caco-2 FFM
Calu-3
hAEC (ALI, DN 07/21)
130
100
70
70
55
40
anti-ACE2
anti-b-actin
